# RNA-Seq analysis in giant pandas reveals the differential expression of multiple genes involved in cataract formation

**DOI:** 10.1186/s12863-021-00996-x

**Published:** 2021-10-27

**Authors:** Yuyan You, Chao Bai, Xuefeng Liu, Yan Lu, Ting Jia, Maohua Xia, Yanqiang Yin, Wei Wang, Yucun Chen, Chenglin Zhang, Yan Liu, Liqin Wang, Tianchun Pu, Tao Ma, Yanhui Liu, Jun Zhou, Lili Niu, Suhui Xu, Yanxia Ni, Xin Hu, Zengshuai Zhang

**Affiliations:** 1 Beijing Key Laboratory of Captive Wildlife Technologies, Beijing Zoo, Beijing, China; 2 Beijing Zoo, Beijing, China; 3 Chongqing Zoo, Chongqing, China; 4 Strait (Fuzhou) Giant Panda Research and Exchange Centers, Fuzhou, China; 5 Chengdu Zoo, Chengdu, China

**Keywords:** Giant panda, endangered mammals, Cataracts, RNA-Seq

## Abstract

**Background:**

The giant panda (*Ailuropoda melanoleuca*) is an endangered mammalian species native to China. Fewer than 2500 giant pandas are known to exist, many of which are bred in captivity as a means to preserve and repopulate the species. Like other captive mammals, giant pandas acquire age-related cataracts, reducing their quality of life. Recent comparative genome-wide methylation analysis revealed 110 differentially methylated genes associated with cataract formation including six also associated with the formation of age-related cataracts in humans.

**Results:**

To investigate the pathological pathway in greater detail, here we used RNA-Seq analysis to investigate the differential expression profiles of genes in three giant pandas with cataracts and three healthy controls. We identified more than 700 differentially expressed genes, 29 of which were selected for further analysis based on their low q-value. We found that many of the genes encoded regulatory and signaling proteins associated with the control of cell growth, migration, differentiation and apoptosis, supporting previous research indicating a key role for apoptosis in cataract formation.

**Conclusion:**

The identification of genes involved in the formation of age-related cataracts could facilitate the development of predictive markers, preventative measures and even new therapies to improve the life of captive animals.

**Supplementary Information:**

The online version contains supplementary material available at 10.1186/s12863-021-00996-x.

## Background

The housing of mammals in zoos and reservations is an efficient strategy to protect endangered species and encourage repopulation, but captive mammals tend to live longer than their wild counterparts and thus experience diseases of ageing, which are uncommon in the wild. One example is the development of age-related cataracts, which are associated with ageing mammals due to the accumulation of oxidative damage in the lens [1]. Cataracts are the main cause of blindness in ageing humans and other primates, as well as companion animals such as dogs and cats [2–4]. They are also prevalent in captive giant pandas (*Ailuropoda melanoleuca*), which live to 15–20 years in the wild but 25–30 years in captivity [5, 6]. Giant pandas 18 or more years old are described as aged because they have reached an equivalent human age of ~ 75. The prevalence of cataracts in the current population of aged giant pandas is ~ 20%.

Age-related cataracts are heritable with significant environmental triggers, including oxidative stress and the resulting accumulation of DNA damage [7–10]. The genes most strongly associated with cataracts therefore include those related to oxidative stress responses, the production of antioxidant enzymes and metabolites, and various DNA repair pathways [11–13]. Previous studies identified changes in DNA methylation associated with cataract formation in several mammals [14–16] and we recently reported that 110 genes with functions relevant to cataract pathogenesis are differentially methylated in giant pandas, including six genes known to be associated with age-related cataracts in humans [17].

Epigenetic modifications such as DNA methylation affect gene expression and therefore control the availability of the corresponding gene products. To gain more insight into the role of differential gene expression in the pathogenesis of age-related cataracts in giant pandas, we took blood samples from three aged giant pandas with cataracts and three healthy controls for RNA-Seq analysis. Following the alignment of reads with the reference genome, we identified expression profiles representing genes expressed exclusively or preferentially in the cataractogenic or healthy samples, and determined the corresponding functional annotations. The identification of genes that are overexpressed or suppressed during the formation of cataracts could lead to the development of new diagnostics, preventative treatments and therapeutic approaches to improve the quality of life for captive giant pandas and other mammals.

## Results

### Samples

Peripheral blood samples were collected from six giant pandas (five females and one male) ranging in age from 19 to 37, with three of the females affected by cataracts and the other specimens defined as healthy based on regular physical examinations (Table 1). The blood samples were assigned to three sample bands: A (affect females), B (unaffected male) and C (unaffected females). These sample bands were subsequently used for comparative transcriptomic analysis.

**Table 1:** Description of giant panda sample donors. Sample band A features the affected females, whereas bands B and C feature the healthy males and females, respectively

Name	Sample band	Spectrum number	Birth year (age)	Status	Sex	Remarks
BD	C3	520	2000 (21)	Healthy	Male	-
YY	C1	362	1990 (31)	Healthy	Female	-
YE	C2	493	1999 (22)	Healthy	Female	-
JN	A3	403	1993 (28)	Age-related cataracts	Female	Died in 2021
LL	A2	320	1986 (32)	Age-related cataracts	Female	Died in 2018
XX	A1	253	1982 (39)	Age-related cataracts	Female	-

### RNA-Seq data processing and quality analysis

Blood samples from all six giant pandas were used to prepare a de novo RNA-Seq dataset with an average of 52.05 ± 5.63 million reads per specimen and an average length of 144.35 ± 1.86 bp after quality control and trimming. For each specimen, ~ 87% of the reads mapped to unique sequences in the reference genome (Supplementary Table S1). Homogeneity distribution analysis and the gene coverage ratio indicated the anticipated distribution of reads within each gene and across different genes (Supplementary Fig. S1a,b) and also confirmed that ~ 44.72% of the reads mapped to exonic regions of the reference sequence (Supplementary Fig. S1c).

### Analysis of gene structure characteristics

**Fig. 1:**
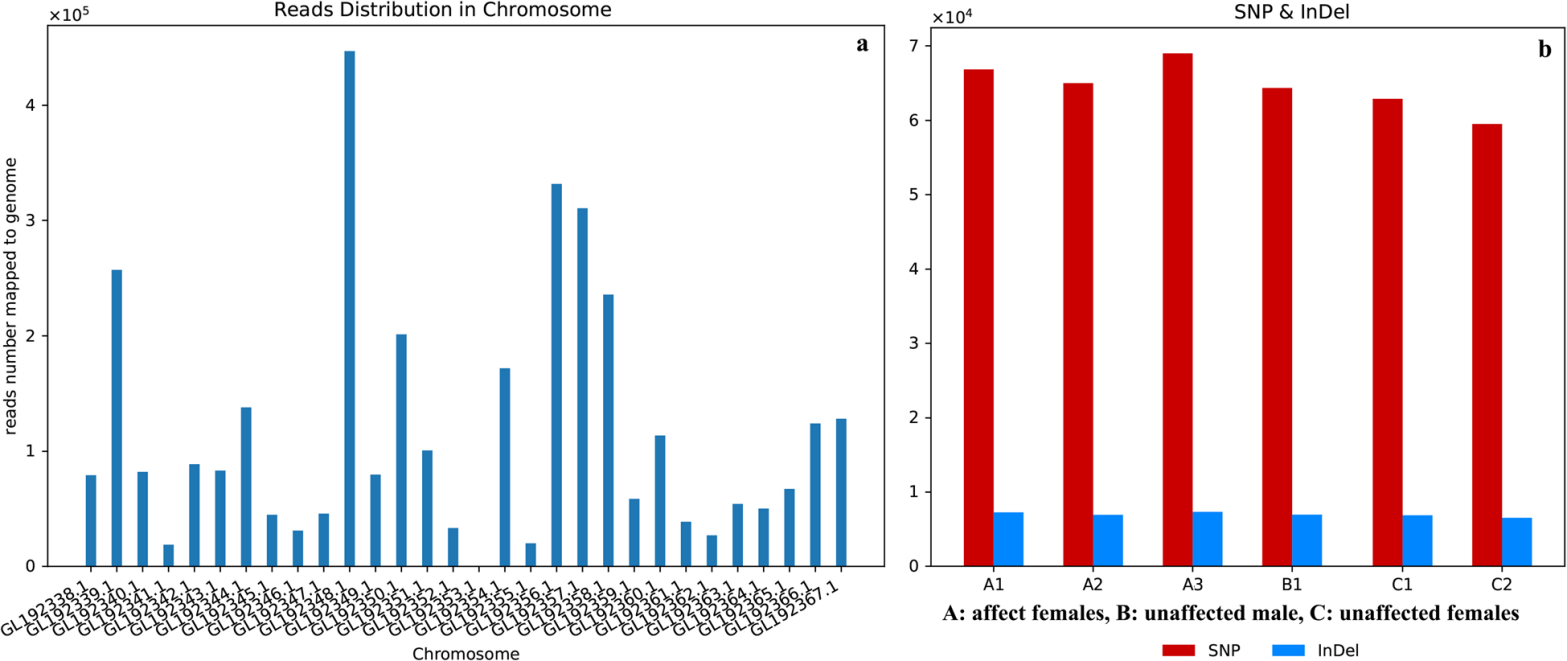
Characteristics of the library mapped to the panda reference genome. (**a**) Distribution of reads across different chromosomes. The x-axis shows the different chromosomes and the y-axis shows the corresponding read number. (**b**) Relative numbers of single-nucleotide polymorphisms (SNPs) and indels in the six pandas

**Fig. 2:**
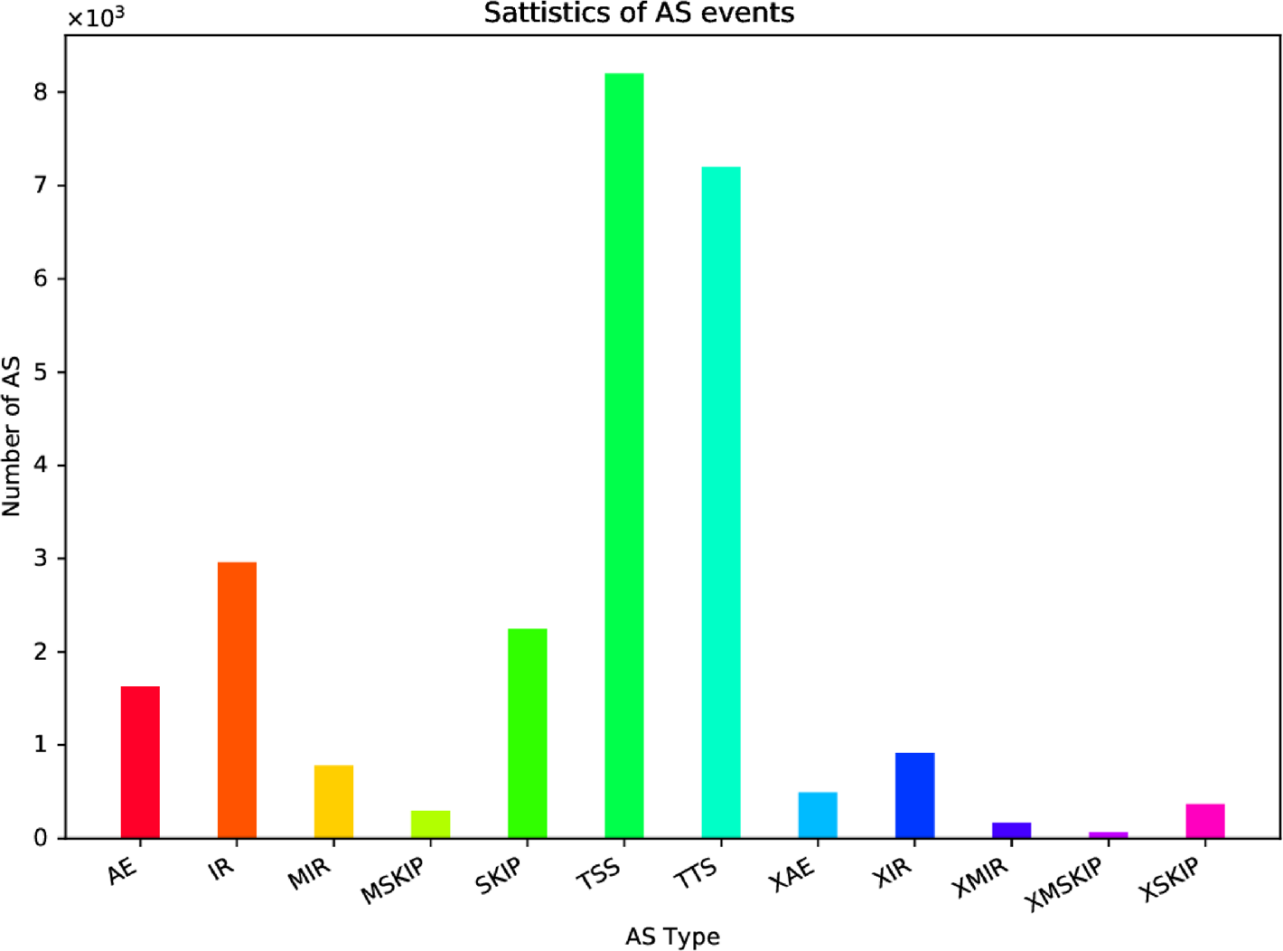
Statistical distribution of different types of RNA processing event. Abbreviations: AE = alternative exon ends, IR = retention of single, MIR = multiple introns, MSKIP = cassette exons, SKIP = exon skipping, TTS = alternative transcription termination site, XAE = approximate AE, XIR = approximate IR, XMIR = approximate MIR, XMSKIP = approximate MSKIP, XSKIP = approximate SKIP

Analysis of the RNA-Seq dataset for gene coverage and chromosome distribution revealed differences in mapping density across different chromosomes, with chromosomes GL192341.1 and GL192355.1 showing a particularly low read density and chromosomes GL192348.1 showing the opposite phenomenon (Fig. 1a). The mapped reads were screened for single-nucleotide polymorphisms (SNPs) and insertion/deletion polymorphisms (indels) revealing 5.9–6.7 × 10^4^ SNPs but < 1 × 10^4^ indels in each sample (Fig. 1b). Among the SNPs, the number of transitions vastly exceeded the number of transversions, and the transitions A > G, C > T, G > A and T > C were particularly abundant (Supplementary Fig. S2). We detected more than 2.5 × 10^4^ alternative mRNA processing events, the most abundant of which were alternative transcriptional start sites (8 × 10^3^) followed by alternative transcriptional stop sites (7 × 10^3^) (Fig. 2). We also identified at least eight new transcript regions representing previously unknown isoforms of known genes, with seven of these new regions mapping to four genes: *GNAO1, OGFOD1, HERPUD1* and *NLRC5* (Supplementary Table S2).

### Analysis of gene expression

**Fig. 3:**
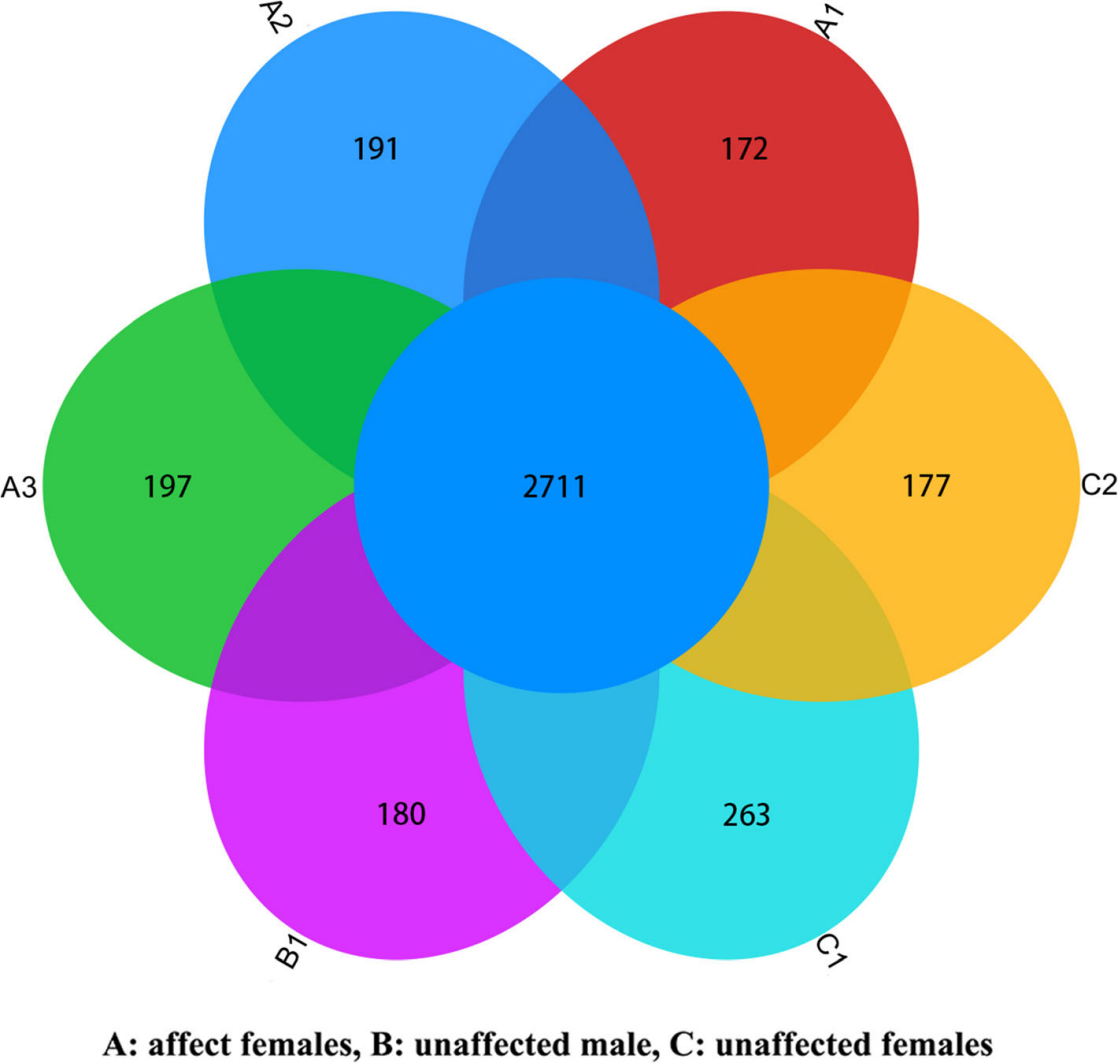
Euler diagram showing the number of differentially expressed genes common to all six pandas and the number of differentially expressed genes in each individual that are not part of this common set

**Fig. 4:**
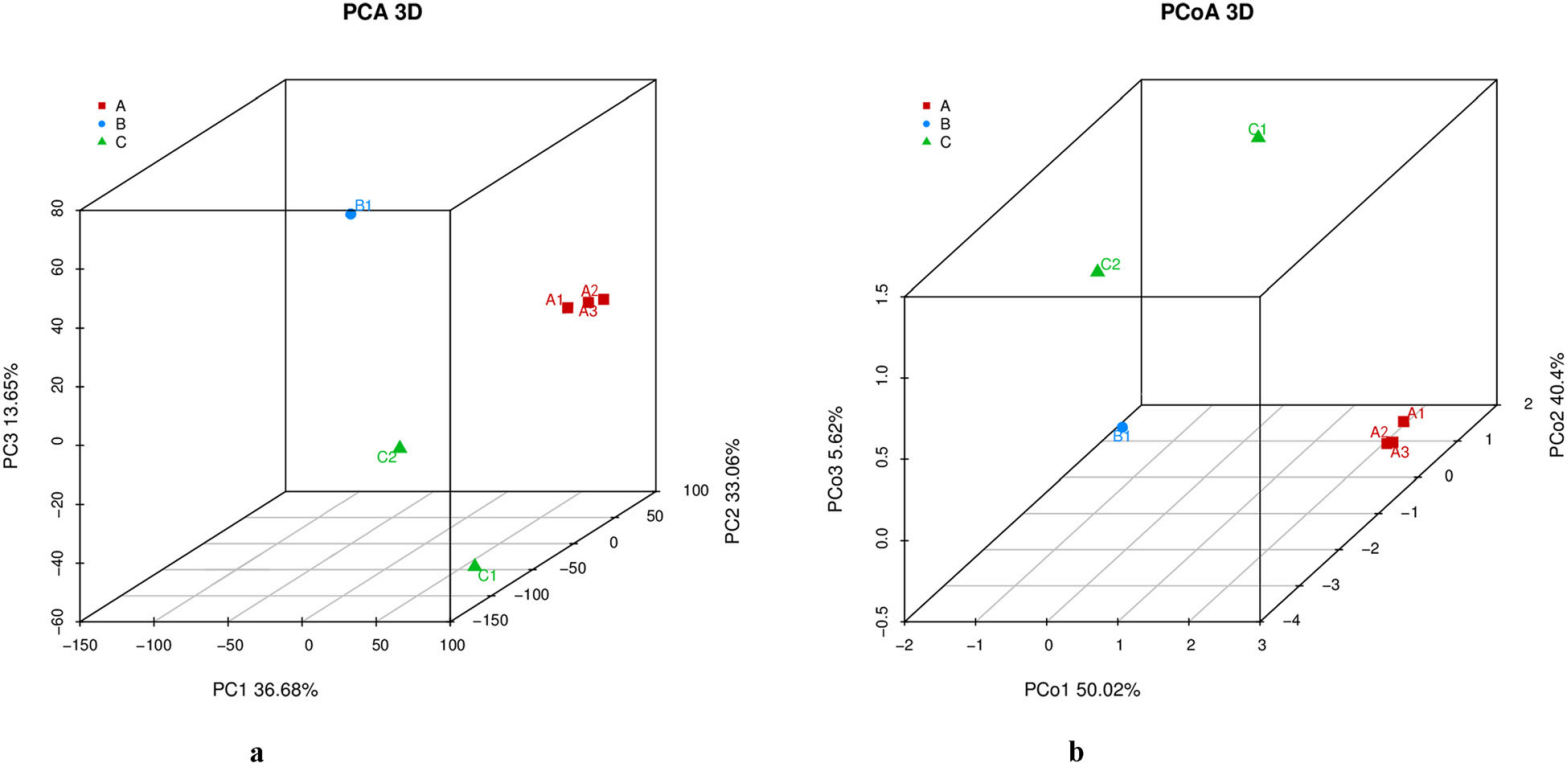
(**a**) Principal component analysis. The three-dimensional plot separates the individual pandas along three principal components. The affected females (red, band A) form one group whereas the unaffected male (blue, band B) and females (green, band C) are scattered. (**b**) Principal coordinates analysis. The three-dimensional plot separates the individual pandas along three principal coordinates. The affected females (red) form one group whereas the unaffected male (blue) and females (green) are scattered. The corresponding two-dimensional plots are shown in Fig. 5

**Fig. 5:**
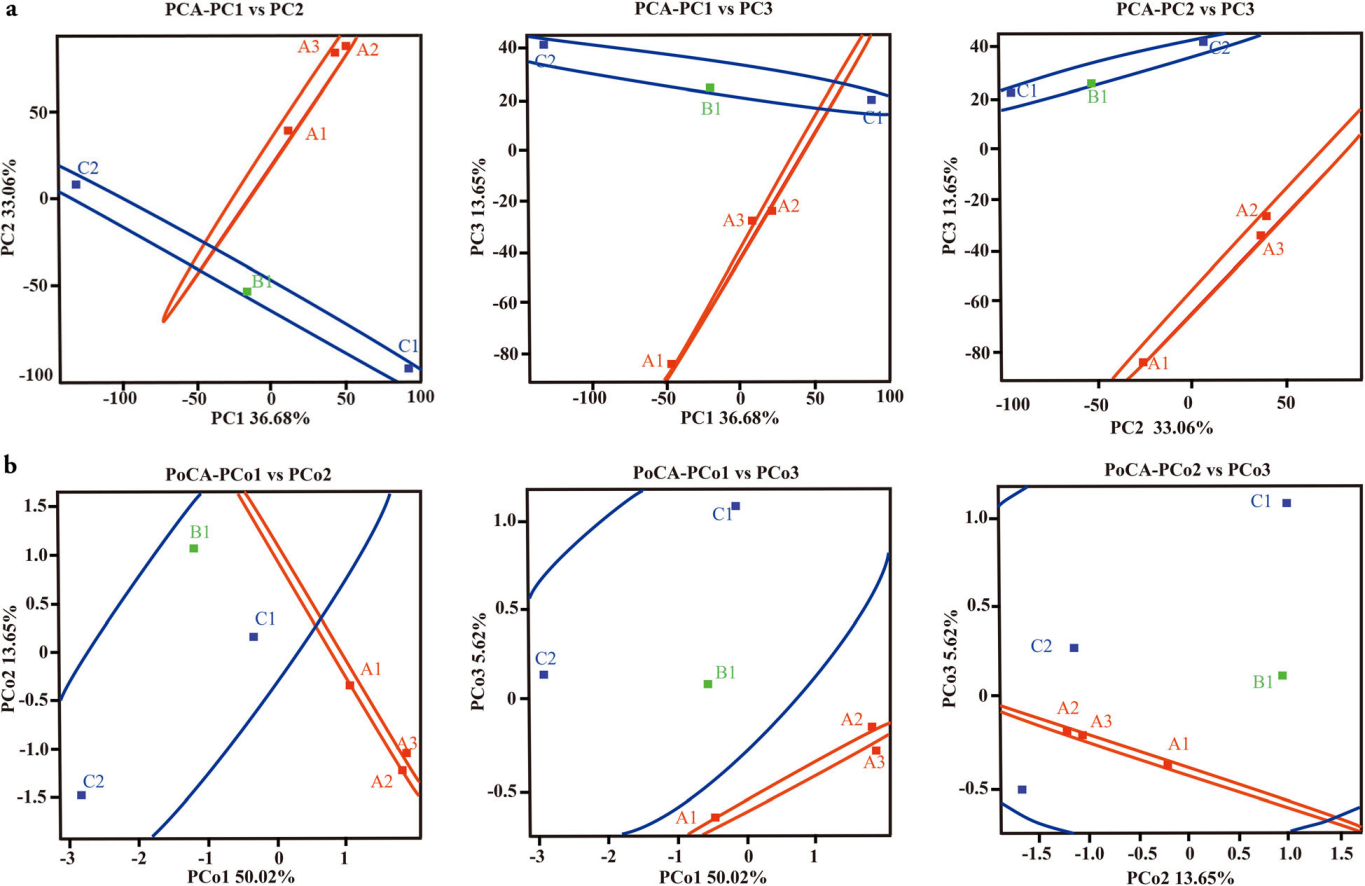
Principal component analysis (**a**) and principal coordinates analysis (**b**). The two-dimensional plots provide more detailed context for the three-dimensional plots shown in Fig. 4a (PCA) and Fig. 4b. (PoCA)

The number of reads mapping to each gene in the reference genome represents the abundance of the corresponding mRNA when corrected for factors such as gene length, sequencing depth and saturation. We also ensured the accuracy of our results by testing for correlation between samples (Supplementary Table S3). We then determined the number of genes that were co-expressed in our samples, revealing a core of 2711 common genes that were universally expressed and a minimum of 172 and a maximum of 263 genes in each animal that were not part of this common set (Fig. 3). PCA indicated that 36.68% of the variation could be explained by PC1, a further 33.06% by PC2, and 13.65% by PC3, primarily separating the affected and unaffected animals but to a lesser extent separating the unaffected male from the unaffected females. Overall, this resulted in the clustering of the affected females together and the dispersion of the other three samples, suggesting the affected females had more in common than any of the unaffected individuals had in common with each other (Fig. 4a). This outcome was broadly supported by PoCA, which revealed that 50.02% of the variation could be explained by PoC1, a further 13.65% by PoC2 and 5.62% by PoC3, again leading to the formation of an affected cluster with the other samples dispersed (Fig. 4b). The individual two-dimensional plots showing the PCA and PoCA data in more detail are provided in Fig. 5.

### Identification and analysis of differentially expressed genes

**Fig. 6:**
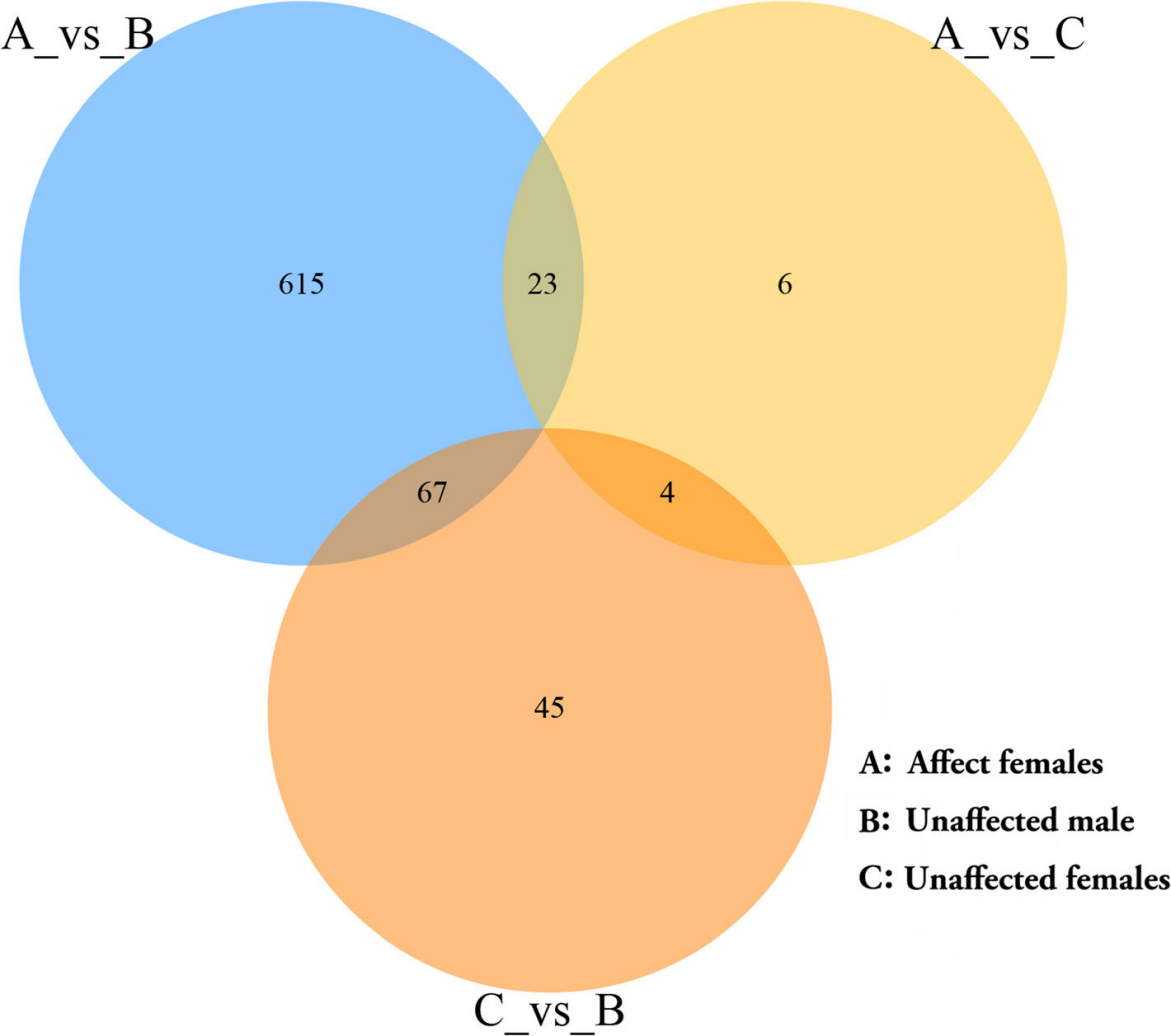
Venn diagram showing the differentially expressed genes when comparing the three bands in three pairwise comparisons. Fig. 7. Scatter plots showing the differentially expressed genes when comparing the three sample bands in three pairwise comparisons. The horizontal and vertical axes represent the log2(TPM) value of two group samples. Each point represents a gene, and the closer each point is to the origin, the lower the expression level. Red represents upregulated genes, green represents downregulated genes, and black represents genes with no difference in expression

**Fig. 7:**
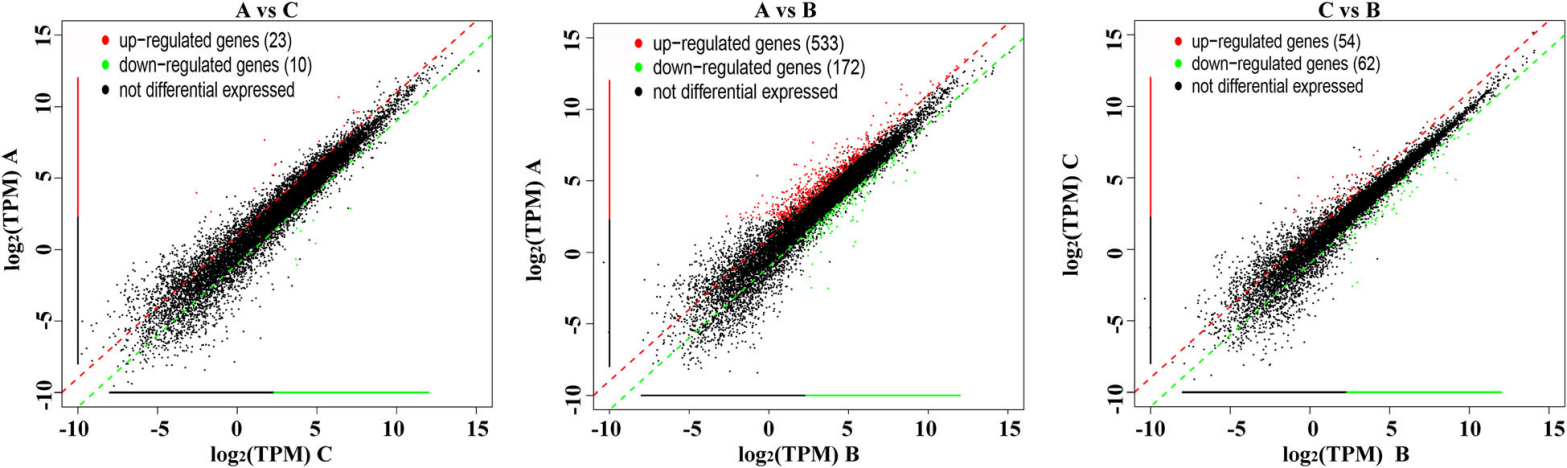
Scatter plots showing the differentially expressed genes when comparing the three sample bands in three pairwise comparisons. The horizontal and vertical axes represent the log2(TPM) value of two group samples. Each point represents a gene, and the closer each point is to the origin, the lower the expression level. Red represents upregulated genes, green represents downregulated genes, and black represents genes with no difference in expression

**Fig. 8:**
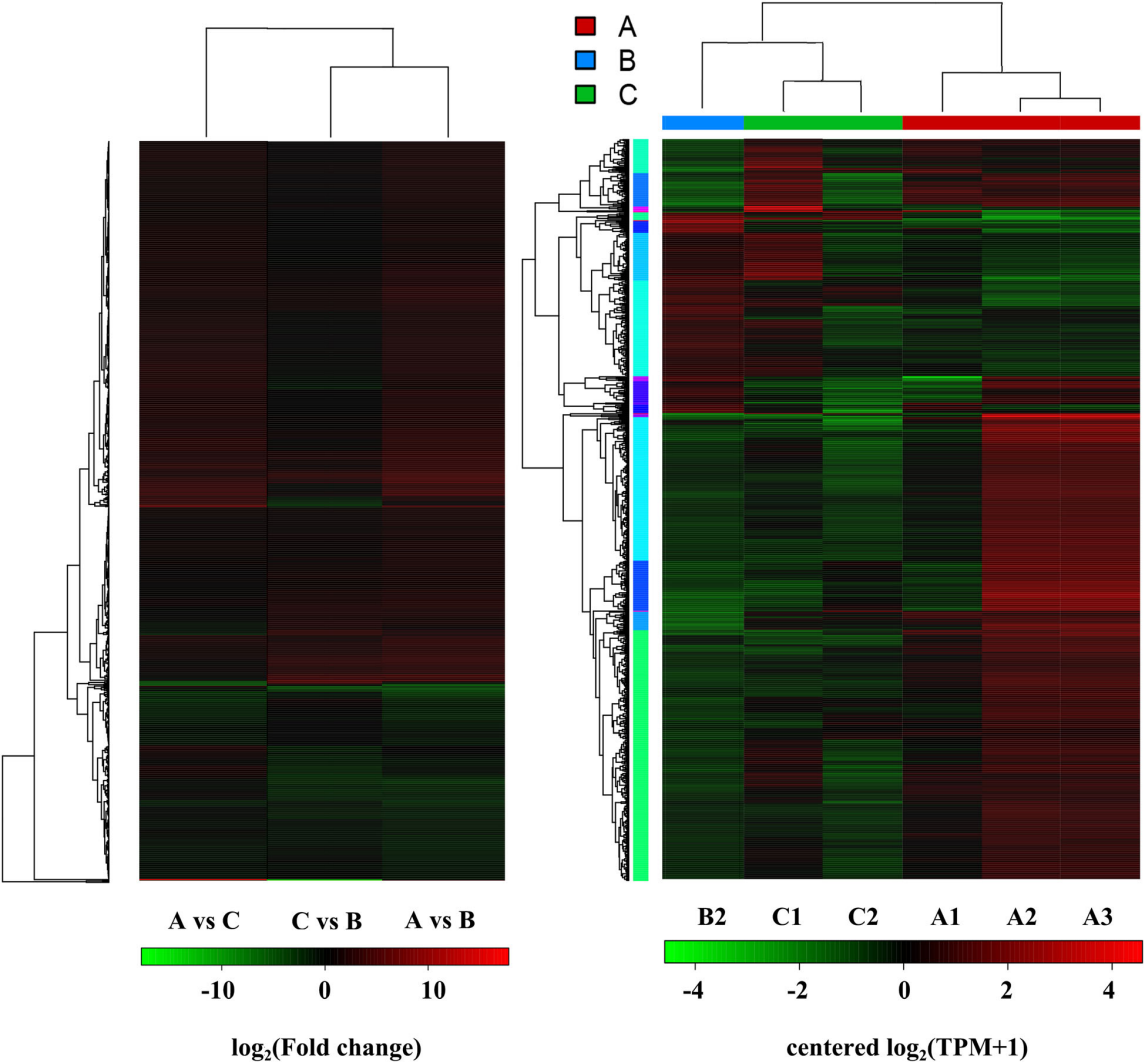
Heat maps to visualize the differentially expressed genes. (**a**) Heat map based on fold change values. Each line represents a gene and each column represents a comparison group (red = upregulated genes and green = downregulated genes, with deeper colors representing higher fold-change values). (**b**) Clustering heat map of differentially expressed genes. Each line represents a gene and each column represents a sample (red = strong expression, green = weak expression). The dendrogram clusters genes with similar expression levels

We observed a much greater number of differentially expressed genes when comparing female pandas with cataracts to the healthy male (A vs B, 705 genes) than when comparing female pandas with cataracts to healthy females (A vs C, 33 genes). However, most of these genes were not differentially expressed when comparing healthy male and female pandas (B vs C, 116 genes), suggesting that the A vs B profile cannot be wholly explained by sex-specific differences in gene expression (Fig. 6). Given that very few genes were differentially expressed when comparing affected and healthy females (A vs C), cataract formation appears to influence a larger number of genes in male than female pandas. Among the 705 differentially expressed genes in the A vs B comparison, 533 were upregulated and 172 downregulated. When visualized as a scatter plot (Fig. 7), it is clear that more genes are upregulated in the healthy male than the affected females and are more likely to show a statistically significant change in expression (Fig. 7b) whereas the 23 upregulated and 10 downregulated genes in the comparison A vs C show limited statistical significance (Fig. 7a). The results are emphasized in the corresponding heat maps (Fig. 8). The comparison of affected females (A) with unaffected pandas of either sex (B + C) revealed 29 genes satisfying q < 0.05 and |log_2_ fold change (FC)| > 1 (Table 2).

**Table 2:** Differentially expressed genes meeting the threshold of low false discovery rate (q < 0.05) and |log2 fold change| > 1

No.	GeneName	result	Transcript id	GeneID	MeanTPM (A)	MeanTPM (B)	log2FoldChange	pValue	qValue	GeneDescription	GO	KEGG	KOG
1	GALNS	up	ENSAMET00000000379	ENSAMEG00000000312	41.27	18.8	1.134360776	0.0000000000000000092189	0.0000000000000027143580	galactosamine (N-acetyl)-6-sulfatase [Source:HGNC Symbol;Acc:HGNC:4122]	GO:0070062;GO:0008484;GO:0008152;GO:0003824;GO:0043890	–	KOG3867-Sulfatase [R]
2	NCF1	down	ENSAMET00000016724	ENSAMEG00000015198	1.536666667	268.47	−7.448812824	0.0000000000000000000000	0.0000000000000000000000	neutrophil cytosolic factor 1 [Source:HGNC Symbol;Acc:HGNC:7660]	GO:0016175;GO:0035091;GO:0055114;GO:0005737;GO:0017124;GO:0043325;GO:0006612;GO:0042554;GO:0005829;GO:0019898;GO:0043020;GO:0014068;GO:0034614;GO:0045741;GO:0045893;GO:0046330;GO:0071276;GO:1900745;GO:0005886	–	–
3	GLG1	down	ENSAMET00000001501	ENSAMEG00000001334	0.283333333	14.76	−5.703048571	0.0000000000000000000000	0.0000000000000000000000	golgi glycoprotein 1 [Source:HGNC Symbol;Acc:HGNC:4316]	GO:0070062;GO:0016020;GO:0005794;GO:0000139;GO:0016021	–	KOG3648-Golgi apparatus protein (cysteine-rich fibroblast growth factor receptor)[U]
4	C12orf56	up	ENSAMET00000019892	ENSAMEG00000018106	14.68666667	2.5	2.554506994	0.0000000000000000000000	0.0000000000000000000000	chromosome 12 open reading frame 56 [Source:HGNC Symbol;Acc:HGNC:26967]	–	–	–
5	AZIN1	down	ENSAMET00000016178	ENSAMEG00000014703	25.47	61.03	−1.260719631	0.0000000000000001900961	0.0000000000000502788186	antizyme inhibitor 1 [Source:RefSeq peptide;Acc:NP_001278387]	GO:0006596;GO:0042978;GO:0043085;GO:0003824;GO:0042177;GO:1902269;GO:0005634	–	KOG0622-Ornithine decarboxylase [E]
6	RCAN1	down	ENSAMET00000005825	ENSAMEG00000005295	0.0001	8.21	−16.3250946	0.0000000000000165267939	0.0000000000036324030796	regulator of calcineurin 1 [Source:HGNC Symbol;Acc:HGNC:3040]	GO:0033173;GO:0019722;GO:0003676;GO:0005622	K17901-ko04921 Oxytocin signaling pathway;ko04919 Thyroid hormone signaling pathway	KOG4019-Calcineurin-mediated signaling pathway inhibitor DSCR1[TR]
7	RASD1	up	ENSAMET00000002517	ENSAMEG00000002311	15.45333333	0.76	4.345774837	0.0000000000025012050313	0.0000000003903130451290	ras related dexamethasone induced 1 [Source:HGNC Symbol;Acc:HGNC:15828]	GO:0016529;GO:0003924;GO:0005525;GO:0000166;GO:0007165;GO:0016020	–	KOG0395-Ras-related GTPase [R]
8	CYSLTR1	up	ENSAMET00000021716	ENSAMEG00000019893	19.67666667	8.01	1.296611693	0.0000000000033418073494	0.0000000005112637616410	cysteinyl leukotriene receptor 1 [Source:HGNC Symbol;Acc:HGNC:17451]	GO:0004974;GO:0061737;GO:0007186;GO:0004930;GO:0016021;GO:0016020;GO:0002437;GO:0006816;GO:0006935;GO:0007166;GO:0005887	K04322-ko04020 Calcium signaling pathway;ko04080 Neuroactive ligand-receptor interaction	–
9	EGR1	up	ENSAMET00000018184	ENSAMEG00000016544	63.94333333	4.81	3.732685154	0.0000000000130159154540	0.0000000017816961461400	early growth response 1 [Source:HGNC Symbol;Acc:HGNC:3238]	GO:0005737;GO:0005654;GO:0005634;GO:2000182;GO:0098759;GO:0090090;GO:0071480;GO:0071310;GO:0070498;GO:0061418;GO:0050725;GO:0046886;GO:0045944;GO:0045893;GO:0045080;GO:0044849;GO:0042981;GO:0035914;GO:0033233;GO:0032868;GO:0030509;GO:0030217;GO:0009749;GO:0006366;GO:0006355;GO:0002931;GO:0001666;GO:0000122;GO:1990841;GO:0044212;GO:0044729;GO:0043565;GO:0035035;GO:0010385;GO:0008270;GO:0003700;GO:0003677;GO:0001077;GO:0000982;GO:0000977;GO:0000976;GO:0006351;GO:0003676;GO:0046872	K09203-ko05020 Prion diseases;ko04933 AGE-RAGE signaling pathway in diabetic complications;ko05166 HTLV-I infection	KOG1721-FOG: Zn-finger [R]
10	RANBP9	up	ENSAMET00000000514	ENSAMEG00000000415	8.37	1.31	2.675660811	0.0000000000223677291954	0.0000000029087367841100	RAN binding protein 9 [Source:HGNC Symbol;Acc:HGNC:13727]	GO:0005737;GO:0005634;GO:0070373;GO:0019899	–	KOG1477-SPRY domain-containing proteins [R]
11	UCHL1	down	ENSAMET00000018946	ENSAMEG00000017217	2.423333333	8.48	− 1.807071401	0.0000000003728989939510	0.0000000401316469007000	ubiquitin C-terminal hydrolase L1 [Source:HGNC Symbol;Acc:HGNC:12513]	GO:0006511;GO:1904115;GO:0016874;GO:0016787;GO:0016579;GO:0006508;GO:0036459;GO:0008234;GO:0008233;GO:0004843;GO:0005622;GO:0004197;GO:0008242;GO:0031625;GO:0031694;GO:0043130;GO:0043407;GO:0005654;GO:0005737;GO:0005829;GO:0005886;GO:0070062	–	KOG1415-Ubiquitin C-terminal hydrolase UCHL1[O]
12	TNFSF12	up	ENSAMET00000018386	ENSAMEG00000016729	8.51	0.0001	16.37687151	0.0000000004630328987080	0.0000000481708558955999	TNF superfamily member 12 [Source:HGNC Symbol;Acc:HGNC:11927]	GO:0005164;GO:0006955;GO:0016020;GO:0045732;GO:0097191;GO:2001238;GO:0005576;GO:0048471	–	–
13	SERPINB10	up	ENSAMET00000016640	ENSAMEG00000015156	58.52	13.83	2.081128613	0.0000000032653933058800	0.0000002715024303890000	serpin family B member 10 [Source:HGNC Symbol;Acc:HGNC:8942]	GO:0005615;GO:0005634;GO:0005829	–	KOG2392-Serpin [V]
14	SPPL2B	up	ENSAMET00000006059	ENSAMEG00000005486	65.44666667	26.61	1.298351204	0.0000000042149703973800	0.0000003425761096409990	signal peptide peptidase like 2B [Source:HGNC Symbol;Acc:HGNC:30627]	GO:0071556;GO:0030660;GO:0071458;GO:0016020;GO:0015629;GO:0010008;GO:0005886;GO:0005813;GO:0005765;GO:0005654;GO:0050776;GO:0033619;GO:0031293;GO:0006509;GO:0042803;GO:0042500;GO:0004190;GO:0016021	–	KOG2442-Uncharacterized conserved protein, contains PA domain [R]
15	PTP4A3	up	ENSAMET00000001697	ENSAMEG00000001550	38.58666667	5.66	2.769228463	0.0000000599264476394000	0.0000035422432402000000	protein tyrosine phosphatase type IVA, member 3 [Source:HGNC Symbol;Acc:HGNC:9636]	GO:1900746;GO:0043542;GO:0043117;GO:0007219;GO:0006470;GO:0016311;GO:0016791;GO:0008138;GO:0006355;GO:1901224;GO:1904951;GO:0005634;GO:0005737;GO:1990830	–	KOG2836-Protein tyrosine phosphatase IVA1[T]
16	FOS	up	ENSAMET00000020143	ENSAMEG00000018335	1639.143333	71.19	4.525123604	0.0000011001521433000000	0.0000455381278412000000	Fos proto-oncogene, AP-1 transcription factor subunit [Source:HGNC Symbol;Acc:HGNC:3796]	GO:0005654;GO:0005667;GO:0005634;GO:0071277;GO:0060395;GO:0045944;GO:0045893;GO:0045672;GO:0042493;GO:0035994;GO:0035914;GO:0034614;GO:0031668;GO:0007399;GO:0007179;GO:0006355;GO:0070412;GO:0044212;GO:0008134;GO:0003700;GO:0003682;GO:0003677;GO:0001077;GO:0000979;GO:0000978;GO:0006357;GO:0006366;GO:0001102;GO:0001190;GO:0046982;GO:0071276;GO:0032993;GO:0035976	K04379-ko04010 MAPK signaling pathway;ko04668 TNF signaling pathway;ko04024 cAMP signaling pathway;ko04210 Apoptosis;ko04620 Toll-like receptor signaling pathway;ko04660 T cell receptor signaling pathway;ko04662 B cell receptor signaling pathway;ko04915 Estrogen signaling pathway;ko04917 Prolactin signaling pathway;ko04921 Oxytocin signaling pathway;ko04725 Cholinergic synapse;ko04728 Dopaminergic synapse;ko04380 Osteoclast differentiation;ko04713 Circadian entrainment;ko05200 Pathways in cancer;ko05231 Choline metabolism in cancer;ko05210 Colorectal cancer;ko05224 Breast cancer;ko05323 Rheumatoid arthritis;ko05031 Amphetamine addiction;ko05132 Salmonella infection;ko05133 Pertussis;ko05166 HTLV-I infection;ko05161 Hepatitis B;ko05168 Herpes simplex infection;ko05140 Leishmaniasis;ko05142 Chagas disease (American trypanosomiasis);ko01522 Endocrine resistance	KOG1414-Transcriptional activator FOSB/c-Fos and related bZIP transcription factors [K]
17	ARHGAP21	up	ENSAMET00000007161	ENSAMEG00000006453	5.533333333	2.03	1.446669108	0.0000060927451965700000	0.000197666	Rho GTPase activating protein 21 [Source:HGNC Symbol;Acc:HGNC:23725]	GO:0005096;GO:0043547;GO:0007165;GO:0030054;GO:0015629;GO:0005886;GO:0005794;GO:0072384;GO:0051684;GO:0051683;GO:0007030	–	–
18	FOSB	up	ENSAMET00000013608	ENSAMEG00000012405	204.76	6.83	4.905904523	0.0000086438020328300000	0.000266574	FosB proto-oncogene, AP-1 transcription factor subunit [Source:HGNC Symbol;Acc:HGNC:3797]	GO:0043231;GO:0005654;GO:0071277;GO:0045944;GO:0003677;GO:0001077;GO:0000978;GO:0006357;GO:0005634;GO:0003700;GO:0006366;GO:0006355	K09029-ko04380 Osteoclast differentiation;ko05030 Cocaine addiction;ko05031 Amphetamine addiction;ko05034 Alcoholism	KOG1414-Transcriptional activator FOSB/c-Fos and related bZIP transcription factors [K]
19	IFI27L2	up	ENSAMET00000006889	ENSAMEG00000006288	798.83	337.07	1.244840284	0.0000096178956521000000	0.000292568	interferon alpha inducible protein 27 like 2 [Source:HGNC Symbol;Acc:HGNC:19753]	GO:0016021;GO:0016020	–	–
20	ANXA3	down	ENSAMET00000005999	ENSAMEG00000005375	13.09666667	44.12	−1.752233124	0.0000856456143253000000	0.001808525	annexin A3 [Source:HGNC Symbol;Acc:HGNC:541]	GO:0070062;GO:0042581;GO:0030670;GO:0016020;GO:0005829;GO:0005886;GO:0005737;GO:0051091;GO:0045766;GO:0043312;GO:0042742;GO:0006909;GO:0010595;GO:0048306;GO:0005544;GO:0004859;GO:0043086;GO:0005509	–	KOG0819-Annexin [U]
21	CLIC4	up	ENSAMET00000011061	ENSAMEG00000010080	14.94333333	6.36	1.232403328	0.000125571	0.002467927	chloride intracellular channel 4 [Source:HGNC Symbol;Acc:HGNC:13518]	GO:0070062;GO:0048471;GO:0045177;GO:0030496;GO:0016363;GO:0015630;GO:0009986;GO:0005911;GO:0005886;GO:0005902;GO:0005829;GO:0005739;GO:0005813;GO:0005737;GO:0005622;GO:0071277;GO:0061299;GO:0048754;GO:0035264;GO:0030336;GO:0030216;GO:0009566;GO:0007035;GO:0001886;GO:0001525;GO:0034707;GO:0005254;GO:0006821;GO:1902476;GO:0034765;GO:0005244;GO:0006811;GO:0006810;GO:0016020	–	KOG1422-Intracellular Cl- channel CLIC, contains GST domain [P]
22	COL2A1	up	ENSAMET00000014144	ENSAMEG00000012885	15.23	6.57	1.212950666	0.000132805	0.002568066	collagen type II alpha 1 chain [Source:HGNC Symbol;Acc:HGNC:2200]	GO:0005669;GO:0006352;GO:0046982;GO:0003700;GO:0006355;GO:0000118;GO:0016575;GO:0032041;GO:0004407;GO:0070932;GO:0016569;GO:0006325;GO:0006351;GO:0005634;GO:0016787;GO:0031012;GO:0005585;GO:0060272;GO:0030199;GO:0007605;GO:0007601;GO:0001501;GO:0048407;GO:0005201;GO:0042289;GO:0005576	–	KOG3544-Collagens (type IV and type XIII), and related proteins [W]
23	DUSP22	up	ENSAMET00000006552	ENSAMEG00000005967	41.65333333	15.57	1.419663008	0.00020624	0.003683723	dual specificity phosphatase 22 [Source:HGNC Symbol;Acc:HGNC:16077]	GO:0000122;GO:0035335;GO:0016311;GO:0016791;GO:0008138;GO:0006470	–	KOG1716-Dual specificity phosphatase [V]
24	EPCAM	down	ENSAMET00000006851	ENSAMEG00000006223	2.763333333	5.91	−1.096748529	0.000258111	0.004446881	epithelial cell adhesion molecule [Source:HGNC Symbol;Acc:HGNC:11529]	GO:0070062;GO:0016328;GO:0016323;GO:0016324;GO:0009986;GO:0005923;GO:0005886;GO:2000648;GO:2000048;GO:0048863;GO:0045944;GO:0023019;GO:0008284;GO:0001657;GO:0032403;GO:0016020;GO:0016021	–	–
25	PDE4A	up	ENSAMET00000012636	ENSAMEG00000011455	6.78	2.24	1.597786541	0.000451773	0.006951735	phosphodiesterase 4A [Source:HGNC Symbol;Acc:HGNC:8780]	GO:0004114;GO:0008081;GO:0016787;GO:0007165;GO:0046872;GO:0048471;GO:0016020;GO:0005886;GO:0005737;GO:0005829;GO:0005654;GO:0043949;GO:0035690;GO:0010738;GO:0007608;GO:0006198;GO:0030552;GO:0004115	–	KOG3689-Cyclic nucleotide phosphodiesterase [T]
26	MYBL1	down	ENSAMET00000013645	ENSAMEG00000012401	4.073333333	15.41	−1.919585077	0.000777204	0.010437403	MYB proto-oncogene like 1 [Source:HGNC Symbol;Acc:HGNC:7547]	GO:0045944;GO:0001077;GO:0000978;GO:0006366;GO:0006355;GO:0003677	–	KOG0048-Transcription factor, Myb superfamily [K]
27	STEAP3	up	ENSAMET00000009325	ENSAMEG00000008497	18.19	6.95	1.38806066	0.000805245	0.010721711	STEAP3 metalloreductase [Source:HGNC Symbol;Acc:HGNC:24592]	GO:0005771;GO:0005737;GO:0009306;GO:0016020;GO:0016021	–	–
28	UNC119	up	ENSAMET00000005420	ENSAMEG00000004930	111.16	50.33	1.143147237	0.002244887	0.022926346	unc-119 lipid binding chaperone [Source:HGNC Symbol;Acc:HGNC:12565]	GO:0051233;GO:0045171;GO:0005813;GO:0000922;GO:0061098;GO:0042953;GO:0008289;GO:0007399;GO:0000281	–	KOG4037-Photoreceptor synaptic vesicle protein HRG4/UNC-119[UT]
29	ENG	up	ENSAMET00000016623	ENSAMEG00000015119	36.99666667	8.75	2.08004037	0.003054818	0.028699842	endoglin [Source:HGNC Symbol;Acc:HGNC:3349]	GO:0007165;GO:0016020;GO:0016021;GO:0005072;GO:0005114;GO:0005534;GO:0005539;GO:0034713;GO:0036122;GO:0042803;GO:0050431;GO:0001300;GO:0001525;GO:0001569;GO:0001570;GO:0001934;GO:0001947;GO:0003148;GO:0003198;GO:0003203;GO:0003208;GO:0003209;GO:0003222;GO:0003273;GO:0006355;GO:0007507;GO:0010629;GO:0010862;GO:0017015;GO:0022009;GO:0030336;GO:0030513;GO:0031953;GO:0035912;GO:0045766;GO:0045944;GO:0048745;GO:0048844;GO:0048845;GO:0055009;GO:0097084;GO:1905007;GO:1905065;GO:1905222;GO:0005615;GO:0005925;GO:0009897;GO:0043235;GO:0072563	–	–

### Functional annotation of differentially expressed genes

**Fig. 9:**
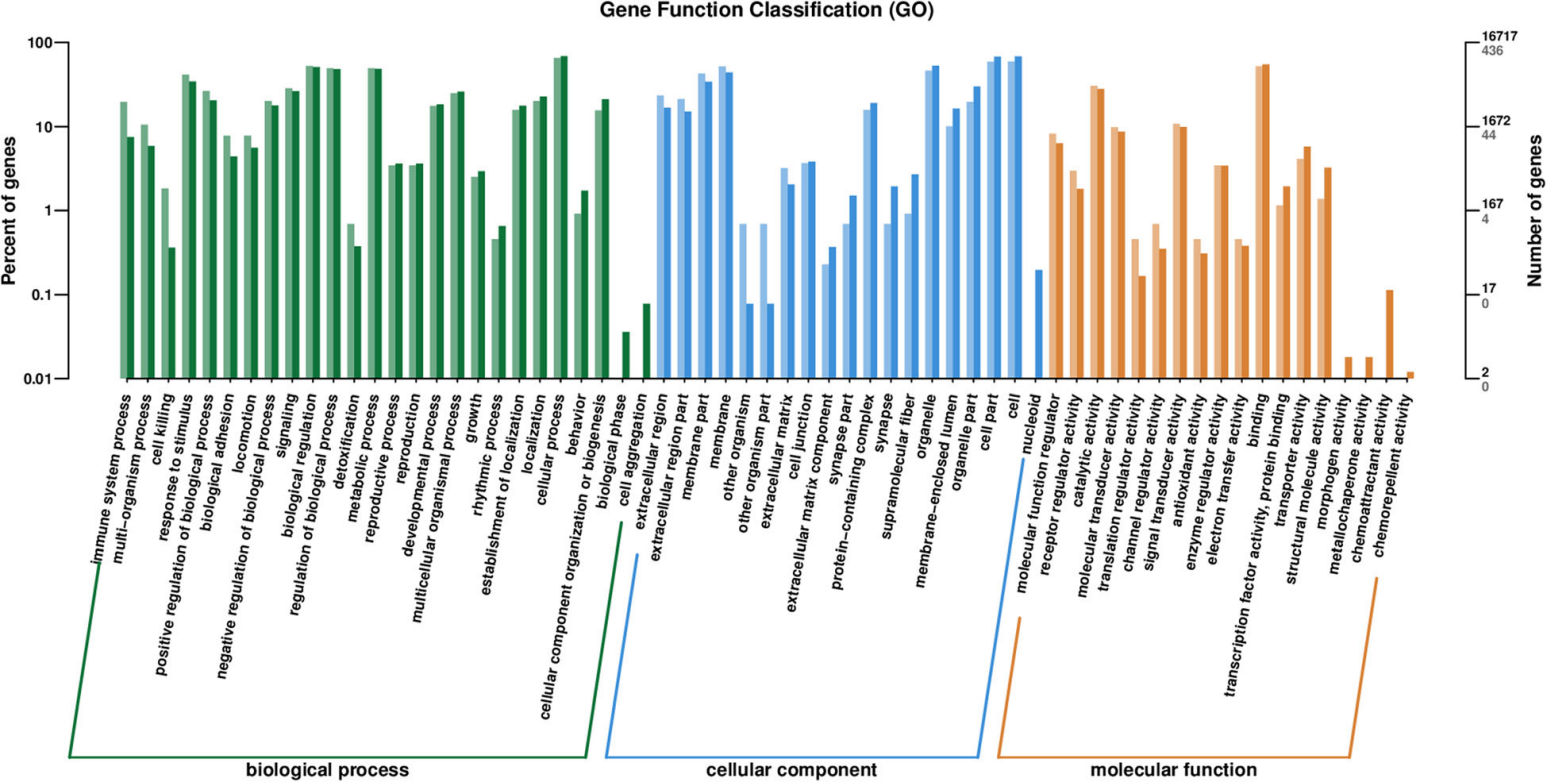
Gene Ontology categories of differentially expressed genes. The x-axis shows classifications and the y-axes show percentage of total classified genes (left) and total number of genes (right). Different colors represent the three different domains of biological process (green), cellular component (blue) and molecular function (yellow). Light bars represent differentially expressed genes, and dark bars represent all genes

**Fig. 10:**
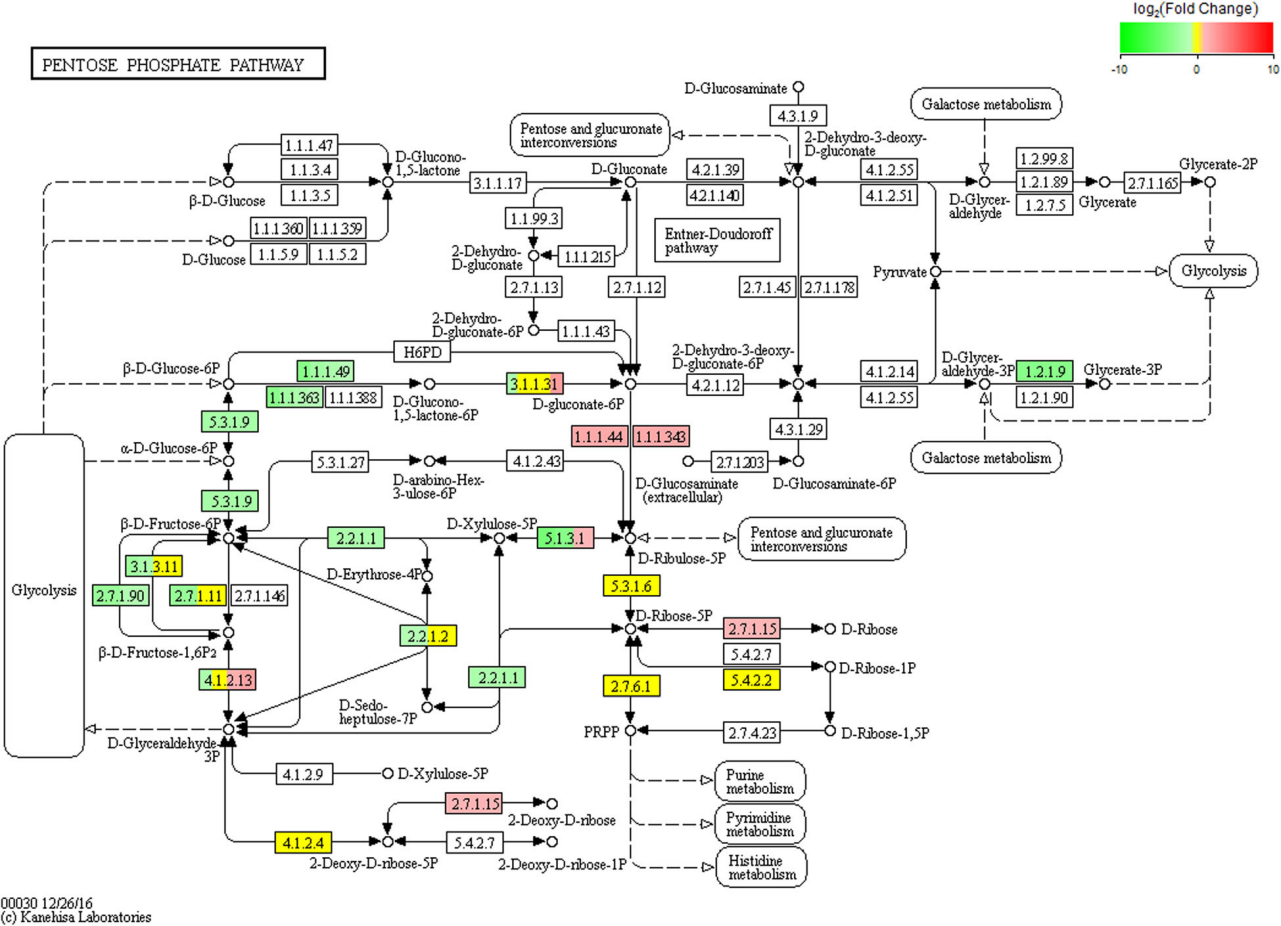
Differential representation of metabolic pathways. The rectangular nodes represent gene products (enzymes or regulatory factors) and the circular nodes represent metabolites. The rounded white boxes indicate linked pathways. Red indicates upregulated genes and green indicates downregulated genes, with deeper coloring indicating a greater fold change in expression

**Fig. 11:**
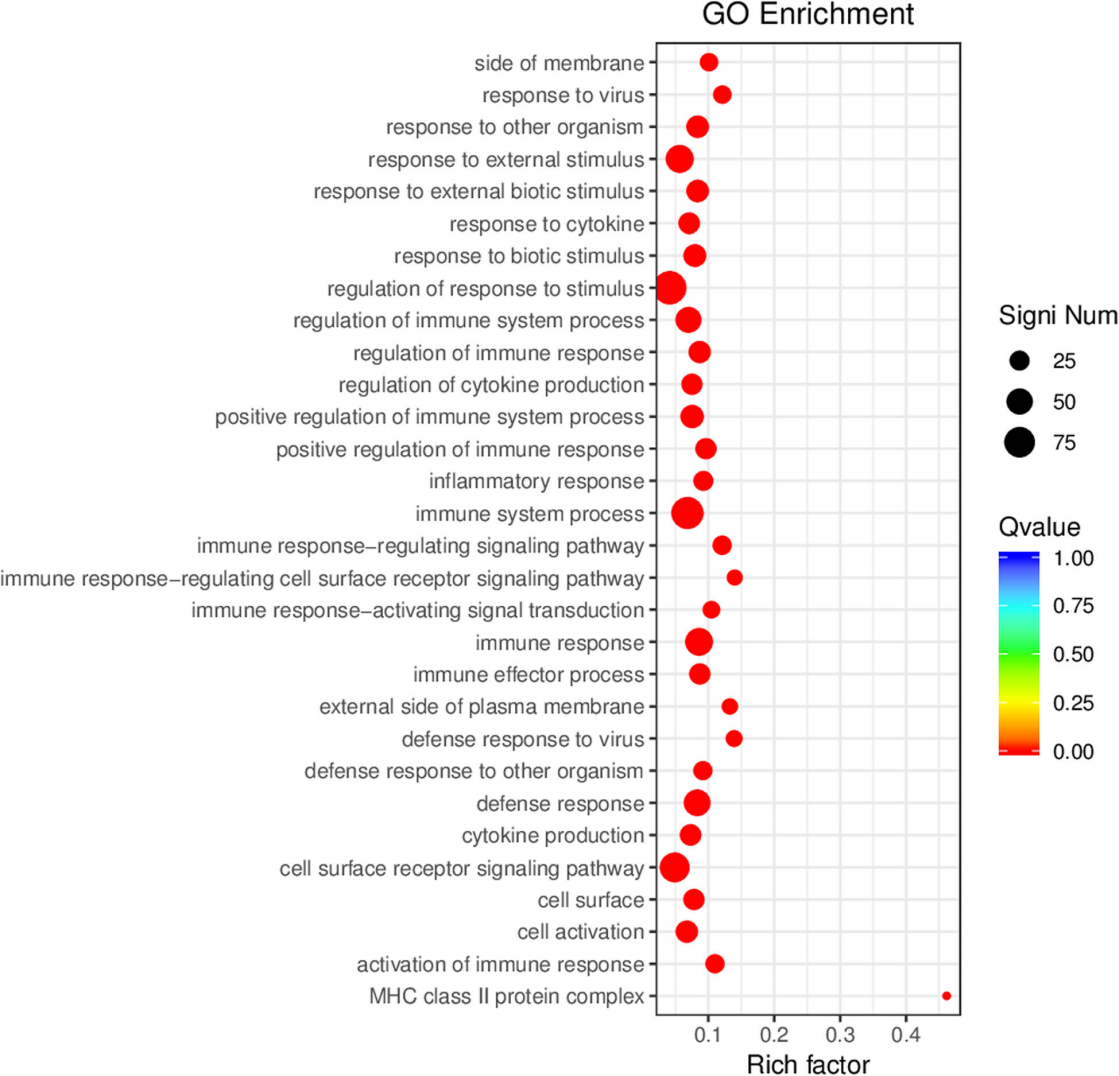
Significant enrichment functional scatter plot. The vertical axis represents the functional annotation, and the horizontal axis represents the Rich factor of that function. The q-value is represented by the color of the dots, and the number of differentially expressed genes representing each function is shown by the size of the dots. Only the 30 highest enrichments are shown

The biological functions and related pathways of the differentially expressed genes were investigated by screening the sequences against the GO and KEGG databases, as well as the Clusters of Orthologous Groups of proteins (COG) and euKaryotic Ortholog Groups (KOG) maintained by the NCBI. GO annotations revealed that the differentially expressed genes represented a wide range of biological processes (111 genes), cellular components (73 genes) and molecular functions (5 genes). Strongly represented biological process categories (accounting for > 10% of the genes) included *cellular process*, *metabolic process*, *biological regulation*/*regulation of a biological process* (particularly positive regulation), and *response to stimulus*. With few exceptions, the functional profile of the differentially expressed genes mirrored that of the total gene catalog, although the differentially expressed genes were overrepresented in the categories *immune system process* and *multi-organism process* but underrepresented in the categories *biological phase* and *cell aggregation* (Fig. 9). There was little difference in terms of cellular component categories, with the exception of the differentially expressed genes being unrepresented in the category *nucleoid*. However, one of the most abundant categories among the differentially expressed and complete gene catalog was *protein-containing complex*, and preliminary protein network analysis revealed that the genes most strongly upregulated in the A vs B comparison also tended to be more likely to interact with other proteins and also tended to have more connections (data not shown). In terms of molecular functions, there was again little difference between the differentially expressed genes and complete gene catalog, with the exception of the differentially expressed genes being unrepresented in the categories involving *morphogen*, *metallochaperone* and *chemoattractant*/*chemorepellant* activity (Fig. 9). Interestingly, several genes encoding enzymes involved in histidine metabolism were included among the differentially expressed pathways revealed when screening KEGG (Fig. 10). The analysis of GO categories that were enriched among the differentially expressed genes revealed strong hits for immunity and defense-related functions, which showed the highest Rich factors (Table 3, Fig. 11). Visualization of the biological, cellular and molecular functions by means of directed acyclic graphs revealed that 45 genes differed between the healthy and unhealthy pandas (Supplementary Fig. S3).

**Table 3:** Functional enrichment analysis based on the representation of GO terms

GO.ID	Term	Ontology	Significant	Annotated	Pvalue	Qvalue	Signi_id	Signi_symbol
GO:0031982	vesicle	cellular component	84/287	2493/14310	0.00000041	0.0004	ENSAMEG00000000015, ENSAMEG00000000093, ENSAMEG00000000312, ENSAMEG00000000943, ENSAMEG00000001070, ENSAMEG00000001092, ENSAMEG00000001276, ENSAMEG00000001502, ENSAMEG00000002069, ENSAMEG00000002535, ENSAMEG00000002581, ENSAMEG00000002718, ENSAMEG00000002732, ENSAMEG00000003442, ENSAMEG00000003627, ENSAMEG00000003820, ENSAMEG00000004013, ENSAMEG00000004196, ENSAMEG00000004441, ENSAMEG00000004680, ENSAMEG00000005486, ENSAMEG00000006014, ENSAMEG00000006082, ENSAMEG00000006368, ENSAMEG00000007046, ENSAMEG00000007327, ENSAMEG00000007468, ENSAMEG00000007631, ENSAMEG00000008008, ENSAMEG00000008407, ENSAMEG00000008497, ENSAMEG00000008620, ENSAMEG00000008800, ENSAMEG00000009105, ENSAMEG00000009193, ENSAMEG00000010113, ENSAMEG00000010219, ENSAMEG00000010536, ENSAMEG00000010841, ENSAMEG00000010991, ENSAMEG00000011403, ENSAMEG00000011494, ENSAMEG00000011509, ENSAMEG00000011617, ENSAMEG00000011687, ENSAMEG00000011750, ENSAMEG00000011820, ENSAMEG00000011842, ENSAMEG00000012016, ENSAMEG00000012043, ENSAMEG00000012457, ENSAMEG00000012482, ENSAMEG00000012483, ENSAMEG00000012511, ENSAMEG00000012590, ENSAMEG00000012708, ENSAMEG00000012743, ENSAMEG00000012804, ENSAMEG00000012943, ENSAMEG00000013803, ENSAMEG00000013869, ENSAMEG00000013931, ENSAMEG00000013980, ENSAMEG00000014605, ENSAMEG00000014724, ENSAMEG00000015157, ENSAMEG00000015221, ENSAMEG00000015326, ENSAMEG00000015564, ENSAMEG00000015938, ENSAMEG00000016015, ENSAMEG00000016143, ENSAMEG00000016396, ENSAMEG00000016446, ENSAMEG00000016736, ENSAMEG00000016795, ENSAMEG00000017062, ENSAMEG00000017184, ENSAMEG00000017862, ENSAMEG00000017964, ENSAMEG00000018199, ENSAMEG00000019907, ENSAMEG00000020287, ENSAMEG00000023442	PDZK1IP1, NUDT14, GALNS, LPL, MINDY1, SLC1A4, ZNRF1, ECM1, KCNQ1, ANPEP, HNMT, SCPEP1, PLOD3, CRYZ, EPS8, CMBL, KCTD12, SLPI, GAS6, NANS, SPPL2B, RAB32, RHOQ, MRAS, TMEM8A, TRIP10, SMPDL3A, SLC7A8, OAF, SLC1A5, STEAP3, CYBRD1, DGKH, AHCY, DMXL2, SLA2, GNPDA1, VIM, HSPB1, HIP1, F5, FBP1, SEPT5, COMT, SERPINE2, HAX1, LYZ, APOH, ANGPTL6, FAM168B, BLVRB, PLD3, CTSZ, SOGA1, GPX1, LAMB2, TUBB6, C3orf58, TIMP1, FES, A2M, RRAS, IGSF8, NAAA, ITSN1, GAA, SGSH, MICALL2, PRADC1, HFE, ACTG1, PEBP1, DLG4, EFHD1, TNFSF13, TMEM175, ARRB1, APP, ABCA3, PTGR1, CDC42BPB, ENDOD1, KCNE3, ATP6
GO:0005576	extracellular region	cellular component	91/287	2809/14310	0.00000064	0.0004	ENSAMEG00000000015, ENSAMEG00000000093, ENSAMEG00000000312, ENSAMEG00000000640, ENSAMEG00000000943, ENSAMEG00000001070, ENSAMEG00000001092, ENSAMEG00000001502, ENSAMEG00000001948, ENSAMEG00000002444, ENSAMEG00000002535, ENSAMEG00000002581, ENSAMEG00000002718, ENSAMEG00000002732, ENSAMEG00000003068, ENSAMEG00000003442, ENSAMEG00000003627, ENSAMEG00000003820, ENSAMEG00000004013, ENSAMEG00000004196, ENSAMEG00000004399, ENSAMEG00000004441, ENSAMEG00000004680, ENSAMEG00000004836, ENSAMEG00000006082, ENSAMEG00000006368, ENSAMEG00000007046, ENSAMEG00000007327, ENSAMEG00000007340, ENSAMEG00000007342, ENSAMEG00000007468, ENSAMEG00000007631, ENSAMEG00000008008, ENSAMEG00000008407, ENSAMEG00000008620, ENSAMEG00000008780, ENSAMEG00000009105, ENSAMEG00000009193, ENSAMEG00000009998, ENSAMEG00000010219, ENSAMEG00000010536, ENSAMEG00000010602, ENSAMEG00000010841, ENSAMEG00000011403, ENSAMEG00000011494, ENSAMEG00000011617, ENSAMEG00000011687, ENSAMEG00000011820, ENSAMEG00000011842, ENSAMEG00000012016, ENSAMEG00000012043, ENSAMEG00000012457, ENSAMEG00000012482, ENSAMEG00000012483, ENSAMEG00000012511, ENSAMEG00000012590, ENSAMEG00000012708, ENSAMEG00000012743, ENSAMEG00000012804, ENSAMEG00000012869, ENSAMEG00000012943, ENSAMEG00000013536, ENSAMEG00000013869, ENSAMEG00000013931, ENSAMEG00000013980, ENSAMEG00000014335, ENSAMEG00000014605, ENSAMEG00000014906, ENSAMEG00000014908, ENSAMEG00000015156, ENSAMEG00000015157, ENSAMEG00000015221, ENSAMEG00000015429, ENSAMEG00000015548, ENSAMEG00000015564, ENSAMEG00000015938, ENSAMEG00000016015, ENSAMEG00000016143, ENSAMEG00000016224, ENSAMEG00000016446, ENSAMEG00000016729, ENSAMEG00000016736, ENSAMEG00000016820, ENSAMEG00000017184, ENSAMEG00000017422, ENSAMEG00000017862, ENSAMEG00000017964, ENSAMEG00000018103, ENSAMEG00000018199, ENSAMEG00000019907, ENSAMEG00000023442	PDZK1IP1, NUDT14, GALNS, HSPB6, LPL, MINDY1, SLC1A4, ECM1, DKK2, CRTAP, ANPEP, HNMT, SCPEP1, PLOD3, MMP19, CRYZ, EPS8, CMBL, KCTD12, SLPI, PPFIBP2, GAS6, NANS, TIMP4, RHOQ, MRAS, TMEM8A, TRIP10, ZP1, SDC2, SMPDL3A, SLC7A8, OAF, SLC1A5, CYBRD1, ENSAMEG00000008780, AHCY, DMXL2, EMILIN2, GNPDA1, VIM, PLBD1, HSPB1, F5, FBP1, COMT, SERPINE2, LYZ, APOH, ANGPTL6, FAM168B, BLVRB, PLD3, CTSZ, SOGA1, GPX1, LAMB2, TUBB6, C3orf58, CFP, TIMP1, ECM2, A2M, RRAS, IGSF8, ENSAMEG00000014335, NAAA, CCL14, ENSAMEG00000014908, SERPINB10, GAA, SGSH, ELN, ENSAMEG00000015548, PRADC1, HFE, ACTG1, PEBP1, CD36, EFHD1, TNFSF12, TNFSF13, IL25, APP, F13A1, ABCA3, PTGR1, CCDC80, CDC42BPB, ENDOD1, ATP6
GO:0044421	extracellular region part	cellular component	84/287	2524/14310	0.00000070	0.0004	ENSAMEG00000000015, ENSAMEG00000000093, ENSAMEG00000000312, ENSAMEG00000000943, ENSAMEG00000001070, ENSAMEG00000001092, ENSAMEG00000001502, ENSAMEG00000001948, ENSAMEG00000002444, ENSAMEG00000002535, ENSAMEG00000002581, ENSAMEG00000002718, ENSAMEG00000002732, ENSAMEG00000003068, ENSAMEG00000003442, ENSAMEG00000003627, ENSAMEG00000003820, ENSAMEG00000004013, ENSAMEG00000004196, ENSAMEG00000004399, ENSAMEG00000004441, ENSAMEG00000004680, ENSAMEG00000006082, ENSAMEG00000006368, ENSAMEG00000007046, ENSAMEG00000007327, ENSAMEG00000007340, ENSAMEG00000007342, ENSAMEG00000007468, ENSAMEG00000007631, ENSAMEG00000008008, ENSAMEG00000008407, ENSAMEG00000008620, ENSAMEG00000008780, ENSAMEG00000009105, ENSAMEG00000009193, ENSAMEG00000009998, ENSAMEG00000010219, ENSAMEG00000010536, ENSAMEG00000010602, ENSAMEG00000010841, ENSAMEG00000011403, ENSAMEG00000011494, ENSAMEG00000011617, ENSAMEG00000011687, ENSAMEG00000011820, ENSAMEG00000011842, ENSAMEG00000012016, ENSAMEG00000012043, ENSAMEG00000012457, ENSAMEG00000012482, ENSAMEG00000012483, ENSAMEG00000012511, ENSAMEG00000012590, ENSAMEG00000012708, ENSAMEG00000012743, ENSAMEG00000012804, ENSAMEG00000012869, ENSAMEG00000012943, ENSAMEG00000013536, ENSAMEG00000013869, ENSAMEG00000013931, ENSAMEG00000013980, ENSAMEG00000014335, ENSAMEG00000014605, ENSAMEG00000015156, ENSAMEG00000015157, ENSAMEG00000015221, ENSAMEG00000015429, ENSAMEG00000015564, ENSAMEG00000015938, ENSAMEG00000016015, ENSAMEG00000016143, ENSAMEG00000016224, ENSAMEG00000016446, ENSAMEG00000016736, ENSAMEG00000017184, ENSAMEG00000017422, ENSAMEG00000017862, ENSAMEG00000017964, ENSAMEG00000018103, ENSAMEG00000018199, ENSAMEG00000019907, ENSAMEG00000023442	PDZK1IP1, NUDT14, GALNS, LPL, MINDY1, SLC1A4, ECM1, DKK2, CRTAP, ANPEP, HNMT, SCPEP1, PLOD3, MMP19, CRYZ, EPS8, CMBL, KCTD12, SLPI, PPFIBP2, GAS6, NANS, RHOQ, MRAS, TMEM8A, TRIP10, ZP1, SDC2, SMPDL3A, SLC7A8, OAF, SLC1A5, CYBRD1, ENSAMEG00000008780, AHCY, DMXL2, EMILIN2, GNPDA1, VIM, PLBD1, HSPB1, F5, FBP1, COMT, SERPINE2, LYZ, APOH, ANGPTL6, FAM168B, BLVRB, PLD3, CTSZ, SOGA1, GPX1, LAMB2, TUBB6, C3orf58, CFP, TIMP1, ECM2, A2M, RRAS, IGSF8, ENSAMEG00000014335, NAAA, SERPINB10, GAA, SGSH, ELN, PRADC1, HFE, ACTG1, PEBP1, CD36, EFHD1, TNFSF13, APP, F13A1, ABCA3, PTGR1, CCDC80, CDC42BPB, ENDOD1, ATP6
GO:0005615	extracellular space	cellular component	79/287	2352/14310	0.00000120	0.0005	ENSAMEG00000000015, ENSAMEG00000000093, ENSAMEG00000000312, ENSAMEG00000000943, ENSAMEG00000001070, ENSAMEG00000001092, ENSAMEG00000001502, ENSAMEG00000001948, ENSAMEG00000002444, ENSAMEG00000002535, ENSAMEG00000002581, ENSAMEG00000002718, ENSAMEG00000002732, ENSAMEG00000003068, ENSAMEG00000003442, ENSAMEG00000003627, ENSAMEG00000003820, ENSAMEG00000004013, ENSAMEG00000004196, ENSAMEG00000004399, ENSAMEG00000004441, ENSAMEG00000004680, ENSAMEG00000006082, ENSAMEG00000006368, ENSAMEG00000007046, ENSAMEG00000007327, ENSAMEG00000007468, ENSAMEG00000007631, ENSAMEG00000008008, ENSAMEG00000008407, ENSAMEG00000008620, ENSAMEG00000008780, ENSAMEG00000009105, ENSAMEG00000009193, ENSAMEG00000009998, ENSAMEG00000010219, ENSAMEG00000010536, ENSAMEG00000010602, ENSAMEG00000010841, ENSAMEG00000011403, ENSAMEG00000011494, ENSAMEG00000011617, ENSAMEG00000011687, ENSAMEG00000011820, ENSAMEG00000011842, ENSAMEG00000012016, ENSAMEG00000012043, ENSAMEG00000012457, ENSAMEG00000012482, ENSAMEG00000012483, ENSAMEG00000012511, ENSAMEG00000012590, ENSAMEG00000012708, ENSAMEG00000012743, ENSAMEG00000012804, ENSAMEG00000012943, ENSAMEG00000013869, ENSAMEG00000013931, ENSAMEG00000013980, ENSAMEG00000014335, ENSAMEG00000014605, ENSAMEG00000015156, ENSAMEG00000015157, ENSAMEG00000015221, ENSAMEG00000015564, ENSAMEG00000015938, ENSAMEG00000016015, ENSAMEG00000016143, ENSAMEG00000016224, ENSAMEG00000016446, ENSAMEG00000016736, ENSAMEG00000017184, ENSAMEG00000017422, ENSAMEG00000017862, ENSAMEG00000017964, ENSAMEG00000018103, ENSAMEG00000018199, ENSAMEG00000019907, ENSAMEG00000023442	PDZK1IP1, NUDT14, GALNS, LPL, MINDY1, SLC1A4, ECM1, DKK2, CRTAP, ANPEP, HNMT, SCPEP1, PLOD3, MMP19, CRYZ, EPS8, CMBL, KCTD12, SLPI, PPFIBP2, GAS6, NANS, RHOQ, MRAS, TMEM8A, TRIP10, SMPDL3A, SLC7A8, OAF, SLC1A5, CYBRD1, ENSAMEG00000008780, AHCY, DMXL2, EMILIN2, GNPDA1, VIM, PLBD1, HSPB1, F5, FBP1, COMT, SERPINE2, LYZ, APOH, ANGPTL6, FAM168B, BLVRB, PLD3, CTSZ, SOGA1, GPX1, LAMB2, TUBB6, C3orf58, TIMP1, A2M, RRAS, IGSF8, ENSAMEG00000014335, NAAA, SERPINB10, GAA, SGSH, PRADC1, HFE, ACTG1, PEBP1, CD36, EFHD1, TNFSF13, APP, F13A1, ABCA3, PTGR1, CCDC80, CDC42BPB, ENDOD1, ATP6
GO:0043230	extracellular organelle	cellular component	63/287	1929/14310	0.00004900	0.013	ENSAMEG00000000015, ENSAMEG00000000093, ENSAMEG00000000312, ENSAMEG00000000943, ENSAMEG00000001070, ENSAMEG00000001092, ENSAMEG00000001502, ENSAMEG00000002535, ENSAMEG00000002581, ENSAMEG00000002718, ENSAMEG00000002732, ENSAMEG00000003442, ENSAMEG00000003627, ENSAMEG00000003820, ENSAMEG00000004013, ENSAMEG00000004196, ENSAMEG00000004441, ENSAMEG00000004680, ENSAMEG00000006082, ENSAMEG00000006368, ENSAMEG00000007046, ENSAMEG00000007327, ENSAMEG00000007468, ENSAMEG00000007631, ENSAMEG00000008008, ENSAMEG00000008407, ENSAMEG00000008620, ENSAMEG00000009105, ENSAMEG00000010219, ENSAMEG00000010536, ENSAMEG00000010841, ENSAMEG00000011403, ENSAMEG00000011494, ENSAMEG00000011617, ENSAMEG00000011687, ENSAMEG00000011820, ENSAMEG00000011842, ENSAMEG00000012016, ENSAMEG00000012043, ENSAMEG00000012457, ENSAMEG00000012482, ENSAMEG00000012483, ENSAMEG00000012511, ENSAMEG00000012590, ENSAMEG00000012708, ENSAMEG00000012743, ENSAMEG00000012943, ENSAMEG00000013869, ENSAMEG00000013931, ENSAMEG00000013980, ENSAMEG00000014605, ENSAMEG00000015157, ENSAMEG00000015221, ENSAMEG00000015564, ENSAMEG00000016015, ENSAMEG00000016143, ENSAMEG00000016446, ENSAMEG00000016736, ENSAMEG00000017184, ENSAMEG00000017964, ENSAMEG00000018199, ENSAMEG00000019907, ENSAMEG00000023442	PDZK1IP1, NUDT14, GALNS, LPL, MINDY1, SLC1A4, ECM1, ANPEP, HNMT, SCPEP1, PLOD3, CRYZ, EPS8, CMBL, KCTD12, SLPI, GAS6, NANS, RHOQ, MRAS, TMEM8A, TRIP10, SMPDL3A, SLC7A8, OAF, SLC1A5, CYBRD1, AHCY, GNPDA1, VIM, HSPB1, F5, FBP1, COMT, SERPINE2, LYZ, APOH, ANGPTL6, FAM168B, BLVRB, PLD3, CTSZ, SOGA1, GPX1, LAMB2, TUBB6, TIMP1, A2M, RRAS, IGSF8, NAAA, GAA, SGSH, PRADC1, ACTG1, PEBP1, EFHD1, TNFSF13, APP, PTGR1, CDC42BPB, ENDOD1, ATP6
GO:1903561	extracellular vesicle	cellular component	63/287	1929/14310	0.00004900	0.013	ENSAMEG00000000015, ENSAMEG00000000093, ENSAMEG00000000312, ENSAMEG00000000943, ENSAMEG00000001070, ENSAMEG00000001092, ENSAMEG00000001502, ENSAMEG00000002535, ENSAMEG00000002581, ENSAMEG00000002718, ENSAMEG00000002732, ENSAMEG00000003442, ENSAMEG00000003627, ENSAMEG00000003820, ENSAMEG00000004013, ENSAMEG00000004196, ENSAMEG00000004441, ENSAMEG00000004680, ENSAMEG00000006082, ENSAMEG00000006368, ENSAMEG00000007046, ENSAMEG00000007327, ENSAMEG00000007468, ENSAMEG00000007631, ENSAMEG00000008008, ENSAMEG00000008407, ENSAMEG00000008620, ENSAMEG00000009105, ENSAMEG00000010219, ENSAMEG00000010536, ENSAMEG00000010841, ENSAMEG00000011403, ENSAMEG00000011494, ENSAMEG00000011617, ENSAMEG00000011687, ENSAMEG00000011820, ENSAMEG00000011842, ENSAMEG00000012016, ENSAMEG00000012043, ENSAMEG00000012457, ENSAMEG00000012482, ENSAMEG00000012483, ENSAMEG00000012511, ENSAMEG00000012590, ENSAMEG00000012708, ENSAMEG00000012743, ENSAMEG00000012943, ENSAMEG00000013869, ENSAMEG00000013931, ENSAMEG00000013980, ENSAMEG00000014605, ENSAMEG00000015157, ENSAMEG00000015221, ENSAMEG00000015564, ENSAMEG00000016015, ENSAMEG00000016143, ENSAMEG00000016446, ENSAMEG00000016736, ENSAMEG00000017184, ENSAMEG00000017964, ENSAMEG00000018199, ENSAMEG00000019907, ENSAMEG00000023442	PDZK1IP1, NUDT14, GALNS, LPL, MINDY1, SLC1A4, ECM1, ANPEP, HNMT, SCPEP1, PLOD3, CRYZ, EPS8, CMBL, KCTD12, SLPI, GAS6, NANS, RHOQ, MRAS, TMEM8A, TRIP10, SMPDL3A, SLC7A8, OAF, SLC1A5, CYBRD1, AHCY, GNPDA1, VIM, HSPB1, F5, FBP1, COMT, SERPINE2, LYZ, APOH, ANGPTL6, FAM168B, BLVRB, PLD3, CTSZ, SOGA1, GPX1, LAMB2, TUBB6, TIMP1, A2M, RRAS, IGSF8, NAAA, GAA, SGSH, PRADC1, ACTG1, PEBP1, EFHD1, TNFSF13, APP, PTGR1, CDC42BPB, ENDOD1, ATP6
GO:0070062	extracellular exosome	cellular component	61/287	1918/14310	0.00014000	0.0319	ENSAMEG00000000015, ENSAMEG00000000093, ENSAMEG00000000312, ENSAMEG00000000943, ENSAMEG00000001070, ENSAMEG00000001092, ENSAMEG00000001502, ENSAMEG00000002535, ENSAMEG00000002581, ENSAMEG00000002718, ENSAMEG00000002732, ENSAMEG00000003442, ENSAMEG00000003627, ENSAMEG00000003820, ENSAMEG00000004013, ENSAMEG00000004196, ENSAMEG00000004441, ENSAMEG00000004680, ENSAMEG00000006082, ENSAMEG00000006368, ENSAMEG00000007046, ENSAMEG00000007327, ENSAMEG00000007468, ENSAMEG00000007631, ENSAMEG00000008008, ENSAMEG00000008407, ENSAMEG00000008620, ENSAMEG00000009105, ENSAMEG00000010219, ENSAMEG00000010536, ENSAMEG00000010841, ENSAMEG00000011494, ENSAMEG00000011617, ENSAMEG00000011820, ENSAMEG00000011842, ENSAMEG00000012016, ENSAMEG00000012043, ENSAMEG00000012457, ENSAMEG00000012482, ENSAMEG00000012483, ENSAMEG00000012511, ENSAMEG00000012590, ENSAMEG00000012708, ENSAMEG00000012743, ENSAMEG00000012943, ENSAMEG00000013869, ENSAMEG00000013931, ENSAMEG00000013980, ENSAMEG00000014605, ENSAMEG00000015157, ENSAMEG00000015221, ENSAMEG00000015564, ENSAMEG00000016015, ENSAMEG00000016143, ENSAMEG00000016446, ENSAMEG00000016736, ENSAMEG00000017184, ENSAMEG00000017964, ENSAMEG00000018199, ENSAMEG00000019907, ENSAMEG00000023442	PDZK1IP1, NUDT14, GALNS, LPL, MINDY1, SLC1A4, ECM1, ANPEP, HNMT, SCPEP1, PLOD3, CRYZ, EPS8, CMBL, KCTD12, SLPI, GAS6, NANS, RHOQ, MRAS, TMEM8A, TRIP10, SMPDL3A, SLC7A8, OAF, SLC1A5, CYBRD1, AHCY, GNPDA1, VIM, HSPB1, FBP1, COMT, LYZ, APOH, ANGPTL6, FAM168B, BLVRB, PLD3, CTSZ, SOGA1, GPX1, LAMB2, TUBB6, TIMP1, A2M, RRAS, IGSF8, NAAA, GAA, SGSH, PRADC1, ACTG1, PEBP1, EFHD1, TNFSF13, APP, PTGR1, CDC42BPB, ENDOD1, ATP6
GO:0031012	extracellular matrix	cellular component	17/287	342/14310	0.00056000	0.1117	ENSAMEG00000001502, ENSAMEG00000003068, ENSAMEG00000004196, ENSAMEG00000007340, ENSAMEG00000007342, ENSAMEG00000009998, ENSAMEG00000010536, ENSAMEG00000010841, ENSAMEG00000011687, ENSAMEG00000011842, ENSAMEG00000012708, ENSAMEG00000012869, ENSAMEG00000012943, ENSAMEG00000013536, ENSAMEG00000015429, ENSAMEG00000016015, ENSAMEG00000018103	ECM1, MMP19, SLPI, ZP1, SDC2, EMILIN2, VIM, HSPB1, SERPINE2, APOH, LAMB2, CFP, TIMP1, ECM2, ELN, ACTG1, CCDC80
GO:0052547	regulation of peptidase activity	biological process	17/269	260/13500	0.00001800	0.127	ENSAMEG00000001502, ENSAMEG00000004196, ENSAMEG00000004441, ENSAMEG00000004774, ENSAMEG00000004836, ENSAMEG00000006630, ENSAMEG00000010076, ENSAMEG00000010287, ENSAMEG00000010991, ENSAMEG00000011687, ENSAMEG00000012590, ENSAMEG00000012943, ENSAMEG00000013869, ENSAMEG00000016947, ENSAMEG00000017062, ENSAMEG00000017184, ENSAMEG00000017990	ECM1, SLPI, GAS6, PPARG, TIMP4, NOD1, ENSAMEG00000010076, KIAA0141, HIP1, SERPINE2, GPX1, TIMP1, A2M, NLRP3, ARRB1, APP, BAD
GO:0052548	regulation of endopeptidase activity	biological process	16/269	234/13500	0.00001900	0.127	ENSAMEG00000004196, ENSAMEG00000004441, ENSAMEG00000004774, ENSAMEG00000004836, ENSAMEG00000006630, ENSAMEG00000010076, ENSAMEG00000010287, ENSAMEG00000010991, ENSAMEG00000011687, ENSAMEG00000012590, ENSAMEG00000012943, ENSAMEG00000013869, ENSAMEG00000016947, ENSAMEG00000017062, ENSAMEG00000017184, ENSAMEG00000017990	SLPI, GAS6, PPARG, TIMP4, NOD1, ENSAMEG00000010076, KIAA0141, HIP1, SERPINE2, GPX1, TIMP1, A2M, NLRP3, ARRB1, APP, BAD
GO:0019897	extrinsic component of plasma membrane	cellular component	6/287	63/14310	0.00161	0.2699	ENSAMEG00000006768, ENSAMEG00000008528, ENSAMEG00000011687, ENSAMEG00000013803, ENSAMEG00000016396, ENSAMEG00000017737	CYTH3, ENSAMEG00000008528, SERPINE2, FES, DLG4, GNG11
GO:0031234	extrinsic component of cytoplasmic side of plasma membrane	cellular component	5/287	44/14310	0.00179	0.2699	ENSAMEG00000006768, ENSAMEG00000008528, ENSAMEG00000013803, ENSAMEG00000016396, ENSAMEG00000017737	CYTH3, ENSAMEG00000008528, FES, DLG4, GNG11
GO:0098802	plasma membrane receptor complex	cellular component	8/287	112/14310	0.00186	0.2699	ENSAMEG00000003627, ENSAMEG00000004337, ENSAMEG00000006806, ENSAMEG00000012689, ENSAMEG00000014318, ENSAMEG00000015938, ENSAMEG00000016101, ENSAMEG00000016396	EPS8, ENSAMEG00000004337, ITGAX, PORCN, ACVR1, HFE, GABBR1, DLG4
GO:0005774	vacuolar membrane	cellular component	10/287	174/14310	0.00271	0.3487	ENSAMEG00000001230, ENSAMEG00000002535, ENSAMEG00000003625, ENSAMEG00000005486, ENSAMEG00000007046, ENSAMEG00000007997, ENSAMEG00000015157, ENSAMEG00000016765, ENSAMEG00000016795, ENSAMEG00000017739	TCIRG1, ANPEP, SBF2, SPPL2B, TMEM8A, CLCN7, GAA, CD68, TMEM175, SLC29A3
GO:0043235	receptor complex	cellular component	11/287	206/14310	0.003	0.3487	ENSAMEG00000003627, ENSAMEG00000004337, ENSAMEG00000006806, ENSAMEG00000010559, ENSAMEG00000012689, ENSAMEG00000014318, ENSAMEG00000015938, ENSAMEG00000016101, ENSAMEG00000016224, ENSAMEG00000016396, ENSAMEG00000017184	EPS8, ENSAMEG00000004337, ITGAX, ENSAMEG00000010559, PORCN, ACVR1, HFE, GABBR1, CD36, DLG4, APP
GO:0044437	vacuolar part	cellular component	10/287	180/14310	0.00345	0.3487	ENSAMEG00000001230, ENSAMEG00000002535, ENSAMEG00000003625, ENSAMEG00000005486, ENSAMEG00000007046, ENSAMEG00000007997, ENSAMEG00000015157, ENSAMEG00000016765, ENSAMEG00000016795, ENSAMEG00000017739	TCIRG1, ANPEP, SBF2, SPPL2B, TMEM8A, CLCN7, GAA, CD68, TMEM175, SLC29A3
GO:0031966	mitochondrial membrane	cellular component	15/287	338/14310	0.00348	0.3487	ENSAMEG00000001814, ENSAMEG00000002314, ENSAMEG00000002398, ENSAMEG00000006014, ENSAMEG00000008424, ENSAMEG00000011225, ENSAMEG00000011750, ENSAMEG00000011831, ENSAMEG00000016446, ENSAMEG00000017990, ENSAMEG00000023426, ENSAMEG00000023430, ENSAMEG00000023436, ENSAMEG00000023442, ENSAMEG00000023455	HADH, ENSAMEG00000002314, CHDH, RAB32, RNF144B, TMEM173, HAX1, IKBKE, EFHD1, BAD, ND1, ND2, COX1, ATP6, CYTB
GO:0098805	whole membrane	cellular component	22/287	592/14310	0.00409	0.3487	ENSAMEG00000001230, ENSAMEG00000002069, ENSAMEG00000002535, ENSAMEG00000003625, ENSAMEG00000005486, ENSAMEG00000006014, ENSAMEG00000006082, ENSAMEG00000007046, ENSAMEG00000007997, ENSAMEG00000010991, ENSAMEG00000011225, ENSAMEG00000011750, ENSAMEG00000012804, ENSAMEG00000015157, ENSAMEG00000016224, ENSAMEG00000016765, ENSAMEG00000016795, ENSAMEG00000017184, ENSAMEG00000017739, ENSAMEG00000017862, ENSAMEG00000017990, ENSAMEG00000020287	TCIRG1, KCNQ1, ANPEP, SBF2, SPPL2B, RAB32, RHOQ, TMEM8A, CLCN7, HIP1, TMEM173, HAX1, C3orf58, GAA, CD36, CD68, TMEM175, APP, SLC29A3, ABCA3, BAD, KCNE3
GO:0005764	lysosome	cellular component	13/287	279/14310	0.00424	0.3487	ENSAMEG00000001230, ENSAMEG00000002069, ENSAMEG00000002535, ENSAMEG00000005486, ENSAMEG00000007046, ENSAMEG00000007997, ENSAMEG00000012483, ENSAMEG00000014605, ENSAMEG00000015157, ENSAMEG00000015221, ENSAMEG00000016765, ENSAMEG00000016795, ENSAMEG00000017739	TCIRG1, KCNQ1, ANPEP, SPPL2B, TMEM8A, CLCN7, CTSZ, NAAA, GAA, SGSH, CD68, TMEM175, SLC29A3
GO:0005765	lysosomal membrane	cellular component	9/287	156/14310	0.00424	0.3487	ENSAMEG00000001230, ENSAMEG00000002535, ENSAMEG00000005486, ENSAMEG00000007046, ENSAMEG00000007997, ENSAMEG00000015157, ENSAMEG00000016765, ENSAMEG00000016795, ENSAMEG00000017739	TCIRG1, ANPEP, SPPL2B, TMEM8A, CLCN7, GAA, CD68, TMEM175, SLC29A3
GO:0098852	lytic vacuole membrane	cellular component	9/287	156/14310	0.00424	0.3487	ENSAMEG00000001230, ENSAMEG00000002535, ENSAMEG00000005486, ENSAMEG00000007046, ENSAMEG00000007997, ENSAMEG00000015157, ENSAMEG00000016765, ENSAMEG00000016795, ENSAMEG00000017739	TCIRG1, ANPEP, SPPL2B, TMEM8A, CLCN7, GAA, CD68, TMEM175, SLC29A3
GO:0000323	lytic vacuole	cellular component	13/287	280/14310	0.00437	0.3487	ENSAMEG00000001230, ENSAMEG00000002069, ENSAMEG00000002535, ENSAMEG00000005486, ENSAMEG00000007046, ENSAMEG00000007997, ENSAMEG00000012483, ENSAMEG00000014605, ENSAMEG00000015157, ENSAMEG00000015221, ENSAMEG00000016765, ENSAMEG00000016795, ENSAMEG00000017739	TCIRG1, KCNQ1, ANPEP, SPPL2B, TMEM8A, CLCN7, CTSZ, NAAA, GAA, SGSH, CD68, TMEM175, SLC29A3
GO:0004866	endopeptidase inhibitor activity	molecular function	9/273	102/13119	0.00027	0.3916	ENSAMEG00000004196, ENSAMEG00000004441, ENSAMEG00000004836, ENSAMEG00000010076, ENSAMEG00000011687, ENSAMEG00000012943, ENSAMEG00000013869, ENSAMEG00000017062, ENSAMEG00000017184	SLPI, GAS6, TIMP4, ENSAMEG00000010076, SERPINE2, TIMP1, A2M, ARRB1, APP
GO:0061135	endopeptidase regulator activity	molecular function	9/273	105/13119	0.00034	0.3916	ENSAMEG00000004196, ENSAMEG00000004441, ENSAMEG00000004836, ENSAMEG00000010076, ENSAMEG00000011687, ENSAMEG00000012943, ENSAMEG00000013869, ENSAMEG00000017062, ENSAMEG00000017184	SLPI, GAS6, TIMP4, ENSAMEG00000010076, SERPINE2, TIMP1, A2M, ARRB1, APP
GO:0008484	sulfuric ester hydrolase activity	molecular function	4/273	17/13119	0.00035	0.3916	ENSAMEG00000000312, ENSAMEG00000013098, ENSAMEG00000014912, ENSAMEG00000015221	GALNS, ENSAMEG00000013098, ENSAMEG00000014912, SGSH
GO:0061134	peptidase regulator activity	molecular function	10/273	132/13119	0.00043	0.3916	ENSAMEG00000004196, ENSAMEG00000004441, ENSAMEG00000004836, ENSAMEG00000010076, ENSAMEG00000011687, ENSAMEG00000012943, ENSAMEG00000013869, ENSAMEG00000017062, ENSAMEG00000017184, ENSAMEG00000017990	SLPI, GAS6, TIMP4, ENSAMEG00000010076, SERPINE2, TIMP1, A2M, ARRB1, APP, BAD
GO:0045275	respiratory chain complex III	cellular component	2/287	6/14310	0.0057	0.4233	ENSAMEG00000023436, ENSAMEG00000023455	COX1, CYTB
GO:0008328	ionotropic glutamate receptor complex	cellular component	3/287	19/14310	0.0061	0.4233	ENSAMEG00000003627, ENSAMEG00000012689, ENSAMEG00000016396	EPS8, PORCN, DLG4
GO:0098878	neurotransmitter receptor complex	cellular component	3/287	19/14310	0.0061	0.4233	ENSAMEG00000003627, ENSAMEG00000012689, ENSAMEG00000016396	EPS8, PORCN, DLG4
GO:0098552	side of membrane	cellular component	10/287	198/14310	0.00671	0.425	ENSAMEG00000005486, ENSAMEG00000006768, ENSAMEG00000008528, ENSAMEG00000011687, ENSAMEG00000013414, ENSAMEG00000013803, ENSAMEG00000015938, ENSAMEG00000016224, ENSAMEG00000016396, ENSAMEG00000017737	SPPL2B, CYTH3, ENSAMEG00000008528, SERPINE2, CD163, FES, HFE, CD36, DLG4, GNG11
GO:0005773	vacuole	cellular component	14/287	329/14310	0.00673	0.425	ENSAMEG00000001230, ENSAMEG00000002069, ENSAMEG00000002535, ENSAMEG00000003625, ENSAMEG00000005486, ENSAMEG00000007046, ENSAMEG00000007997, ENSAMEG00000012483, ENSAMEG00000014605, ENSAMEG00000015157, ENSAMEG00000015221, ENSAMEG00000016765, ENSAMEG00000016795, ENSAMEG00000017739	TCIRG1, KCNQ1, ANPEP, SBF2, SPPL2B, TMEM8A, CLCN7, CTSZ, NAAA, GAA, SGSH, CD68, TMEM175, SLC29A3
GO:0005740	mitochondrial envelope	cellular component	15/287	365/14310	0.00699	0.425	ENSAMEG00000001814, ENSAMEG00000002314, ENSAMEG00000002398, ENSAMEG00000006014, ENSAMEG00000008424, ENSAMEG00000011225, ENSAMEG00000011750, ENSAMEG00000011831, ENSAMEG00000016446, ENSAMEG00000017990, ENSAMEG00000023426, ENSAMEG00000023430, ENSAMEG00000023436, ENSAMEG00000023442, ENSAMEG00000023455	HADH, ENSAMEG00000002314, CHDH, RAB32, RNF144B, TMEM173, HAX1, IKBKE, EFHD1, BAD, ND1, ND2, COX1, ATP6, CYTB
GO:0098562	cytoplasmic side of membrane	cellular component	6/287	85/14310	0.00719	0.425	ENSAMEG00000005486, ENSAMEG00000006768, ENSAMEG00000008528, ENSAMEG00000013803, ENSAMEG00000016396, ENSAMEG00000017737	SPPL2B, CYTH3, ENSAMEG00000008528, FES, DLG4, GNG11
GO:0005614	interstitial matrix	cellular component	2/287	7/14310	0.00788	0.4492	ENSAMEG00000013536, ENSAMEG00000018103	ECM2, CCDC80
GO:0030414	peptidase inhibitor activity	molecular function	9/273	114/13119	0.00062	0.4517	ENSAMEG00000004196, ENSAMEG00000004441, ENSAMEG00000004836, ENSAMEG00000010076, ENSAMEG00000011687, ENSAMEG00000012943, ENSAMEG00000013869, ENSAMEG00000017062, ENSAMEG00000017184	SLPI, GAS6, TIMP4, ENSAMEG00000010076, SERPINE2, TIMP1, A2M, ARRB1, APP
GO:0005912	adherens junction	cellular component	14/287	337/14310	0.00824	0.4535	ENSAMEG00000003894, ENSAMEG00000006768, ENSAMEG00000010536, ENSAMEG00000010841, ENSAMEG00000013263, ENSAMEG00000013297, ENSAMEG00000013803, ENSAMEG00000013931, ENSAMEG00000014689, ENSAMEG00000015431, ENSAMEG00000015837, ENSAMEG00000016015, ENSAMEG00000016087, ENSAMEG00000019485	MRC2, CYTH3, VIM, HSPB1, REXO2, FHL2, FES, RRAS, TNS3, LIMK1, STXBP6, ACTG1, PDLIM1, CDC42EP4
GO:0031410	cytoplasmic vesicle	cellular component	25/287	755/14310	0.00965	0.4966	ENSAMEG00000001276, ENSAMEG00000002069, ENSAMEG00000005486, ENSAMEG00000006014, ENSAMEG00000008497, ENSAMEG00000008800, ENSAMEG00000009193, ENSAMEG00000010113, ENSAMEG00000010991, ENSAMEG00000011403, ENSAMEG00000011509, ENSAMEG00000011687, ENSAMEG00000011750, ENSAMEG00000012016, ENSAMEG00000012483, ENSAMEG00000012804, ENSAMEG00000013803, ENSAMEG00000014724, ENSAMEG00000015326, ENSAMEG00000015938, ENSAMEG00000016396, ENSAMEG00000016795, ENSAMEG00000017062, ENSAMEG00000017184, ENSAMEG00000017862	ZNRF1, KCNQ1, SPPL2B, RAB32, STEAP3, DGKH, DMXL2, SLA2, HIP1, F5, SEPT5, SERPINE2, HAX1, ANGPTL6, CTSZ, C3orf58, FES, ITSN1, MICALL2, HFE, DLG4, TMEM175, ARRB1, APP, ABCA3
GO:0097708	intracellular vesicle	cellular component	25/287	756/14310	0.0098	0.4966	ENSAMEG00000001276, ENSAMEG00000002069, ENSAMEG00000005486, ENSAMEG00000006014, ENSAMEG00000008497, ENSAMEG00000008800, ENSAMEG00000009193, ENSAMEG00000010113, ENSAMEG00000010991, ENSAMEG00000011403, ENSAMEG00000011509, ENSAMEG00000011687, ENSAMEG00000011750, ENSAMEG00000012016, ENSAMEG00000012483, ENSAMEG00000012804, ENSAMEG00000013803, ENSAMEG00000014724, ENSAMEG00000015326, ENSAMEG00000015938, ENSAMEG00000016396, ENSAMEG00000016795, ENSAMEG00000017062, ENSAMEG00000017184, ENSAMEG00000017862	ZNRF1, KCNQ1, SPPL2B, RAB32, STEAP3, DGKH, DMXL2, SLA2, HIP1, F5, SEPT5, SERPINE2, HAX1, ANGPTL6, CTSZ, C3orf58, FES, ITSN1, MICALL2, HFE, DLG4, TMEM175, ARRB1, APP, ABCA3
GO:0070161	anchoring junction	cellular component	14/287	345/14310	0.01	0.4966	ENSAMEG00000003894, ENSAMEG00000006768, ENSAMEG00000010536, ENSAMEG00000010841, ENSAMEG00000013263, ENSAMEG00000013297, ENSAMEG00000013803, ENSAMEG00000013931, ENSAMEG00000014689, ENSAMEG00000015431, ENSAMEG00000015837, ENSAMEG00000016015, ENSAMEG00000016087, ENSAMEG00000019485	MRC2, CYTH3, VIM, HSPB1, REXO2, FHL2, FES, RRAS, TNS3, LIMK1, STXBP6, ACTG1, PDLIM1, CDC42EP4
GO:0042627	chylomicron	cellular component	2/287	8/14310	0.01036	0.4966	ENSAMEG00000000943, ENSAMEG00000011842	LPL, APOH
GO:0042641	actomyosin	cellular component	4/287	43/14310	0.01058	0.4966	ENSAMEG00000015326, ENSAMEG00000017327, ENSAMEG00000017670, ENSAMEG00000018199	MICALL2, FHOD1, PDLIM4, CDC42BPB
GO:0030027	lamellipodium	cellular component	6/287	96/14310	0.01272	0.5715	ENSAMEG00000004387, ENSAMEG00000007278, ENSAMEG00000011750, ENSAMEG00000014724, ENSAMEG00000015431, ENSAMEG00000017670	PLXND1, ARHGAP31, HAX1, ITSN1, LIMK1, PDLIM4
GO:0032281	AMPA glutamate receptor complex	cellular component	2/287	9/14310	0.01315	0.5715	ENSAMEG00000012689, ENSAMEG00000016396	PORCN, DLG4
GO:0031252	cell leading edge	cellular component	9/287	187/14310	0.01325	0.5715	ENSAMEG00000003627, ENSAMEG00000004387, ENSAMEG00000006768, ENSAMEG00000007278, ENSAMEG00000010536, ENSAMEG00000011750, ENSAMEG00000014724, ENSAMEG00000015431, ENSAMEG00000017670	EPS8, PLXND1, CYTH3, ARHGAP31, VIM, HAX1, ITSN1, LIMK1, PDLIM4
GO:0051336	regulation of hydrolase activity	biological process	28/269	690/13500	0.00028	0.5728	ENSAMEG00000001502, ENSAMEG00000003625, ENSAMEG00000004196, ENSAMEG00000004441, ENSAMEG00000004774, ENSAMEG00000004836, ENSAMEG00000005271, ENSAMEG00000005566, ENSAMEG00000006453, ENSAMEG00000006630, ENSAMEG00000007278, ENSAMEG00000008477, ENSAMEG00000010076, ENSAMEG00000010287, ENSAMEG00000010991, ENSAMEG00000011687, ENSAMEG00000011842, ENSAMEG00000012590, ENSAMEG00000012943, ENSAMEG00000013869, ENSAMEG00000014678, ENSAMEG00000015030, ENSAMEG00000015604, ENSAMEG00000016947, ENSAMEG00000017062, ENSAMEG00000017184, ENSAMEG00000017990, ENSAMEG00000019280	ECM1, SBF2, SLPI, GAS6, PPARG, TIMP4, ENSAMEG00000005271, ADAP2, ARHGAP21, NOD1, ARHGAP31, HSCB, ENSAMEG00000010076, KIAA0141, HIP1, SERPINE2, APOH, GPX1, TIMP1, A2M, PPP1R15A, ENSAMEG00000015030, NUAK1, NLRP3, ARRB1, APP, BAD, CDC42EP2
GO:0010466	negative regulation of peptidase activity	biological process	11/269	157/13500	0.00031	0.5728	ENSAMEG00000001502, ENSAMEG00000004196, ENSAMEG00000004441, ENSAMEG00000004836, ENSAMEG00000010076, ENSAMEG00000011687, ENSAMEG00000012590, ENSAMEG00000012943, ENSAMEG00000013869, ENSAMEG00000017062, ENSAMEG00000017184	ECM1, SLPI, GAS6, TIMP4, ENSAMEG00000010076, SERPINE2, GPX1, TIMP1, A2M, ARRB1, APP
GO:0002376	immune system process	biological process	43/269	1258/13500	0.00031	0.5728	ENSAMEG00000000272, ENSAMEG00000001502, ENSAMEG00000003627, ENSAMEG00000003641, ENSAMEG00000004196, ENSAMEG00000004441, ENSAMEG00000004516, ENSAMEG00000004663, ENSAMEG00000004774, ENSAMEG00000004811, ENSAMEG00000005486, ENSAMEG00000006014, ENSAMEG00000006630, ENSAMEG00000006928, ENSAMEG00000007284, ENSAMEG00000007512, ENSAMEG00000008499, ENSAMEG00000010113, ENSAMEG00000011225, ENSAMEG00000011750, ENSAMEG00000011831, ENSAMEG00000012283, ENSAMEG00000012418, ENSAMEG00000012590, ENSAMEG00000012594, ENSAMEG00000013803, ENSAMEG00000013869, ENSAMEG00000013931, ENSAMEG00000014335, ENSAMEG00000014490, ENSAMEG00000014906, ENSAMEG00000014908, ENSAMEG00000015180, ENSAMEG00000015938, ENSAMEG00000016224, ENSAMEG00000016729, ENSAMEG00000016736, ENSAMEG00000016820, ENSAMEG00000017184, ENSAMEG00000017745, ENSAMEG00000017990, ENSAMEG00000018841, ENSAMEG00000019893	CBFA2T3, ECM1, EPS8, CARD9, SLPI, GAS6, CD1D, TRIM14, PPARG, POU2F2, SPPL2B, RAB32, NOD1, DUSP10, CYBB, HSD3B7, ZFP36, SLA2, TMEM173, HAX1, IKBKE, SDHAF4, ERCC1, GPX1, LYL1, FES, A2M, RRAS, ENSAMEG00000014335, IL5RA, CCL14, ENSAMEG00000014908, GATA2, HFE, CD36, TNFSF12, TNFSF13, IL25, APP, BATF2, BAD, CEBPG, CYSLTR1
GO:0055095	lipoprotein particle mediated signaling	biological process	2/269	2/13500	0.0004	0.5728	ENSAMEG00000000943, ENSAMEG00000016224	LPL, CD36
GO:0055096	low-density lipoprotein particle mediated signaling	biological process	2/269	2/13500	0.0004	0.5728	ENSAMEG00000000943, ENSAMEG00000016224	LPL, CD36
GO:0010885	regulation of cholesterol storage	biological process	3/269	8/13500	0.00041	0.5728	ENSAMEG00000000943, ENSAMEG00000004774, ENSAMEG00000016224	LPL, PPARG, CD36
GO:0045861	negative regulation of proteolysis	biological process	13/269	218/13500	0.00043	0.5728	ENSAMEG00000001502, ENSAMEG00000004196, ENSAMEG00000004441, ENSAMEG00000004836, ENSAMEG00000010076, ENSAMEG00000011687, ENSAMEG00000012483, ENSAMEG00000012590, ENSAMEG00000012943, ENSAMEG00000013869, ENSAMEG00000015938, ENSAMEG00000017062, ENSAMEG00000017184	ECM1, SLPI, GAS6, TIMP4, ENSAMEG00000010076, SERPINE2, CTSZ, GPX1, TIMP1, A2M, HFE, ARRB1, APP
GO:0030162	regulation of proteolysis	biological process	20/269	436/13500	0.00047	0.5728	ENSAMEG00000000272, ENSAMEG00000001502, ENSAMEG00000004196, ENSAMEG00000004441, ENSAMEG00000004774, ENSAMEG00000004836, ENSAMEG00000006630, ENSAMEG00000010076, ENSAMEG00000010287, ENSAMEG00000010991, ENSAMEG00000011687, ENSAMEG00000012483, ENSAMEG00000012590, ENSAMEG00000012943, ENSAMEG00000013869, ENSAMEG00000015938, ENSAMEG00000016947, ENSAMEG00000017062, ENSAMEG00000017184, ENSAMEG00000017990	CBFA2T3, ECM1, SLPI, GAS6, PPARG, TIMP4, NOD1, ENSAMEG00000010076, KIAA0141, HIP1, SERPINE2, CTSZ, GPX1, TIMP1, A2M, HFE, NLRP3, ARRB1, APP, BAD
GO:0006955	immune response	biological process	27/269	686/13500	0.00058	0.5728	ENSAMEG00000001502, ENSAMEG00000003641, ENSAMEG00000004196, ENSAMEG00000004441, ENSAMEG00000004663, ENSAMEG00000004774, ENSAMEG00000004811, ENSAMEG00000005486, ENSAMEG00000007284, ENSAMEG00000011225, ENSAMEG00000011831, ENSAMEG00000012283, ENSAMEG00000012418, ENSAMEG00000012590, ENSAMEG00000013803, ENSAMEG00000013869, ENSAMEG00000014335, ENSAMEG00000014490, ENSAMEG00000014906, ENSAMEG00000014908, ENSAMEG00000015938, ENSAMEG00000016224, ENSAMEG00000016729, ENSAMEG00000016736, ENSAMEG00000017184, ENSAMEG00000018841, ENSAMEG00000019893	ECM1, CARD9, SLPI, GAS6, TRIM14, PPARG, POU2F2, SPPL2B, CYBB, TMEM173, IKBKE, SDHAF4, ERCC1, GPX1, FES, A2M, ENSAMEG00000014335, IL5RA, CCL14, ENSAMEG00000014908, HFE, CD36, TNFSF12, TNFSF13, APP, CEBPG, CYSLTR1
GO:0030851	granulocyte differentiation	biological process	4/269	20/13500	0.00058	0.5728	ENSAMEG00000000272, ENSAMEG00000011750, ENSAMEG00000015180, ENSAMEG00000016820	CBFA2T3, HAX1, GATA2, IL25
GO:0010951	negative regulation of endopeptidase activity	biological process	10/269	143/13500	0.00058	0.5728	ENSAMEG00000004196, ENSAMEG00000004441, ENSAMEG00000004836, ENSAMEG00000010076, ENSAMEG00000011687, ENSAMEG00000012590, ENSAMEG00000012943, ENSAMEG00000013869, ENSAMEG00000017062, ENSAMEG00000017184	SLPI, GAS6, TIMP4, ENSAMEG00000010076, SERPINE2, GPX1, TIMP1, A2M, ARRB1, APP
GO:0010878	cholesterol storage	biological process	3/269	9/13500	0.0006	0.5728	ENSAMEG00000000943, ENSAMEG00000004774, ENSAMEG00000016224	LPL, PPARG, CD36
GO:0098797	plasma membrane protein complex	cellular component	14/287	360/14310	0.01408	0.5914	ENSAMEG00000002069, ENSAMEG00000003627, ENSAMEG00000004337, ENSAMEG00000006806, ENSAMEG00000007284, ENSAMEG00000008528, ENSAMEG00000010991, ENSAMEG00000012689, ENSAMEG00000014318, ENSAMEG00000015938, ENSAMEG00000016101, ENSAMEG00000016396, ENSAMEG00000017737, ENSAMEG00000020287	KCNQ1, EPS8, ENSAMEG00000004337, ITGAX, CYBB, ENSAMEG00000008528, HIP1, PORCN, ACVR1, HFE, GABBR1, DLG4, GNG11, KCNE3
GO:1990204	oxidoreductase complex	cellular component	5/287	72/14310	0.01471	0.602	ENSAMEG00000007284, ENSAMEG00000023426, ENSAMEG00000023430, ENSAMEG00000023436, ENSAMEG00000023455	CYBB, ND1, ND2, COX1, CYTB
GO:0098796	membrane protein complex	cellular component	24/287	749/14310	0.01602	0.6392	ENSAMEG00000001230, ENSAMEG00000002069, ENSAMEG00000003627, ENSAMEG00000004337, ENSAMEG00000006083, ENSAMEG00000006806, ENSAMEG00000007284, ENSAMEG00000008528, ENSAMEG00000009860, ENSAMEG00000010991, ENSAMEG00000012689, ENSAMEG00000012804, ENSAMEG00000014318, ENSAMEG00000015938, ENSAMEG00000016101, ENSAMEG00000016396, ENSAMEG00000017737, ENSAMEG00000020108, ENSAMEG00000020287, ENSAMEG00000023426, ENSAMEG00000023430, ENSAMEG00000023436, ENSAMEG00000023442, ENSAMEG00000023455	TCIRG1, KCNQ1, EPS8, ENSAMEG00000004337, AP2A2, ITGAX, CYBB, ENSAMEG00000008528, ORMDL3, HIP1, PORCN, C3orf58, ACVR1, HFE, GABBR1, DLG4, GNG11, OST4, KCNE3, ND1, ND2, COX1, ATP6, CYTB
GO:0009898	cytoplasmic side of plasma membrane	cellular component	5/287	75/14310	0.01729	0.6534	ENSAMEG00000006768, ENSAMEG00000008528, ENSAMEG00000013803, ENSAMEG00000016396, ENSAMEG00000017737	CYTH3, ENSAMEG00000008528, FES, DLG4, GNG11
GO:0044304	main axon	cellular component	3/287	28/14310	0.01806	0.6534	ENSAMEG00000012095, ENSAMEG00000016396, ENSAMEG00000016637	NAV1, DLG4, DAGLA
GO:0016020	membrane	cellular component	165/287	7329/14310	0.0182	0.6534	ENSAMEG00000000015, ENSAMEG00000000095, ENSAMEG00000000381, ENSAMEG00000000590, ENSAMEG00000000602, ENSAMEG00000000751, ENSAMEG00000000943, ENSAMEG00000001092, ENSAMEG00000001230, ENSAMEG00000001267, ENSAMEG00000001276, ENSAMEG00000001318, ENSAMEG00000001514, ENSAMEG00000001784, ENSAMEG00000001814, ENSAMEG00000001879, ENSAMEG00000002069, ENSAMEG00000002314, ENSAMEG00000002398, ENSAMEG00000002415, ENSAMEG00000002508, ENSAMEG00000002535, ENSAMEG00000003020, ENSAMEG00000003086, ENSAMEG00000003089, ENSAMEG00000003400, ENSAMEG00000003591, ENSAMEG00000003625, ENSAMEG00000003627, ENSAMEG00000003733, ENSAMEG00000003778, ENSAMEG00000003894, ENSAMEG00000003970, ENSAMEG00000004337, ENSAMEG00000004387, ENSAMEG00000004469, ENSAMEG00000004481, ENSAMEG00000004516, ENSAMEG00000004600, ENSAMEG00000005113, ENSAMEG00000005412, ENSAMEG00000005486, ENSAMEG00000005566, ENSAMEG00000005794, ENSAMEG00000005927, ENSAMEG00000006014, ENSAMEG00000006082, ENSAMEG00000006083, ENSAMEG00000006288, ENSAMEG00000006368, ENSAMEG00000006453, ENSAMEG00000006630, ENSAMEG00000006677, ENSAMEG00000006768, ENSAMEG00000006806, ENSAMEG00000006896, ENSAMEG00000007046, ENSAMEG00000007223, ENSAMEG00000007284, ENSAMEG00000007302, ENSAMEG00000007340, ENSAMEG00000007342, ENSAMEG00000007512, ENSAMEG00000007631, ENSAMEG00000007997, ENSAMEG00000008407, ENSAMEG00000008416, ENSAMEG00000008424, ENSAMEG00000008433, ENSAMEG00000008497, ENSAMEG00000008528, ENSAMEG00000008575, ENSAMEG00000008620, ENSAMEG00000009198, ENSAMEG00000009367, ENSAMEG00000009654, ENSAMEG00000009860, ENSAMEG00000010113, ENSAMEG00000010536, ENSAMEG00000010841, ENSAMEG00000010857, ENSAMEG00000010898, ENSAMEG00000010991, ENSAMEG00000011225, ENSAMEG00000011403, ENSAMEG00000011408, ENSAMEG00000011455, ENSAMEG00000011509, ENSAMEG00000011564, ENSAMEG00000011617, ENSAMEG00000011687, ENSAMEG00000011750, ENSAMEG00000011831, ENSAMEG00000011849, ENSAMEG00000011977, ENSAMEG00000012457, ENSAMEG00000012482, ENSAMEG00000012576, ENSAMEG00000012689, ENSAMEG00000012804, ENSAMEG00000013414, ENSAMEG00000013445, ENSAMEG00000013447, ENSAMEG00000013560, ENSAMEG00000013803, ENSAMEG00000013851, ENSAMEG00000013931, ENSAMEG00000013980, ENSAMEG00000014079, ENSAMEG00000014279, ENSAMEG00000014318, ENSAMEG00000014354, ENSAMEG00000014490, ENSAMEG00000014678, ENSAMEG00000014705, ENSAMEG00000014724, ENSAMEG00000015157, ENSAMEG00000015252, ENSAMEG00000015326, ENSAMEG00000015431, ENSAMEG00000015837, ENSAMEG00000015938, ENSAMEG00000015964, ENSAMEG00000016015, ENSAMEG00000016101, ENSAMEG00000016224, ENSAMEG00000016396, ENSAMEG00000016446, ENSAMEG00000016637, ENSAMEG00000016729, ENSAMEG00000016736, ENSAMEG00000016739, ENSAMEG00000016765, ENSAMEG00000016795, ENSAMEG00000016820, ENSAMEG00000017107, ENSAMEG00000017184, ENSAMEG00000017327, ENSAMEG00000017329, ENSAMEG00000017737, ENSAMEG00000017739, ENSAMEG00000017834, ENSAMEG00000017862, ENSAMEG00000017990, ENSAMEG00000018122, ENSAMEG00000018339, ENSAMEG00000018345, ENSAMEG00000018637, ENSAMEG00000018844, ENSAMEG00000019280, ENSAMEG00000019485, ENSAMEG00000019659, ENSAMEG00000019719, ENSAMEG00000019893, ENSAMEG00000019905, ENSAMEG00000019907, ENSAMEG00000020108, ENSAMEG00000020206, ENSAMEG00000020281, ENSAMEG00000020287, ENSAMEG00000023426, ENSAMEG00000023430, ENSAMEG00000023436, ENSAMEG00000023442, ENSAMEG00000023455	PDZK1IP1, MARVELD1, ENSAMEG00000000381, IGFLR1, EPB41L3, TP53I11, LPL, SLC1A4, TCIRG1, ST7, ZNRF1, ENSAMEG00000001318, NAGPA, TTC7B, HADH, SEMA6B, KCNQ1, ENSAMEG00000002314, CHDH, IL17RB, TMEM45B, ANPEP, HACD4, NRP2, PNKD, ENSAMEG00000003400, TMEM26, SBF2, EPS8, INPP5E, IL10RA, MRC2, ENSAMEG00000003970, ENSAMEG00000004337, PLXND1, ENSAMEG00000004469, ENSAMEG00000004481, CD1D, NDST1, SCD5, ADORA3, SPPL2B, ADAP2, LHFPL2, ATP10A, RAB32, RHOQ, AP2A2, IFI27L2, MRAS, ARHGAP21, NOD1, NINJ1, CYTH3, ITGAX, IL11RA, TMEM8A, SLC7A7, CYBB, GPR108, ZP1, SDC2, HSD3B7, SLC7A8, CLCN7, SLC1A5, KREMEN1, RNF144B, ENSAMEG00000008433, STEAP3, ENSAMEG00000008528, STAB1, CYBRD1, ENSAMEG00000009198, XXYLT1, ENSAMEG00000009654, ORMDL3, SLA2, VIM, HSPB1, ADCY9, VANGL1, HIP1, TMEM173, F5, HHIPL1, PDE4A, SEPT5, SLC2A6, COMT, SERPINE2, HAX1, IKBKE, SLC29A1, ENSAMEG00000011977, BLVRB, PLD3, DNAJA4, PORCN, C3orf58, CD163, ENSAMEG00000013445, ENSAMEG00000013447, ENSAMEG00000013560, FES, TSPAN5, RRAS, IGSF8, TMEM53, SIGLECL1, ACVR1, ENSAMEG00000014354, IL5RA, PPP1R15A, ENSAMEG00000014705, ITSN1, GAA, RILPL1, MICALL2, LIMK1, STXBP6, HFE, ADCY4, ACTG1, GABBR1, CD36, DLG4, EFHD1, DAGLA, TNFSF12, TNFSF13, MFSD7, CD68, TMEM175, IL25, RIN1, APP, FHOD1, SLC17A9, GNG11, SLC29A3, BRICD5, ABCA3, BAD, ENSAMEG00000018122, FLVCR2, TTLL5, LPAR6, TMEM37, CDC42EP2, CDC42EP4, S1PR2, B3GNT9, CYSLTR1, OR2K2, ENDOD1, OST4, PAQR7, B3GALT2, KCNE3, ND1, ND2, COX1, ATP6, CYTB
GO:0005608	laminin-3 complex	cellular component	1/287	1/14310	0.02006	0.6534	ENSAMEG00000012708	LAMB2
GO:0038039	G-protein coupled receptor heterodimeric complex	cellular component	1/287	1/14310	0.02006	0.6534	ENSAMEG00000016101	GABBR1
GO:0043196	varicosity	cellular component	1/287	1/14310	0.02006	0.6534	ENSAMEG00000016637	DAGLA
GO:0097232	lamellar body membrane	cellular component	1/287	1/14310	0.02006	0.6534	ENSAMEG00000017862	ABCA3
GO:0097233	alveolar lamellar body membrane	cellular component	1/287	1/14310	0.02006	0.6534	ENSAMEG00000017862	ABCA3
GO:0098839	postsynaptic density membrane	cellular component	1/287	1/14310	0.02006	0.6534	ENSAMEG00000016396	DLG4
GO:0098803	respiratory chain complex	cellular component	4/287	54/14310	0.02287	0.73	ENSAMEG00000023426, ENSAMEG00000023430, ENSAMEG00000023436, ENSAMEG00000023455	ND1, ND2, COX1, CYTB
GO:0043281	regulation of cysteine-type endopeptidase activity involved in apoptotic process	biological process	9/269	125/13500	0.00088	0.7841	ENSAMEG00000004441, ENSAMEG00000004774, ENSAMEG00000006630, ENSAMEG00000010287, ENSAMEG00000010991, ENSAMEG00000012590, ENSAMEG00000016947, ENSAMEG00000017062, ENSAMEG00000017990	GAS6, PPARG, NOD1, KIAA0141, HIP1, GPX1, NLRP3, ARRB1, BAD
GO:0005925	focal adhesion	cellular component	11/287	281/14310	0.02663	0.8278	ENSAMEG00000003894, ENSAMEG00000010536, ENSAMEG00000010841, ENSAMEG00000013263, ENSAMEG00000013297, ENSAMEG00000013803, ENSAMEG00000013931, ENSAMEG00000014689, ENSAMEG00000015431, ENSAMEG00000016015, ENSAMEG00000016087	MRC2, VIM, HSPB1, REXO2, FHL2, FES, RRAS, TNS3, LIMK1, ACTG1, PDLIM1
GO:0070069	cytochrome complex	cellular component	2/287	13/14310	0.02702	0.8278	ENSAMEG00000023436, ENSAMEG00000023455	COX1, CYTB
GO:0019898	extrinsic component of membrane	cellular component	6/287	115/14310	0.02828	0.8278	ENSAMEG00000006768, ENSAMEG00000008528, ENSAMEG00000011687, ENSAMEG00000013803, ENSAMEG00000016396, ENSAMEG00000017737	CYTH3, ENSAMEG00000008528, SERPINE2, FES, DLG4, GNG11
GO:0005924	cell-substrate adherens junction	cellular component	11/287	284/14310	0.02848	0.8278	ENSAMEG00000003894, ENSAMEG00000010536, ENSAMEG00000010841, ENSAMEG00000013263, ENSAMEG00000013297, ENSAMEG00000013803, ENSAMEG00000013931, ENSAMEG00000014689, ENSAMEG00000015431, ENSAMEG00000016015, ENSAMEG00000016087	MRC2, VIM, HSPB1, REXO2, FHL2, FES, RRAS, TNS3, LIMK1, ACTG1, PDLIM1
GO:0120025	plasma membrane bounded cell projection	cellular component	24/287	793/14310	0.02923	0.8278	ENSAMEG00000003086, ENSAMEG00000003627, ENSAMEG00000003733, ENSAMEG00000004387, ENSAMEG00000006768, ENSAMEG00000007278, ENSAMEG00000008620, ENSAMEG00000010536, ENSAMEG00000010841, ENSAMEG00000011509, ENSAMEG00000011750, ENSAMEG00000012095, ENSAMEG00000014724, ENSAMEG00000015252, ENSAMEG00000015431, ENSAMEG00000015964, ENSAMEG00000016224, ENSAMEG00000016396, ENSAMEG00000016637, ENSAMEG00000017062, ENSAMEG00000017184, ENSAMEG00000017670, ENSAMEG00000018345, ENSAMEG00000020287	NRP2, EPS8, INPP5E, PLXND1, CYTH3, ARHGAP31, CYBRD1, VIM, HSPB1, SEPT5, HAX1, NAV1, ITSN1, RILPL1, LIMK1, ADCY4, CD36, DLG4, DAGLA, ARRB1, APP, PDLIM4, TTLL5, KCNE3
GO:0031090	organelle membrane	cellular component	31/287	1086/14310	0.02933	0.8278	ENSAMEG00000001230, ENSAMEG00000001814, ENSAMEG00000002314, ENSAMEG00000002398, ENSAMEG00000002535, ENSAMEG00000003625, ENSAMEG00000003733, ENSAMEG00000005486, ENSAMEG00000006014, ENSAMEG00000007046, ENSAMEG00000007997, ENSAMEG00000008424, ENSAMEG00000010991, ENSAMEG00000011225, ENSAMEG00000011750, ENSAMEG00000011831, ENSAMEG00000012804, ENSAMEG00000015157, ENSAMEG00000016446, ENSAMEG00000016765, ENSAMEG00000016795, ENSAMEG00000017739, ENSAMEG00000017862, ENSAMEG00000017990, ENSAMEG00000019719, ENSAMEG00000020281, ENSAMEG00000023426, ENSAMEG00000023430, ENSAMEG00000023436, ENSAMEG00000023442, ENSAMEG00000023455	TCIRG1, HADH, ENSAMEG00000002314, CHDH, ANPEP, SBF2, INPP5E, SPPL2B, RAB32, TMEM8A, CLCN7, RNF144B, HIP1, TMEM173, HAX1, IKBKE, C3orf58, GAA, EFHD1, CD68, TMEM175, SLC29A3, ABCA3, BAD, B3GNT9, B3GALT2, ND1, ND2, COX1, ATP6, CYTB
GO:0030055	cell-substrate junction	cellular component	11/287	287/14310	0.03042	0.8278	ENSAMEG00000003894, ENSAMEG00000010536, ENSAMEG00000010841, ENSAMEG00000013263, ENSAMEG00000013297, ENSAMEG00000013803, ENSAMEG00000013931, ENSAMEG00000014689, ENSAMEG00000015431, ENSAMEG00000016015, ENSAMEG00000016087	MRC2, VIM, HSPB1, REXO2, FHL2, FES, RRAS, TNS3, LIMK1, ACTG1, PDLIM1
GO:0031091	platelet alpha granule	cellular component	2/287	14/14310	0.03112	0.8278	ENSAMEG00000011403, ENSAMEG00000011687	F5, SERPINE2
GO:0034361	very-low-density lipoprotein particle	cellular component	2/287	14/14310	0.03112	0.8278	ENSAMEG00000000943, ENSAMEG00000011842	LPL, APOH
GO:0034385	triglyceride-rich plasma lipoprotein particle	cellular component	2/287	14/14310	0.03112	0.8278	ENSAMEG00000000943, ENSAMEG00000011842	LPL, APOH
GO:0061077	chaperone-mediated protein folding	biological process	4/269	23/13500	0.00101	0.8293	ENSAMEG00000000640, ENSAMEG00000002444, ENSAMEG00000010841, ENSAMEG00000014637	HSPB6, CRTAP, HSPB1, UNC45B
GO:0030222	eosinophil differentiation	biological process	2/269	3/13500	0.00117	0.8293	ENSAMEG00000015180, ENSAMEG00000016820	GATA2, IL25
GO:0031532	actin cytoskeleton reorganization	biological process	6/269	60/13500	0.0012	0.8293	ENSAMEG00000003627, ENSAMEG00000011750, ENSAMEG00000013803, ENSAMEG00000015326, ENSAMEG00000017670, ENSAMEG00000019659	EPS8, HAX1, FES, MICALL2, PDLIM4, S1PR2
GO:0030029	actin filament-based process	biological process	19/269	442/13500	0.0014	0.8293	ENSAMEG00000000602, ENSAMEG00000002069, ENSAMEG00000003627, ENSAMEG00000005610, ENSAMEG00000006082, ENSAMEG00000007327, ENSAMEG00000011750, ENSAMEG00000012992, ENSAMEG00000013803, ENSAMEG00000015326, ENSAMEG00000015431, ENSAMEG00000017062, ENSAMEG00000017147, ENSAMEG00000017327, ENSAMEG00000017670, ENSAMEG00000018199, ENSAMEG00000019280, ENSAMEG00000019659, ENSAMEG00000020287	EPB41L3, KCNQ1, EPS8, INPPL1, RHOQ, TRIP10, HAX1, SNTA1, FES, MICALL2, LIMK1, ARRB1, FMNL2, FHOD1, PDLIM4, CDC42BPB, CDC42EP2, S1PR2, KCNE3
GO:0042133	neurotransmitter metabolic process	biological process	6/269	62/13500	0.00143	0.8293	ENSAMEG00000002398, ENSAMEG00000002581, ENSAMEG00000011617, ENSAMEG00000016224, ENSAMEG00000016637, ENSAMEG00000016662	CHDH, HNMT, COMT, CD36, DAGLA, DPYD
GO:0032634	interleukin-5 production	biological process	3/269	12/13500	0.00151	0.8293	ENSAMEG00000002415, ENSAMEG00000014490, ENSAMEG00000016820	IL17RB, IL5RA, IL25
GO:0060307	regulation of ventricular cardiac muscle cell membrane repolarization	biological process	3/269	12/13500	0.00151	0.8293	ENSAMEG00000002069, ENSAMEG00000012992, ENSAMEG00000020287	KCNQ1, SNTA1, KCNE3
GO:0097164	ammonium ion metabolic process	biological process	7/269	85/13500	0.00152	0.8293	ENSAMEG00000001272, ENSAMEG00000002314, ENSAMEG00000002398, ENSAMEG00000002581, ENSAMEG00000003089, ENSAMEG00000007468, ENSAMEG00000011617	CHKA, ENSAMEG00000002314, CHDH, HNMT, PNKD, SMPDL3A, COMT
GO:0002521	leukocyte differentiation	biological process	14/269	281/13500	0.00154	0.8293	ENSAMEG00000000272, ENSAMEG00000004441, ENSAMEG00000004774, ENSAMEG00000004811, ENSAMEG00000006928, ENSAMEG00000011750, ENSAMEG00000012594, ENSAMEG00000013931, ENSAMEG00000015180, ENSAMEG00000016820, ENSAMEG00000017184, ENSAMEG00000017745, ENSAMEG00000017990, ENSAMEG00000018841	CBFA2T3, GAS6, PPARG, POU2F2, DUSP10, HAX1, LYL1, RRAS, GATA2, IL25, APP, BATF2, BAD, CEBPG
GO:0010883	regulation of lipid storage	biological process	4/269	26/13500	0.00163	0.8293	ENSAMEG00000000943, ENSAMEG00000004774, ENSAMEG00000011831, ENSAMEG00000016224	LPL, PPARG, IKBKE, CD36
GO:2000116	regulation of cysteine-type endopeptidase activity	biological process	9/269	138/13500	0.00177	0.8293	ENSAMEG00000004441, ENSAMEG00000004774, ENSAMEG00000006630, ENSAMEG00000010287, ENSAMEG00000010991, ENSAMEG00000012590, ENSAMEG00000016947, ENSAMEG00000017062, ENSAMEG00000017990	GAS6, PPARG, NOD1, KIAA0141, HIP1, GPX1, NLRP3, ARRB1, BAD
GO:0007596	blood coagulation	biological process	8/269	114/13500	0.002	0.8293	ENSAMEG00000004441, ENSAMEG00000010841, ENSAMEG00000011403, ENSAMEG00000011687, ENSAMEG00000011842, ENSAMEG00000016015, ENSAMEG00000016224, ENSAMEG00000017422	GAS6, HSPB1, F5, SERPINE2, APOH, ACTG1, CD36, F13A1
GO:0006952	defense response	biological process	24/269	638/13500	0.00213	0.8293	ENSAMEG00000001502, ENSAMEG00000002415, ENSAMEG00000003641, ENSAMEG00000004196, ENSAMEG00000004663, ENSAMEG00000004774, ENSAMEG00000006630, ENSAMEG00000007284, ENSAMEG00000008499, ENSAMEG00000008575, ENSAMEG00000011225, ENSAMEG00000011820, ENSAMEG00000011831, ENSAMEG00000012283, ENSAMEG00000012590, ENSAMEG00000013869, ENSAMEG00000014335, ENSAMEG00000014490, ENSAMEG00000016224, ENSAMEG00000016820, ENSAMEG00000016947, ENSAMEG00000017745, ENSAMEG00000018841, ENSAMEG00000019893	ECM1, IL17RB, CARD9, SLPI, TRIM14, PPARG, NOD1, CYBB, ZFP36, STAB1, TMEM173, LYZ, IKBKE, SDHAF4, GPX1, A2M, ENSAMEG00000014335, IL5RA, CD36, IL25, NLRP3, BATF2, CEBPG, CYSLTR1
GO:0009611	response to wounding	biological process	13/269	260/13500	0.00218	0.8293	ENSAMEG00000004441, ENSAMEG00000006677, ENSAMEG00000008499, ENSAMEG00000010280, ENSAMEG00000010841, ENSAMEG00000011403, ENSAMEG00000011687, ENSAMEG00000011842, ENSAMEG00000012590, ENSAMEG00000012708, ENSAMEG00000016015, ENSAMEG00000016224, ENSAMEG00000017422	GAS6, NINJ1, ZFP36, ENSAMEG00000010280, HSPB1, F5, SERPINE2, APOH, GPX1, LAMB2, ACTG1, CD36, F13A1
GO:0007599	hemostasis	biological process	8/269	116/13500	0.00223	0.8293	ENSAMEG00000004441, ENSAMEG00000010841, ENSAMEG00000011403, ENSAMEG00000011687, ENSAMEG00000011842, ENSAMEG00000016015, ENSAMEG00000016224, ENSAMEG00000017422	GAS6, HSPB1, F5, SERPINE2, APOH, ACTG1, CD36, F13A1
GO:0042089	cytokine biosynthetic process	biological process	6/269	68/13500	0.0023	0.8293	ENSAMEG00000003641, ENSAMEG00000006630, ENSAMEG00000008499, ENSAMEG00000010841, ENSAMEG00000013744, ENSAMEG00000018841	CARD9, NOD1, ZFP36, HSPB1, MAST2, CEBPG
GO:0010757	negative regulation of plasminogen activation	biological process	2/269	4/13500	0.00231	0.8293	ENSAMEG00000011687, ENSAMEG00000012483	SERPINE2, CTSZ
GO:0010886	positive regulation of cholesterol storage	biological process	2/269	4/13500	0.00231	0.8293	ENSAMEG00000000943, ENSAMEG00000016224	LPL, CD36
GO:0106049	regulation of cellular response to osmotic stress	biological process	2/269	4/13500	0.00231	0.8293	ENSAMEG00000009393, ENSAMEG00000017990	YBX3, BAD
GO:1902218	regulation of intrinsic apoptotic signaling pathway in response to osmotic stress	biological process	2/269	4/13500	0.00231	0.8293	ENSAMEG00000009393, ENSAMEG00000017990	YBX3, BAD
GO:0050817	coagulation	biological process	8/269	117/13500	0.00235	0.8293	ENSAMEG00000004441, ENSAMEG00000010841, ENSAMEG00000011403, ENSAMEG00000011687, ENSAMEG00000011842, ENSAMEG00000016015, ENSAMEG00000016224, ENSAMEG00000017422	GAS6, HSPB1, F5, SERPINE2, APOH, ACTG1, CD36, F13A1
GO:0009141	nucleoside triphosphate metabolic process	biological process	10/269	172/13500	0.00236	0.8293	ENSAMEG00000000272, ENSAMEG00000003519, ENSAMEG00000006082, ENSAMEG00000007468, ENSAMEG00000011494, ENSAMEG00000017990, ENSAMEG00000023430, ENSAMEG00000023436, ENSAMEG00000023442, ENSAMEG00000023455	CBFA2T3, PFKFB2, RHOQ, SMPDL3A, FBP1, BAD, ND2, COX1, ATP6, CYTB
GO:0099623	regulation of cardiac muscle cell membrane repolarization	biological process	3/269	14/13500	0.00242	0.8293	ENSAMEG00000002069, ENSAMEG00000012992, ENSAMEG00000020287	KCNQ1, SNTA1, KCNE3
GO:0099625	ventricular cardiac muscle cell membrane repolarization	biological process	3/269	14/13500	0.00242	0.8293	ENSAMEG00000002069, ENSAMEG00000012992, ENSAMEG00000020287	KCNQ1, SNTA1, KCNE3
GO:0055086	nucleobase-containing small molecule metabolic process	biological process	18/269	431/13500	0.00254	0.8487	ENSAMEG00000000272, ENSAMEG00000003519, ENSAMEG00000006082, ENSAMEG00000007468, ENSAMEG00000007497, ENSAMEG00000008034, ENSAMEG00000009105, ENSAMEG00000010857, ENSAMEG00000011455, ENSAMEG00000011494, ENSAMEG00000014118, ENSAMEG00000015964, ENSAMEG00000016662, ENSAMEG00000017990, ENSAMEG00000023430, ENSAMEG00000023436, ENSAMEG00000023442, ENSAMEG00000023455	CBFA2T3, PFKFB2, RHOQ, SMPDL3A, NADSYN1, ENSAMEG00000008034, AHCY, ADCY9, PDE4A, FBP1, ENSAMEG00000014118, ADCY4, DPYD, BAD, ND2, COX1, ATP6, CYTB
GO:0030054	cell junction	cellular component	20/287	640/14310	0.03342	0.8682	ENSAMEG00000000602, ENSAMEG00000003894, ENSAMEG00000006453, ENSAMEG00000006768, ENSAMEG00000010536, ENSAMEG00000010841, ENSAMEG00000013263, ENSAMEG00000013297, ENSAMEG00000013803, ENSAMEG00000013931, ENSAMEG00000014689, ENSAMEG00000015326, ENSAMEG00000015431, ENSAMEG00000015837, ENSAMEG00000016015, ENSAMEG00000016087, ENSAMEG00000016396, ENSAMEG00000017184, ENSAMEG00000017327, ENSAMEG00000019485	EPB41L3, MRC2, ARHGAP21, CYTH3, VIM, HSPB1, REXO2, FHL2, FES, RRAS, TNS3, MICALL2, LIMK1, STXBP6, ACTG1, PDLIM1, DLG4, APP, FHOD1, CDC42EP4
GO:0042995	cell projection	cellular component	24/287	808/14310	0.03526	0.8682	ENSAMEG00000003086, ENSAMEG00000003627, ENSAMEG00000003733, ENSAMEG00000004387, ENSAMEG00000006768, ENSAMEG00000007278, ENSAMEG00000008620, ENSAMEG00000010536, ENSAMEG00000010841, ENSAMEG00000011509, ENSAMEG00000011750, ENSAMEG00000012095, ENSAMEG00000014724, ENSAMEG00000015252, ENSAMEG00000015431, ENSAMEG00000015964, ENSAMEG00000016224, ENSAMEG00000016396, ENSAMEG00000016637, ENSAMEG00000017062, ENSAMEG00000017184, ENSAMEG00000017670, ENSAMEG00000018345, ENSAMEG00000020287	NRP2, EPS8, INPP5E, PLXND1, CYTH3, ARHGAP31, CYBRD1, VIM, HSPB1, SEPT5, HAX1, NAV1, ITSN1, RILPL1, LIMK1, ADCY4, CD36, DLG4, DAGLA, ARRB1, APP, PDLIM4, TTLL5, KCNE3
GO:0015629	actin cytoskeleton	cellular component	11/287	294/14310	0.03531	0.8682	ENSAMEG00000005486, ENSAMEG00000006082, ENSAMEG00000006453, ENSAMEG00000011202, ENSAMEG00000011750, ENSAMEG00000015326, ENSAMEG00000016015, ENSAMEG00000017327, ENSAMEG00000017670, ENSAMEG00000018199, ENSAMEG00000019485	SPPL2B, RHOQ, ARHGAP21, ZNF74, HAX1, MICALL2, ACTG1, FHOD1, PDLIM4, CDC42BPB, CDC42EP4
GO:0070469	respiratory chain	cellular component	4/287	63/14310	0.03751	0.8682	ENSAMEG00000023426, ENSAMEG00000023430, ENSAMEG00000023436, ENSAMEG00000023455	ND1, ND2, COX1, CYTB
GO:0001725	stress fiber	cellular component	3/287	37/14310	0.03757	0.8682	ENSAMEG00000015326, ENSAMEG00000017327, ENSAMEG00000017670	MICALL2, FHOD1, PDLIM4
GO:0097517	contractile actin filament bundle	cellular component	3/287	37/14310	0.03757	0.8682	ENSAMEG00000015326, ENSAMEG00000017327, ENSAMEG00000017670	MICALL2, FHOD1, PDLIM4
GO:0044429	mitochondrial part	cellular component	16/287	491/14310	0.03918	0.8682	ENSAMEG00000001814, ENSAMEG00000002314, ENSAMEG00000002398, ENSAMEG00000006014, ENSAMEG00000006540, ENSAMEG00000008424, ENSAMEG00000011225, ENSAMEG00000011750, ENSAMEG00000011831, ENSAMEG00000016446, ENSAMEG00000017990, ENSAMEG00000023426, ENSAMEG00000023430, ENSAMEG00000023436, ENSAMEG00000023442, ENSAMEG00000023455	HADH, ENSAMEG00000002314, CHDH, RAB32, GLRX5, RNF144B, TMEM173, HAX1, IKBKE, EFHD1, BAD, ND1, ND2, COX1, ATP6, CYTB
GO:0005737	cytoplasm	cellular component	140/287	6226/14310	0.03957	0.8682	ENSAMEG00000000640, ENSAMEG00000001230, ENSAMEG00000001276, ENSAMEG00000001784, ENSAMEG00000001814, ENSAMEG00000002069, ENSAMEG00000002314, ENSAMEG00000002398, ENSAMEG00000002415, ENSAMEG00000002444, ENSAMEG00000002535, ENSAMEG00000002581, ENSAMEG00000002732, ENSAMEG00000003020, ENSAMEG00000003089, ENSAMEG00000003442, ENSAMEG00000003625, ENSAMEG00000003627, ENSAMEG00000003641, ENSAMEG00000003733, ENSAMEG00000004196, ENSAMEG00000004348, ENSAMEG00000004441, ENSAMEG00000004516, ENSAMEG00000004680, ENSAMEG00000004774, ENSAMEG00000005113, ENSAMEG00000005486, ENSAMEG00000005566, ENSAMEG00000005610, ENSAMEG00000005798, ENSAMEG00000005874, ENSAMEG00000005927, ENSAMEG00000006014, ENSAMEG00000006083, ENSAMEG00000006453, ENSAMEG00000006540, ENSAMEG00000006630, ENSAMEG00000006768, ENSAMEG00000006928, ENSAMEG00000007046, ENSAMEG00000007497, ENSAMEG00000007997, ENSAMEG00000008424, ENSAMEG00000008477, ENSAMEG00000008497, ENSAMEG00000008499, ENSAMEG00000008800, ENSAMEG00000009105, ENSAMEG00000009193, ENSAMEG00000009337, ENSAMEG00000009367, ENSAMEG00000009393, ENSAMEG00000009555, ENSAMEG00000009824, ENSAMEG00000009860, ENSAMEG00000009928, ENSAMEG00000010113, ENSAMEG00000010219, ENSAMEG00000010287, ENSAMEG00000010536, ENSAMEG00000010841, ENSAMEG00000010991, ENSAMEG00000011170, ENSAMEG00000011225, ENSAMEG00000011403, ENSAMEG00000011455, ENSAMEG00000011494, ENSAMEG00000011509, ENSAMEG00000011582, ENSAMEG00000011617, ENSAMEG00000011687, ENSAMEG00000011750, ENSAMEG00000011792, ENSAMEG00000011831, ENSAMEG00000012016, ENSAMEG00000012283, ENSAMEG00000012418, ENSAMEG00000012425, ENSAMEG00000012457, ENSAMEG00000012483, ENSAMEG00000012576, ENSAMEG00000012590, ENSAMEG00000012689, ENSAMEG00000012804, ENSAMEG00000013098, ENSAMEG00000013162, ENSAMEG00000013263, ENSAMEG00000013478, ENSAMEG00000013803, ENSAMEG00000014605, ENSAMEG00000014637, ENSAMEG00000014678, ENSAMEG00000014689, ENSAMEG00000014724, ENSAMEG00000015156, ENSAMEG00000015157, ENSAMEG00000015221, ENSAMEG00000015252, ENSAMEG00000015326, ENSAMEG00000015346, ENSAMEG00000015431, ENSAMEG00000015604, ENSAMEG00000015938, ENSAMEG00000015964, ENSAMEG00000016015, ENSAMEG00000016224, ENSAMEG00000016339, ENSAMEG00000016396, ENSAMEG00000016446, ENSAMEG00000016662, ENSAMEG00000016729, ENSAMEG00000016736, ENSAMEG00000016765, ENSAMEG00000016795, ENSAMEG00000016947, ENSAMEG00000017062, ENSAMEG00000017107, ENSAMEG00000017184, ENSAMEG00000017327, ENSAMEG00000017441, ENSAMEG00000017670, ENSAMEG00000017699, ENSAMEG00000017739, ENSAMEG00000017862, ENSAMEG00000017964, ENSAMEG00000017990, ENSAMEG00000018186, ENSAMEG00000018345, ENSAMEG00000019280, ENSAMEG00000019485, ENSAMEG00000019719, ENSAMEG00000020108, ENSAMEG00000020281, ENSAMEG00000020287, ENSAMEG00000023426, ENSAMEG00000023430, ENSAMEG00000023436, ENSAMEG00000023442, ENSAMEG00000023455	HSPB6, TCIRG1, ZNRF1, TTC7B, HADH, KCNQ1, ENSAMEG00000002314, CHDH, IL17RB, CRTAP, ANPEP, HNMT, PLOD3, HACD4, PNKD, CRYZ, SBF2, EPS8, CARD9, INPP5E, SLPI, ENSAMEG00000004348, GAS6, CD1D, NANS, PPARG, SCD5, SPPL2B, ADAP2, INPPL1, JAZF1, TTC25, ATP10A, RAB32, AP2A2, ARHGAP21, GLRX5, NOD1, CYTH3, DUSP10, TMEM8A, NADSYN1, CLCN7, RNF144B, HSCB, STEAP3, ZFP36, DGKH, AHCY, DMXL2, ZEB2, XXYLT1, YBX3, CPT2, SOCS2, ORMDL3, TRIM47, SLA2, GNPDA1, KIAA0141, VIM, HSPB1, HIP1, PGPEP1, TMEM173, F5, PDE4A, FBP1, SEPT5, CCDC50, COMT, SERPINE2, HAX1, ENSAMEG00000011792, IKBKE, ANGPTL6, SDHAF4, ERCC1, RNF7, BLVRB, CTSZ, DNAJA4, GPX1, PORCN, C3orf58, ENSAMEG00000013098, ACSS3, REXO2, SNAI1, FES, NAAA, UNC45B, PPP1R15A, TNS3, ITSN1, SERPINB10, GAA, SGSH, RILPL1, MICALL2, CLIP2, LIMK1, NUAK1, HFE, ADCY4, ACTG1, CD36, TRIM3, DLG4, EFHD1, DPYD, TNFSF12, TNFSF13, CD68, TMEM175, NLRP3, ARRB1, RIN1, APP, FHOD1, ENSAMEG00000017441, PDLIM4, CA5B, SLC29A3, ABCA3, PTGR1, BAD, TNFAIP2, TTLL5, CDC42EP2, CDC42EP4, B3GNT9, OST4, B3GALT2, KCNE3, ND1, ND2, COX1, ATP6, CYTB
GO:0000110	nucleotide-excision repair factor 1 complex	cellular component	1/287	2/14310	0.03971	0.8682	ENSAMEG00000012418	ERCC1
GO:0031232	extrinsic component of external side of plasma membrane	cellular component	1/287	2/14310	0.03971	0.8682	ENSAMEG00000011687	SERPINE2
GO:0035339	SPOTS complex	cellular component	1/287	2/14310	0.03971	0.8682	ENSAMEG00000009860	ORMDL3
GO:0043083	synaptic cleft	cellular component	1/287	2/14310	0.03971	0.8682	ENSAMEG00000012708	LAMB2
GO:0072559	NLRP3 inflammasome complex	cellular component	1/287	2/14310	0.03971	0.8682	ENSAMEG00000016947	NLRP3
GO:0042107	cytokine metabolic process	biological process	6/269	70/13500	0.00267	0.8704	ENSAMEG00000003641, ENSAMEG00000006630, ENSAMEG00000008499, ENSAMEG00000010841, ENSAMEG00000013744, ENSAMEG00000018841	CARD9, NOD1, ZFP36, HSPB1, MAST2, CEBPG
GO:0043393	regulation of protein binding	biological process	9/269	148/13500	0.00286	0.882	ENSAMEG00000004196, ENSAMEG00000004387, ENSAMEG00000011225, ENSAMEG00000012483, ENSAMEG00000015938, ENSAMEG00000016224, ENSAMEG00000016268, ENSAMEG00000017062, ENSAMEG00000017184	SLPI, PLXND1, TMEM173, CTSZ, HFE, CD36, CAMK1, ARRB1, APP
GO:0032640	tumor necrosis factor production	biological process	6/269	71/13500	0.00287	0.882	ENSAMEG00000003641, ENSAMEG00000004441, ENSAMEG00000006630, ENSAMEG00000010841, ENSAMEG00000016224, ENSAMEG00000017184	CARD9, GAS6, NOD1, HSPB1, CD36, APP
GO:0010884	positive regulation of lipid storage	biological process	3/269	15/13500	0.00298	0.882	ENSAMEG00000000943, ENSAMEG00000011831, ENSAMEG00000016224	LPL, IKBKE, CD36
GO:0051239	regulation of multicellular organismal process	biological process	47/269	1581/13500	0.00314	0.882	ENSAMEG00000000528, ENSAMEG00000000640, ENSAMEG00000000943, ENSAMEG00000001502, ENSAMEG00000002069, ENSAMEG00000002415, ENSAMEG00000003641, ENSAMEG00000004387, ENSAMEG00000004441, ENSAMEG00000004774, ENSAMEG00000006630, ENSAMEG00000006928, ENSAMEG00000008416, ENSAMEG00000008499, ENSAMEG00000008575, ENSAMEG00000009824, ENSAMEG00000010280, ENSAMEG00000010536, ENSAMEG00000010841, ENSAMEG00000011225, ENSAMEG00000011687, ENSAMEG00000011750, ENSAMEG00000011842, ENSAMEG00000012590, ENSAMEG00000012943, ENSAMEG00000012992, ENSAMEG00000013478, ENSAMEG00000013744, ENSAMEG00000013803, ENSAMEG00000013931, ENSAMEG00000014318, ENSAMEG00000014490, ENSAMEG00000015157, ENSAMEG00000015180, ENSAMEG00000015431, ENSAMEG00000015938, ENSAMEG00000016224, ENSAMEG00000016268, ENSAMEG00000016625, ENSAMEG00000016736, ENSAMEG00000016947, ENSAMEG00000017062, ENSAMEG00000017184, ENSAMEG00000017990, ENSAMEG00000018841, ENSAMEG00000019659, ENSAMEG00000020287	NCKIPSD, HSPB6, LPL, ECM1, KCNQ1, IL17RB, CARD9, PLXND1, GAS6, PPARG, NOD1, DUSP10, KREMEN1, ZFP36, STAB1, SOCS2, ENSAMEG00000010280, VIM, HSPB1, TMEM173, SERPINE2, HAX1, APOH, GPX1, TIMP1, SNTA1, SNAI1, MAST2, FES, RRAS, ACVR1, IL5RA, GAA, GATA2, LIMK1, HFE, CD36, CAMK1, PBX3, TNFSF13, NLRP3, ARRB1, APP, BAD, CEBPG, S1PR2, KCNE3
GO:0007585	respiratory gaseous exchange	biological process	4/269	31/13500	0.00318	0.882	ENSAMEG00000004600, ENSAMEG00000010280, ENSAMEG00000015157, ENSAMEG00000016625	NDST1, ENSAMEG00000010280, GAA, PBX3
GO:0050830	defense response to Gram-positive bacterium	biological process	4/269	31/13500	0.00318	0.882	ENSAMEG00000003641, ENSAMEG00000006630, ENSAMEG00000011820, ENSAMEG00000016224	CARD9, NOD1, LYZ, CD36
GO:0071706	tumor necrosis factor superfamily cytokine production	biological process	6/269	74/13500	0.00353	0.882	ENSAMEG00000003641, ENSAMEG00000004441, ENSAMEG00000006630, ENSAMEG00000010841, ENSAMEG00000016224, ENSAMEG00000017184	CARD9, GAS6, NOD1, HSPB1, CD36, APP
GO:0009205	purine ribonucleoside triphosphate metabolic process	biological process	9/269	153/13500	0.00357	0.882	ENSAMEG00000000272, ENSAMEG00000003519, ENSAMEG00000006082, ENSAMEG00000011494, ENSAMEG00000017990, ENSAMEG00000023430, ENSAMEG00000023436, ENSAMEG00000023442, ENSAMEG00000023455	CBFA2T3, PFKFB2, RHOQ, FBP1, BAD, ND2, COX1, ATP6, CYTB
GO:0031638	zymogen activation	biological process	4/269	32/13500	0.00357	0.882	ENSAMEG00000011687, ENSAMEG00000011842, ENSAMEG00000012483, ENSAMEG00000017990	SERPINE2, APOH, CTSZ, BAD
GO:0001816	cytokine production	biological process	17/269	411/13500	0.00366	0.882	ENSAMEG00000000943, ENSAMEG00000002415, ENSAMEG00000003641, ENSAMEG00000004441, ENSAMEG00000006630, ENSAMEG00000008499, ENSAMEG00000010841, ENSAMEG00000011225, ENSAMEG00000013744, ENSAMEG00000014490, ENSAMEG00000015938, ENSAMEG00000016224, ENSAMEG00000016820, ENSAMEG00000016947, ENSAMEG00000017062, ENSAMEG00000017184, ENSAMEG00000018841	LPL, IL17RB, CARD9, GAS6, NOD1, ZFP36, HSPB1, TMEM173, MAST2, IL5RA, HFE, CD36, IL25, NLRP3, ARRB1, APP, CEBPG
GO:0035754	B cell chemotaxis	biological process	2/269	5/13500	0.0038	0.882	ENSAMEG00000004441, ENSAMEG00000007512	GAS6, HSD3B7
GO:0090240	positive regulation of histone H4 acetylation	biological process	2/269	5/13500	0.0038	0.882	ENSAMEG00000005777, ENSAMEG00000017062	AUTS2, ARRB1
GO:0045765	regulation of angiogenesis	biological process	9/269	155/13500	0.00389	0.882	ENSAMEG00000000640, ENSAMEG00000001502, ENSAMEG00000004387, ENSAMEG00000004774, ENSAMEG00000008575, ENSAMEG00000010841, ENSAMEG00000011842, ENSAMEG00000013931, ENSAMEG00000015180	HSPB6, ECM1, PLXND1, PPARG, STAB1, HSPB1, APOH, RRAS, GATA2
GO:0051240	positive regulation of multicellular organismal process	biological process	29/269	863/13500	0.004	0.882	ENSAMEG00000000528, ENSAMEG00000000640, ENSAMEG00000000943, ENSAMEG00000001502, ENSAMEG00000002069, ENSAMEG00000002415, ENSAMEG00000003641, ENSAMEG00000004441, ENSAMEG00000006630, ENSAMEG00000006928, ENSAMEG00000009824, ENSAMEG00000010841, ENSAMEG00000011225, ENSAMEG00000011687, ENSAMEG00000011750, ENSAMEG00000011842, ENSAMEG00000013478, ENSAMEG00000013803, ENSAMEG00000013931, ENSAMEG00000014318, ENSAMEG00000015180, ENSAMEG00000015431, ENSAMEG00000016224, ENSAMEG00000016268, ENSAMEG00000016736, ENSAMEG00000016947, ENSAMEG00000017184, ENSAMEG00000017990, ENSAMEG00000018841	NCKIPSD, HSPB6, LPL, ECM1, KCNQ1, IL17RB, CARD9, GAS6, NOD1, DUSP10, SOCS2, HSPB1, TMEM173, SERPINE2, HAX1, APOH, SNAI1, FES, RRAS, ACVR1, GATA2, LIMK1, CD36, CAMK1, TNFSF13, NLRP3, APP, BAD, CEBPG
GO:0009199	ribonucleoside triphosphate metabolic process	biological process	9/269	157/13500	0.00423	0.882	ENSAMEG00000000272, ENSAMEG00000003519, ENSAMEG00000006082, ENSAMEG00000011494, ENSAMEG00000017990, ENSAMEG00000023430, ENSAMEG00000023436, ENSAMEG00000023442, ENSAMEG00000023455	CBFA2T3, PFKFB2, RHOQ, FBP1, BAD, ND2, COX1, ATP6, CYTB
GO:0042060	wound healing	biological process	11/269	218/13500	0.00436	0.882	ENSAMEG00000004441, ENSAMEG00000006677, ENSAMEG00000010280, ENSAMEG00000010841, ENSAMEG00000011403, ENSAMEG00000011687, ENSAMEG00000011842, ENSAMEG00000012590, ENSAMEG00000016015, ENSAMEG00000016224, ENSAMEG00000017422	GAS6, NINJ1, ENSAMEG00000010280, HSPB1, F5, SERPINE2, APOH, GPX1, ACTG1, CD36, F13A1
GO:0009144	purine nucleoside triphosphate metabolic process	biological process	9/269	158/13500	0.00441	0.882	ENSAMEG00000000272, ENSAMEG00000003519, ENSAMEG00000006082, ENSAMEG00000011494, ENSAMEG00000017990, ENSAMEG00000023430, ENSAMEG00000023436, ENSAMEG00000023442, ENSAMEG00000023455	CBFA2T3, PFKFB2, RHOQ, FBP1, BAD, ND2, COX1, ATP6, CYTB
GO:0006753	nucleoside phosphate metabolic process	biological process	16/269	388/13500	0.0049	0.882	ENSAMEG00000000272, ENSAMEG00000003519, ENSAMEG00000006082, ENSAMEG00000007468, ENSAMEG00000007497, ENSAMEG00000008034, ENSAMEG00000010857, ENSAMEG00000011455, ENSAMEG00000011494, ENSAMEG00000014118, ENSAMEG00000015964, ENSAMEG00000017990, ENSAMEG00000023430, ENSAMEG00000023436, ENSAMEG00000023442, ENSAMEG00000023455	CBFA2T3, PFKFB2, RHOQ, SMPDL3A, NADSYN1, ENSAMEG00000008034, ADCY9, PDE4A, FBP1, ENSAMEG00000014118, ADCY4, BAD, ND2, COX1, ATP6, CYTB
GO:0032612	interleukin-1 production	biological process	4/269	35/13500	0.00497	0.882	ENSAMEG00000004441, ENSAMEG00000010841, ENSAMEG00000016224, ENSAMEG00000016947	GAS6, HSPB1, CD36, NLRP3
GO:0051346	negative regulation of hydrolase activity	biological process	12/269	254/13500	0.00501	0.882	ENSAMEG00000001502, ENSAMEG00000004196, ENSAMEG00000004441, ENSAMEG00000004836, ENSAMEG00000010076, ENSAMEG00000011687, ENSAMEG00000012590, ENSAMEG00000012943, ENSAMEG00000013869, ENSAMEG00000014678, ENSAMEG00000017062, ENSAMEG00000017184	ECM1, SLPI, GAS6, TIMP4, ENSAMEG00000010076, SERPINE2, GPX1, TIMP1, A2M, PPP1R15A, ARRB1, APP
GO:0010955	negative regulation of protein processing	biological process	3/269	18/13500	0.00511	0.882	ENSAMEG00000011687, ENSAMEG00000012483, ENSAMEG00000013869	SERPINE2, CTSZ, A2M
GO:0014002	astrocyte development	biological process	3/269	18/13500	0.00511	0.882	ENSAMEG00000010536, ENSAMEG00000012708, ENSAMEG00000017184	VIM, LAMB2, APP
GO:0099622	cardiac muscle cell membrane repolarization	biological process	3/269	18/13500	0.00511	0.882	ENSAMEG00000002069, ENSAMEG00000012992, ENSAMEG00000020287	KCNQ1, SNTA1, KCNE3
GO:1903318	negative regulation of protein maturation	biological process	3/269	18/13500	0.00511	0.882	ENSAMEG00000011687, ENSAMEG00000012483, ENSAMEG00000013869	SERPINE2, CTSZ, A2M
GO:0008627	intrinsic apoptotic signaling pathway in response to osmotic stress	biological process	2/269	6/13500	0.00563	0.882	ENSAMEG00000009393, ENSAMEG00000017990	YBX3, BAD
GO:0015858	nucleoside transport	biological process	2/269	6/13500	0.00563	0.882	ENSAMEG00000011849, ENSAMEG00000017739	SLC29A1, SLC29A3
GO:0018095	protein polyglutamylation	biological process	2/269	6/13500	0.00563	0.882	ENSAMEG00000002683, ENSAMEG00000018345	TPGS1, TTLL5
GO:0047484	regulation of response to osmotic stress	biological process	2/269	6/13500	0.00563	0.882	ENSAMEG00000009393, ENSAMEG00000017990	YBX3, BAD
GO:0050711	negative regulation of interleukin-1 secretion	biological process	2/269	6/13500	0.00563	0.882	ENSAMEG00000004441, ENSAMEG00000016947	GAS6, NLRP3
GO:0097623	potassium ion export across plasma membrane	biological process	2/269	6/13500	0.00563	0.882	ENSAMEG00000002069, ENSAMEG00000020287	KCNQ1, KCNE3
GO:1901642	nucleoside transmembrane transport	biological process	2/269	6/13500	0.00563	0.882	ENSAMEG00000011849, ENSAMEG00000017739	SLC29A1, SLC29A3
GO:0019359	nicotinamide nucleotide biosynthetic process	biological process	5/269	58/13500	0.00587	0.882	ENSAMEG00000000272, ENSAMEG00000003519, ENSAMEG00000007497, ENSAMEG00000008034, ENSAMEG00000011494	CBFA2T3, PFKFB2, NADSYN1, ENSAMEG00000008034, FBP1
GO:0019363	pyridine nucleotide biosynthetic process	biological process	5/269	58/13500	0.00587	0.882	ENSAMEG00000000272, ENSAMEG00000003519, ENSAMEG00000007497, ENSAMEG00000008034, ENSAMEG00000011494	CBFA2T3, PFKFB2, NADSYN1, ENSAMEG00000008034, FBP1
GO:0046034	ATP metabolic process	biological process	8/269	136/13500	0.00588	0.882	ENSAMEG00000000272, ENSAMEG00000003519, ENSAMEG00000011494, ENSAMEG00000017990, ENSAMEG00000023430, ENSAMEG00000023436, ENSAMEG00000023442, ENSAMEG00000023455	CBFA2T3, PFKFB2, FBP1, BAD, ND2, COX1, ATP6, CYTB
GO:0030036	actin cytoskeleton organization	biological process	16/269	396/13500	0.00593	0.882	ENSAMEG00000000602, ENSAMEG00000003627, ENSAMEG00000005610, ENSAMEG00000006082, ENSAMEG00000007327, ENSAMEG00000011750, ENSAMEG00000013803, ENSAMEG00000015326, ENSAMEG00000015431, ENSAMEG00000017062, ENSAMEG00000017147, ENSAMEG00000017327, ENSAMEG00000017670, ENSAMEG00000018199, ENSAMEG00000019280, ENSAMEG00000019659	EPB41L3, EPS8, INPPL1, RHOQ, TRIP10, HAX1, FES, MICALL2, LIMK1, ARRB1, FMNL2, FHOD1, PDLIM4, CDC42BPB, CDC42EP2, S1PR2
GO:0031639	plasminogen activation	biological process	3/269	19/13500	0.00599	0.882	ENSAMEG00000011687, ENSAMEG00000011842, ENSAMEG00000012483	SERPINE2, APOH, CTSZ
GO:0086005	ventricular cardiac muscle cell action potential	biological process	3/269	19/13500	0.00599	0.882	ENSAMEG00000002069, ENSAMEG00000012992, ENSAMEG00000020287	KCNQ1, SNTA1, KCNE3
GO:0072521	purine-containing compound metabolic process	biological process	14/269	327/13500	0.00601	0.882	ENSAMEG00000000272, ENSAMEG00000003519, ENSAMEG00000006082, ENSAMEG00000009105, ENSAMEG00000010857, ENSAMEG00000011455, ENSAMEG00000011494, ENSAMEG00000015964, ENSAMEG00000016662, ENSAMEG00000017990, ENSAMEG00000023430, ENSAMEG00000023436, ENSAMEG00000023442, ENSAMEG00000023455	CBFA2T3, PFKFB2, RHOQ, AHCY, ADCY9, PDE4A, FBP1, ADCY4, DPYD, BAD, ND2, COX1, ATP6, CYTB
GO:0044092	negative regulation of molecular function	biological process	23/269	655/13500	0.00605	0.882	ENSAMEG00000001502, ENSAMEG00000004196, ENSAMEG00000004441, ENSAMEG00000004836, ENSAMEG00000005412, ENSAMEG00000006928, ENSAMEG00000008499, ENSAMEG00000010076, ENSAMEG00000010841, ENSAMEG00000011687, ENSAMEG00000012483, ENSAMEG00000012590, ENSAMEG00000012943, ENSAMEG00000013869, ENSAMEG00000014678, ENSAMEG00000015431, ENSAMEG00000015938, ENSAMEG00000016268, ENSAMEG00000016947, ENSAMEG00000017062, ENSAMEG00000017184, ENSAMEG00000018841, ENSAMEG00000020287	ECM1, SLPI, GAS6, TIMP4, ADORA3, DUSP10, ZFP36, ENSAMEG00000010076, HSPB1, SERPINE2, CTSZ, GPX1, TIMP1, A2M, PPP1R15A, LIMK1, HFE, CAMK1, NLRP3, ARRB1, APP, CEBPG, KCNE3
GO:0032760	positive regulation of tumor necrosis factor production	biological process	4/269	37/13500	0.00607	0.882	ENSAMEG00000003641, ENSAMEG00000006630, ENSAMEG00000010841, ENSAMEG00000016224	CARD9, NOD1, HSPB1, CD36
GO:0045597	positive regulation of cell differentiation	biological process	18/269	469/13500	0.00612	0.882	ENSAMEG00000000528, ENSAMEG00000004441, ENSAMEG00000006928, ENSAMEG00000008499, ENSAMEG00000009824, ENSAMEG00000011687, ENSAMEG00000011750, ENSAMEG00000013478, ENSAMEG00000013803, ENSAMEG00000014318, ENSAMEG00000015180, ENSAMEG00000015431, ENSAMEG00000016224, ENSAMEG00000016268, ENSAMEG00000017184, ENSAMEG00000017238, ENSAMEG00000017990, ENSAMEG00000019659	NCKIPSD, GAS6, DUSP10, ZFP36, SOCS2, SERPINE2, HAX1, SNAI1, FES, ACVR1, GATA2, LIMK1, CD36, CAMK1, APP, HSF4, BAD, S1PR2
GO:1901342	regulation of vasculature development	biological process	9/269	167/13500	0.0063	0.882	ENSAMEG00000000640, ENSAMEG00000001502, ENSAMEG00000004387, ENSAMEG00000004774, ENSAMEG00000008575, ENSAMEG00000010841, ENSAMEG00000011842, ENSAMEG00000013931, ENSAMEG00000015180	HSPB6, ECM1, PLXND1, PPARG, STAB1, HSPB1, APOH, RRAS, GATA2
GO:0042035	regulation of cytokine biosynthetic process	biological process	5/269	59/13500	0.00631	0.882	ENSAMEG00000003641, ENSAMEG00000008499, ENSAMEG00000010841, ENSAMEG00000013744, ENSAMEG00000018841	CARD9, ZFP36, HSPB1, MAST2, CEBPG
GO:1903557	positive regulation of tumor necrosis factor superfamily cytokine production	biological process	4/269	38/13500	0.00668	0.882	ENSAMEG00000003641, ENSAMEG00000006630, ENSAMEG00000010841, ENSAMEG00000016224	CARD9, NOD1, HSPB1, CD36
GO:0019439	aromatic compound catabolic process	biological process	12/269	264/13500	0.00676	0.882	ENSAMEG00000000272, ENSAMEG00000002581, ENSAMEG00000003519, ENSAMEG00000005484, ENSAMEG00000007468, ENSAMEG00000008499, ENSAMEG00000009105, ENSAMEG00000011455, ENSAMEG00000011494, ENSAMEG00000011617, ENSAMEG00000012457, ENSAMEG00000016662	CBFA2T3, HNMT, PFKFB2, LSM7, SMPDL3A, ZFP36, AHCY, PDE4A, FBP1, COMT, BLVRB, DPYD
GO:0003016	respiratory system process	biological process	3/269	20/13500	0.00694	0.882	ENSAMEG00000010280, ENSAMEG00000015157, ENSAMEG00000016625	ENSAMEG00000010280, GAA, PBX3
GO:0042246	tissue regeneration	biological process	3/269	20/13500	0.00694	0.882	ENSAMEG00000006677, ENSAMEG00000010280, ENSAMEG00000012590	NINJ1, ENSAMEG00000010280, GPX1
GO:0060306	regulation of membrane repolarization	biological process	3/269	20/13500	0.00694	0.882	ENSAMEG00000002069, ENSAMEG00000012992, ENSAMEG00000020287	KCNQ1, SNTA1, KCNE3
GO:0072525	pyridine-containing compound biosynthetic process	biological process	5/269	61/13500	0.00726	0.882	ENSAMEG00000000272, ENSAMEG00000003519, ENSAMEG00000007497, ENSAMEG00000008034, ENSAMEG00000011494	CBFA2T3, PFKFB2, NADSYN1, ENSAMEG00000008034, FBP1
GO:0019637	organophosphate metabolic process	biological process	22/269	627/13500	0.00726	0.882	ENSAMEG00000000272, ENSAMEG00000001272, ENSAMEG00000001784, ENSAMEG00000002314, ENSAMEG00000003519, ENSAMEG00000003733, ENSAMEG00000005610, ENSAMEG00000006082, ENSAMEG00000007468, ENSAMEG00000007497, ENSAMEG00000008034, ENSAMEG00000008800, ENSAMEG00000010857, ENSAMEG00000011455, ENSAMEG00000011494, ENSAMEG00000014118, ENSAMEG00000015964, ENSAMEG00000017990, ENSAMEG00000023430, ENSAMEG00000023436, ENSAMEG00000023442, ENSAMEG00000023455	CBFA2T3, CHKA, TTC7B, ENSAMEG00000002314, PFKFB2, INPP5E, INPPL1, RHOQ, SMPDL3A, NADSYN1, ENSAMEG00000008034, DGKH, ADCY9, PDE4A, FBP1, ENSAMEG00000014118, ADCY4, BAD, ND2, COX1, ATP6, CYTB
GO:0046486	glycerolipid metabolic process	biological process	10/269	202/13500	0.00733	0.882	ENSAMEG00000000943, ENSAMEG00000001272, ENSAMEG00000001784, ENSAMEG00000002314, ENSAMEG00000003733, ENSAMEG00000005610, ENSAMEG00000008800, ENSAMEG00000011842, ENSAMEG00000012590, ENSAMEG00000016637	LPL, CHKA, TTC7B, ENSAMEG00000002314, INPP5E, INPPL1, DGKH, APOH, GPX1, DAGLA
GO:1901135	carbohydrate derivative metabolic process	biological process	23/269	667/13500	0.00747	0.882	ENSAMEG00000000272, ENSAMEG00000002732, ENSAMEG00000003519, ENSAMEG00000004600, ENSAMEG00000006082, ENSAMEG00000009105, ENSAMEG00000009367, ENSAMEG00000010219, ENSAMEG00000010857, ENSAMEG00000011455, ENSAMEG00000011494, ENSAMEG00000012689, ENSAMEG00000015221, ENSAMEG00000015964, ENSAMEG00000016662, ENSAMEG00000017990, ENSAMEG00000019719, ENSAMEG00000020108, ENSAMEG00000020281, ENSAMEG00000023430, ENSAMEG00000023436, ENSAMEG00000023442, ENSAMEG00000023455	CBFA2T3, PLOD3, PFKFB2, NDST1, RHOQ, AHCY, XXYLT1, GNPDA1, ADCY9, PDE4A, FBP1, PORCN, SGSH, ADCY4, DPYD, BAD, B3GNT9, OST4, B3GALT2, ND2, COX1, ATP6, CYTB
GO:0030812	negative regulation of nucleotide catabolic process	biological process	2/269	7/13500	0.00778	0.882	ENSAMEG00000000272, ENSAMEG00000011494	CBFA2T3, FBP1
GO:0031269	pseudopodium assembly	biological process	2/269	7/13500	0.00778	0.882	ENSAMEG00000019280, ENSAMEG00000019485	CDC42EP2, CDC42EP4
GO:0031272	regulation of pseudopodium assembly	biological process	2/269	7/13500	0.00778	0.882	ENSAMEG00000019280, ENSAMEG00000019485	CDC42EP2, CDC42EP4
GO:0031274	positive regulation of pseudopodium assembly	biological process	2/269	7/13500	0.00778	0.882	ENSAMEG00000019280, ENSAMEG00000019485	CDC42EP2, CDC42EP4
GO:0045820	negative regulation of glycolytic process	biological process	2/269	7/13500	0.00778	0.882	ENSAMEG00000000272, ENSAMEG00000011494	CBFA2T3, FBP1
GO:0051195	negative regulation of cofactor metabolic process	biological process	2/269	7/13500	0.00778	0.882	ENSAMEG00000000272, ENSAMEG00000011494	CBFA2T3, FBP1
GO:0051198	negative regulation of coenzyme metabolic process	biological process	2/269	7/13500	0.00778	0.882	ENSAMEG00000000272, ENSAMEG00000011494	CBFA2T3, FBP1
GO:0071404	cellular response to low-density lipoprotein particle stimulus	biological process	2/269	7/13500	0.00778	0.882	ENSAMEG00000000943, ENSAMEG00000016224	LPL, CD36
GO:0072378	blood coagulation, fibrin clot formation	biological process	2/269	7/13500	0.00778	0.882	ENSAMEG00000011842, ENSAMEG00000017422	APOH, F13A1
GO:0098915	membrane repolarization during ventricular cardiac muscle cell action potential	biological process	2/269	7/13500	0.00778	0.882	ENSAMEG00000002069, ENSAMEG00000020287	KCNQ1, KCNE3
GO:0001817	regulation of cytokine production	biological process	15/269	373/13500	0.00793	0.882	ENSAMEG00000000943, ENSAMEG00000002415, ENSAMEG00000003641, ENSAMEG00000004441, ENSAMEG00000006630, ENSAMEG00000008499, ENSAMEG00000010841, ENSAMEG00000011225, ENSAMEG00000013744, ENSAMEG00000014490, ENSAMEG00000015938, ENSAMEG00000016224, ENSAMEG00000016947, ENSAMEG00000017062, ENSAMEG00000018841	LPL, IL17RB, CARD9, GAS6, NOD1, ZFP36, HSPB1, TMEM173, MAST2, IL5RA, HFE, CD36, NLRP3, ARRB1, CEBPG
GO:0048708	astrocyte differentiation	biological process	4/269	40/13500	0.00802	0.882	ENSAMEG00000010536, ENSAMEG00000011687, ENSAMEG00000012708, ENSAMEG00000017184	VIM, SERPINE2, LAMB2, APP
GO:0002573	myeloid leukocyte differentiation	biological process	7/269	115/13500	0.00814	0.882	ENSAMEG00000000272, ENSAMEG00000004774, ENSAMEG00000011750, ENSAMEG00000015180, ENSAMEG00000016820, ENSAMEG00000017184, ENSAMEG00000017745	CBFA2T3, PPARG, HAX1, GATA2, IL25, APP, BATF2
GO:0007626	locomotory behavior	biological process	7/269	115/13500	0.00814	0.882	ENSAMEG00000003627, ENSAMEG00000008061, ENSAMEG00000015157, ENSAMEG00000016396, ENSAMEG00000016625, ENSAMEG00000017184, ENSAMEG00000017191	EPS8, SOBP, GAA, DLG4, PBX3, APP, ENSAMEG00000017191
GO:0033138	positive regulation of peptidyl-serine phosphorylation	biological process	5/269	63/13500	0.0083	0.882	ENSAMEG00000004441, ENSAMEG00000011750, ENSAMEG00000016268, ENSAMEG00000017062, ENSAMEG00000017184	GAS6, HAX1, CAMK1, ARRB1, APP
GO:0002437	inflammatory response to antigenic stimulus	biological process	3/269	22/13500	0.0091	0.882	ENSAMEG00000012590, ENSAMEG00000014490, ENSAMEG00000019893	GPX1, IL5RA, CYSLTR1
GO:0050701	interleukin-1 secretion	biological process	3/269	22/13500	0.0091	0.882	ENSAMEG00000004441, ENSAMEG00000016224, ENSAMEG00000016947	GAS6, CD36, NLRP3
GO:0009117	nucleotide metabolic process	biological process	15/269	381/13500	0.00953	0.882	ENSAMEG00000000272, ENSAMEG00000003519, ENSAMEG00000006082, ENSAMEG00000007497, ENSAMEG00000008034, ENSAMEG00000010857, ENSAMEG00000011455, ENSAMEG00000011494, ENSAMEG00000014118, ENSAMEG00000015964, ENSAMEG00000017990, ENSAMEG00000023430, ENSAMEG00000023436, ENSAMEG00000023442, ENSAMEG00000023455	CBFA2T3, PFKFB2, RHOQ, NADSYN1, ENSAMEG00000008034, ADCY9, PDE4A, FBP1, ENSAMEG00000014118, ADCY4, BAD, ND2, COX1, ATP6, CYTB

## Discussion

Ageing mammals in captivity often develop cataracts due to the accumulation of oxidative damage [1]. This has been observed in captive giant pandas, which typically live up to 10 years longer than their wild counterparts [5, 6]. The prevalence of cataracts in the current population of aged giant pandas is ~ 20% and this is associated with a declining quality of life, as the animals find it difficult to feed and negotiate their surroundings. Although genetic factors have been identified that promote age-related cataracts, the most important triggers are environmental, particularly oxidative stress and DNA damage [7–10]. We previously compared the DNA methylation status of giant pandas with and without cataracts in an attempt to identify epigenetic effects that might influence the expression of genes associated with cataract formation [17]. We identified multiple differentially methylated genes with potential roles in cataract-related pathways, including base excision repair, apoptosis and p53 signaling. Certain genes also showed abnormal methylation profiles specifically in the pandas with cataracts, including the cysteine-aspartate protease gene *CASP3*, a pro-apoptotic mediator already linked to cataracts in rats [18, 19], and the glutathione S-transferase gene *GSTM3*, which is expressed in the lens tissues of human patients with age-related cataracts [20].

DNA methylation is an epigenetic mechanism that regulates gene expression by modifying the structure of chromatin, usually leading to the suppression of gene expression. As a logical extension of our previous study, we were therefore interested in the analysis of differential gene expression between giant pandas with cataracts and controls with healthy eyes. Using the same animals as in our previous study, we carried out RNA-Seq analysis to identify panels of genes either upregulated or downregulated in pandas with cataracts. Following the alignment of reads with the reference genome, we identified expression profiles representing genes expressed exclusively or preferentially in the cataractogenic or healthy samples, and identified the corresponding functional annotations by screening the KEGG database and GO categories. Our RNA-Seq data also revealed abundant alternative splicing and novel transcripts within the dataset, as previously reported during murine lens development [21]. Two genes that were shown in our DNA methylation study [17] to be methylated specifically in affected pandas were also shown to be downregulated in the affected pandas by RNA-Seq analysis. CASP3, encoding the apoptotic cysteine-aspartate protease was downregulated with a log_2_FC of − 1.03, whereas GSTM3, encoding the glutathione S-transferase, was downregulated with a log_2_FC of − 1.28.

A link between the glutathione (GSH) pathway and cataract formation was identified in a transcriptomic study in mice [22]. The authors considered the consequences of GSH deficiency in naïve and buthionine sulfoximine-treated C57Bl/6 LEGSKO (lens GSH-synthesis knockout) mice compared to wild-type controls, thus providing a more relevant model of oxidative damage. Among 24,415 mapped reads, 441 genes showed significantly modulated expression, including genes involved in epithelial-mesenchymal transition (EMT) signaling, the visual cycle, and lipid metabolism. Several detoxification genes were upregulated, including the aldehyde dehydrogenases *Aldh1a1* and *Aldh3a1*, the metallothioneins *Mt1* and *Mt2*, the carboxylesterase *Ces1g*, and the urea transporter *Slc14a1*, whereas genes encoding lens crystallins and other vision-related genes were downregulated [22]. The authors concluded that GSH deficiency in the lens leads to the expression of detoxifying genes and the activation of EMT signaling (providing evidence of adaption to the loss of antioxidant capacity) and also revealing a pathogenetic mechanism of cataract formation.

Similarly, our RNA-Seq analysis revealed the differential expression of several genes related to oxidative damage, the visual cycle, developmental functions, and lipid metabolism, suggesting that ageing pandas develop cataracts as the natural capacity for oxidative stress responses begins to diminish. For example, we observed a 7.45-fold downregulation of *NCF1*, encoding a membrane-bound subunit of NADPH oxidase which is involved in superoxide production and the induction of apoptosis [23, 24]. Examples of differentially regulated genes involved in visual perception included *COL2A1*, encoding a collagen component of the extracellular matrix (1.2-fold induction) and *UNC119*, encoding a G-protein-binding factor that is required for protein trafficking in photoreceptor cells (1.1-fold induction) [25]. The differentially expressed genes related to developmental functions included *EPCAM*, encoding the epithelial cell adhesion molecule involved in cell–cell adhesion, cell signaling, migration, proliferation, and differentiation (downregulated 1.1-fold) [26, 27] as well as a chloride channel that interacts with the cytoskeleton and is known to regulate vascular morphogenesis in the eye (*CLIC4*, 1.2-fold induction) [28, 29] and another gene involved in vascular remodeling (*ENG*, 2.1-fold induction) [30].

Previous transcriptomic studies of cataract formation have focused on the analysis of gene expression in models of human congenital cataracts rather than age-related cataract formation, but it is possible that some of the pathway elements are conserved. For example, mutations in the major intrinsic protein (MIP, also known as aquaporin 0) promote cataract formation in human infants, and several strains of rats and mice with loss-of-function *mip* mutations also develop fully penetrant cataracts [31–35]. More recently, we reported a novel mutation in the panda *Mip* gene also associated with cataract formation [36]. Transcriptomic analysis in juvenile *mip*^−/−^ knockout mice identified 29 genes with > 2-fold changes in expression, including the mitochondrial translocase (*Timmdc1*), a matrix metallopeptidase (*Mmp2*), a Rho GTPase-interacting protein (*Ubxn11*) and a transcription factor (*Twist2*) which were strongly upregulated, and a proteasome subunit (*Psmd8*), a ribonuclease (*Pop4*), and a heat-shock protein (*Hspb1*) that were strongly downregulated [37].

The discovery of differentially expressed proteasome subunits and heat shock factors indicates that the regulation of protein turnover may contribute to cataract formation, and likewise we identified several differentially expressed genes with similar functions. These included *GLG1*, which encodes a negative regulator of protein processing (downregulated 5.7-fold in affected pandas), *AZIN1*, which encodes a regulator of protein turnover (downregulated 1.2-fold in affected pandas), and *UCHL1*, which encodes a thiol-dependent ubiquitin-specific protease (downregulated 1.8-fold in affected pandas). Furthermore, we detected the strong (16.4-fold) upregulation of *TNFSF12*, encoding a regulator of protein turnover linked to angiogenesis and apoptosis [38], and a 2.1-fold induction of *SERPINB10*, another regulator of proteases associated with apoptosis [39]. The link with apoptosis was also supported by the 3.7-fold induction of *EGR1*, which mediates p53-independent apoptosis induced by c-Myc [40]. We also observed the 1.5-fold induction of *ARHGAP21*, a target of p53 that promotes the degradation of MDM2 to maintain TP53 stability [41], and the 1.4-fold induction of *STEAP3*, another target of p53 that regulates cell cycle progression and apoptosis [42]. The *IFI27L2* gene, encoding an interferon-induced mitochondrial membrane protein that also promotes apoptosis, was induced 1.24-fold [43]. Recent studies have shown that lens epithelial cells undergo apoptosis as a common cytological basis for all kinds of cataract except congenital lesions.

Several of the most strongly modulated genes we identified are involved in signaling, indicating the activation of cell–cell signaling as a response to lens deterioration. The top-ranking downregulated gene was *RCAN1* (16-fold repression), which encodes a calcineurin-binding regulator of CNS development. The relevance of this gene in cataracts is unclear, but may be linked to the vascular remodeling discussed above given its role in the suppression of angiogenesis [44]. Other genes we identified appear to be involved in signaling pathways involving G-protein-coupled receptors (GPCRs). For example, *CYSLTR1* (induced 1.3-fold) encodes a leukotriene-specific GPCR typically involved in bronchoconstriction, but it also regulates vascular permeability, cell migration and collagen deposition, which may be the more relevant functions here [45]. The UV irradiation of lens epithelial cells was previously shown to increase the expression of LET-7B, the ligand for another GPCR (LGRT), resulting in the induction of apoptosis [46]. A targeted deletion in *LGR4* reduced the resistance of rat lens epithelial cells to oxidative stress and accelerated the development of age-related cataracts [47]. *RASD1* (induced 4.3-fold) encodes a member of the Ras family of G-protein regulators acting downstream of GPCRs, and its upregulation in human cell lines has been shown to suppress cell growth and induce apoptosis [48, 49]. Similarly *RANBP9* (induced 2.7-fold) encodes a Ras protein that influences cytoskeletal organization and interacts with several regulators of cell growth [50]. *PDE4A* (induced 1.6-fold) encodes a cAMP-specific 3′,5′-cyclic phosphodiesterase that regulates second messenger signaling downstream of GPCRs by cleaving cAMP [51]. Other modulated genes encoding signaling proteins potentially involved in the regulation of cell growth included *PTP4A3*, encoding a membrane-associated protein tyrosine phosphatase (induced 2.8-fold) [52], *ANXA3*, encoding annexin 3 (downregulated 1.75-fold) [53], and *DUSP22*, encoding a dual-specificity protein kinase (induced 1.42-fold) [54]. We also observed the 1.92-fold downregulation of the *MYBL1* gene, whose principal function is the regulation of meiosis, so its relevance in the context of cataract formation is unclear.

Mutations in the *HSF4* gene have been shown to cause cataracts in humans and other mammals [55], including the recent discovery of a novel *HSF4* mutation in pandas [56]. HSF4 is a transcriptional repressor and we proposed that the novel mutation we discovered in this gene is likely to affect its interactions with upstream signaling components, thus disrupting the genetic control of lens functions. Although *HSF4* was not among the differentially expressed genes we detected in this study, we found that both FOS and FOSB were strongly upregulated (4.5-fold and 4.9-fold, respectively). These genes encode two leucine zipper proteins that dimerize with members of the JUN family to form transcriptional regulators [57]. Importantly, the FOS family of transcription factors is involved in the regulation of many of the processes discussed above revealed by the functional analysis of other differentially expressed genes, including cell proliferation, differentiation and survival, oxidative stress and angiogenesis [58]. It is therefore possible that FOS/FOSB play a key role in the regulation of the other genes discussed above, or that the *FOS*/*FOSB* genes are targets of the modulated signaling pathways we have identified.

The analysis of GO enrichment provided results that were broadly consistent with the functional annotation of the differentially expressed genes, with *cellular process*, *metabolic process*, *biological regulation*/*regulation of a biological process* as the most overrepresented categories in the differentially expressed gene catalog. The categories *immune system process* and *multi-organism process* were also represented, which may reflect the modulation of signaling proteins that are also known to participate in the regulation of immunity responses, including cytokine release and immune cell recruitment. Another enriched category was *protein-containing complex*, which agreed with our preliminary protein network analysis and is consistent with the tendency of signaling proteins to form complexes. Finally, functional annotation by screening the differentially expressed genes against the KEGG database generated several hits in the histidine metabolic pathway, which is particularly interesting given that dietary histidine and/or carnitine are known to prevent cataract development in salmon, presumably by countering the effect of oxidative stress [59, 60]. Taken together, our results confirm that age-related cataracts in pandas bear many of the same hallmarks as cataracts in other animals, including the modulation of stress response genes, metabolic adaptations, and signaling pathways regulating cell growth and apoptosis. The identification of genes that are overexpressed or suppressed during the formation of cataracts could lead to the development of markers for early diagnosis, preventative strategies and therapies that improve the quality of life for captive giant pandas and other mammals.

## Conclusions

Blood samples from six giant pandas with and without cataracts were used for de novo RNA-Seq analysis. This revealed the differential expression of several genes related to oxidative damage, the visual cycle, developmental functions, and lipid metabolism, suggesting that ageing pandas may develop cataracts as the natural capacity for oxidative stress responses begins to diminish. We found that many of the genes encoded regulatory and signaling proteins associated with the control of cell growth, migration, differentiation and apoptosis, supporting previous research indicating a key role for apoptosis in cataract formation. The identification of genes potentially involved in the formation of age-related cataracts could facilitate the development of predictive markers, preventative measures and even new therapies to improve the life of captive animals.

## Methods

### Clinical findings

Routine physical examinations were carried out every month on the living captive animals, including eye, mouth and nose and general physical appearance, abdominal palpation, and general clinical signs. Blood was collected once a month for the analysis of physiological and biochemical indicators in order to exclude risk factors such as injury, diabetes or other diseases that can promote cataract formation [36].

### RNA isolation

Peripheral blood samples (2 ml) were collected from all six giant panda specimens (Table 1) because markers for many diseases can be detected in blood using transcriptomics methods [61] including eye diseases [62–64]. JN and LL were from Beijing Zoo, XX and YE were from Chongqing Zoo, and BD and YY were from Strait (Fuzhou) Giant Panda Research and Exchange Center. Three of the females were diagnosed with age-related cataracts and the other three donors were healthy controls. Blood samples were stored at 4 °C and total RNA was extracted within 2 days using the Trizol Total RNA Extractor kit (Sangon Biotech, Shanghai, China) according to the manufacturer’s protocol. The samples were treated with RNase-free DNase I to remove genomic DNA. RNA integrity was evaluated by 1.0% agarose gel electrophoresis, and RNA quality and quantity were assessed using a NanoPhotometer (Implen, Westlake Village, CA, USA) and an Agilent 2100 Bioanalyzer (Agilent Technologies, Santa Clara, CA, USA).

### Library preparation and sequencing

High-quality RNA samples were sent to Sangon Biotech for library preparation and sequencing. The libraries were generated from 2 μg RNA using the VAHTS mRNA-seq V2 Library Prep Kit for Illumina (Vazyme Biotech, Nanjing, China) and index codes were added to attribute sequences to each sample. Briefly, mRNA was purified from total RNA using poly-T oligo-attached magnetic beads. Fragmentation was carried out using divalent cations at 94 °C in VAHTS 5× First Strand Synthesis Reaction Buffer, and first-strand cDNA was synthesized using random hexamers and M-MuLV reverse transcriptase RNase H^−^. Second-strand cDNA was synthesized using DNA polymerase I and RNase H, which also created blunt ends for 3′ polyadenylation. Following adapter ligation, cDNA fragments in the size range 150–200 bp were selected using the AMPure XP system (Beckman Coulter, Brea, CA, USA). The size-selected and adaptor-ligated cDNA was incubated with 3 μL USER enzyme mix (New England Biolabs, Ipswich, MA, USA) for 15 min at 37 °C then 5 min at 95 °C. We then carried out PCR using Phusion high-fidelity DNA polymerase, universal PCR primers i7 (5′-CAA GCA GAA GAC GGC ATA CGA GAT-index primer-GTG ACT GGA GTT CAG ACG TGT GCT C-3′) and i5 (5′-A*A TGA TAC GGC GAC CAC CGA GAT CTA CAC-index primer-ACA CTC TTT CCC TAC ACG ACG CTC TTC CGA T*C-3′) and sample-specific index primers (Table 4). The PCR products were purified (AMPure XP system) and library quality was assessed using the Agilent Bioanalyzer 2100 system. The libraries were then quantified and pooled. Paired-end sequencing was carried out using a HiSeq XTen device (Illumina, San Diego, CA, USA).

**Table 4:** Sample-specific index primers for sequencing

Name	Index primer (5′ → 3′)	
	i7	i5
Bing-Dian	ACTCGGTA	TGCAGAGC
Ya-Ya	CGTAAGCC	CGTAAGCC
Ya-Er	CTCATGTC	GTAGACGT
Ji-Ni	GACTGTCA	TCAGTCAC
Le-Le	CTGCCTGT	CAGTAGAC
Xin-Xing	TCAGTCAC	ATGCAGGT

### Data assessment and quality control

The quality of the sequence data was determined using FastQC v0.11.2. Raw reads were filtered using Trimmomatic v0.36 in five steps. First, the adaptor sequences were removed. Next, low-quality bases (Q < 20) were removed from reads in the 3′ to 5′ direction and then in the 5′ to 3′ direction. Then we used a sliding window method to remove low-quality bases from the read tails (window size = 5 bp). Finally, any reads smaller than 35 nt were removed along with the corresponding paired reads.

### Alignment with reference genome

Sequences randomly selected from clean data were used as blastn queries against the NCBI nucleotide database (http://ncbi.nlm.nih.gov/). Hits with an E-value cutoff of ≤1 × 10^− 10^, similarity > 90% and coverage > 80% of the results were used to calculate the species distribution and pollution detection. The remaining clean reads were mapped to the reference genome using HISAT2 v2.0 with default parameters. RSeQC v2.6.1 was used for the statistical analysis of the alignment results.

### Gene structure analysis

The sequence of each chromosome was compared and mapped statistically to show the distribution of sequences using BEDtools v2.26.0. After comparing the reads to the reference genome, the proportion of each gene structure in the reads was counted, including exons, introns and intergenic DNA, using Qualimap v2.2.1. Only 30 chromosomes were displayed. BCFtools v1.5 was used to find SNPs and SnpEff v2.36 was used to determine their effects. The sequences were filtered according to the mass value (> 20) and coverage (> 8). The sequences were assembled using StringTie v1.3.3b, and compared with existing genomic data in the STRING database (http://string-db.org/) using GffCompare v0.10.1 to find new transcription regions. The predicted transcripts were used as gene models to identify variable splicing. ASprofile v1.0.4 is used to classify variable splicing events according to the predicted gene model of each sample.

### Expression analysis

StringTie v1.3.3b was applied to the transcriptionally assembled reference genome to determine gene expression levels by measuring transcript abundances as transcripts per million reads (TPM) for both the protein-coding genes and lncRNAs in each sample. After quality control, the sequence was compared with the reference genome using HISAT2 v2.1.0 and the results were statistically compared using RSEQC v2.6.1, with the feature count used to homogenize the read count matrix (gene length and sequence depth) after each gene count to determine the TPM. Principal component analysis (PCA) and principal co-ordinates analysis (PCoA) were used to determine the distance and difference between samples in vegan v2.0.10. Data were presented as TPM avoid the influence of gene length or sequencing discrepancies during sample comparison. We used DESeq2 v1.12.4 to identify genes that were differentially expressed between two samples. Differential expression was considered significant if the false discovery rate (q-value) was ≤0.05 and the log2 fold change (|FC| value) was ≥2. If the normalized expression of a gene between two samples was zero, its expression value was adjusted to 0.01 (because 0 cannot be plotted on a log plot). If the normalized expression of a gene in two libraries was < 1, further differential expression analysis was conducted without this gene.

### Functional analysis of differentially expressed genes

Given the absence of biological replicate samples, we first standardized all the data with TMM. This removed all the unexpressed genes, identified a sample with average data trends from many samples as the reference sample, calculated the total number of reads for all samples, and divided each sample by its own total number of reads to get the modified number of reads. The Q3 value (the third quartile) of each sample’s modified read number was calculated, averaged, and the sample with the smallest difference from the average Q3 was used as the reference sample. To find the representative gene sets in each sample and calculate the standardization factor of the sample, we referred to the fold change of these representative gene sets. Differentially expressed genes were identified by standardizing the read count data in DESeq2 v1.12.4 as described above. TopGO v2.24.0 was then used for GO enrichment analysis and to prepare the significant GO-directed acyclic graph. ClusterProfiler v3.0.5 was used to analyze KEGG pathways and KOG taxonomic enrichment analysis, allowing the construction of a network diagram. Functional enrichment analysis was carried out to identify differentially expressed genes significantly enriched in GO terms (biological, cellular and molecular functions) or KEGG metabolic pathways. Genes were mapped to the GO database (http://www.geeontology.org) and KEGG database (http://www.kegg.jp) [65–67], the number of genes representing each term or pathway was calculated, and hypergeometric tests were performed to identify significantly enriched GO terms or KEGG pathways in the gene list. GO terms and KEGG pathways were considered significant if the q-value was ≤0.05.

The results of differential gene expression analysis were visualized using DESeq2 v1.12.4 and mapped to the STRING protein interaction network database (http://string-db.org/) to construct the protein interaction network. Based on these results, Venn diagrams were prepared with VennDiagram v1.6.17 in the R package. The correlation of gene expression levels between samples was determined in R using the cor.test package to ensure data reliability and rational sample selection. The sample-to-sample and group-to-group distance heat map were prepared in R using the gplots v2.17.0 package, which showed the distance relationship between the samples or the groups directly. Scatter plots were used to show the degree of difference between groups of genes and were prepared in R v3.2.

## Supplementary Information

**Additional file 1: Supplementary Table S1.** Alignment statistics. Total reads = number (percentage) of sequences in the clean data (after sequence filtering). Total mapped = number (percentage) of sequences that can be located on the reference sequence. Multiple mapped = number (percentage) of sequences with multiple comparison positions on the reference sequence. Unique mapped = number (percentage) of sequences with unique comparison positions on the reference sequence. Read-1/Read-2 mapped = number (percentage) of Read-1 and Read-2 sequences compared to reference sequence (only the unique mapped sequences were calculated). Reads mapped to +/− = the number (percentage) of positive and negative chains on the reference sequence compared to the sequencing sequence (only the unique mapped sequences were calculated). Non-splice reads = number (percentage) of all sequences compared to exons. Splice reads = number (percentage) of segmented comparisons of sequences (also known as junction reads) on two exons. Reads mapped in proper pairs = number (percentage) of sequences for simultaneous alignment of two terminal reads.

**Additional file 2: Supplementary Table S2.** New transcript prediction statistics.

**Additional file 3: Supplementary Table S3.** Correlation analysis between samples.

**Additional file 4: Supplementary Fig. S1.** Genome coverage statistics. (a) Homogeneity distribution curves. The x-axis represents the length of a gene, with 0 as the 5′ end and 100 as the 3′ end. The y-axis shows the total number of sequences that mapped to the corresponding gene position. Each color represents one sample. (b) Pie chart showing the gene coverage ratio. The percentage value represents the percentage of the total area of the corresponding gene in the region under which the gene is measured, with the number of genes that can be measured within the interval in parentheses. (c) Distribution of genome coverage by exon, intron and intergenic regions.

**Additional file 5: Supplementary Fig. S2.** Frequency of different types of single-nucleotide polymorphism, with red representing transversions and blue representing transitions.

**Additional file 6: Supplementary Fig. 3.** Directed acyclic graph of significant Gene Ontology molecular functions. Each box represents a GO term, showing the GO term ID, GO description, GO enriched *p*-value, and the number of differentially expressed/background genes under each GO term. The depth of color represents the degree of enrichment. (a) Acyclic graph of significant GO biological processes. (b) Acyclic graph of significant GO molecular functions. (c) Acyclic graph of significant GO cellular components.

## Data Availability

The RNA sequencing data are available at NCBI GenBank (https://dataview.ncbi.nlm.nih.gov/object/PRJNA720280?reviewer=m87rlugdrccp4b1f1q494083re). All data necessary for confirming the conclusions of the article are present within the article, figures, and tables.
