# Supplementary material for: RNA-Seq analysis in giant pandas reveals the differential expression of multiple genes involved in cataract formation
Source: BMC Genom Data. 2021 Oct 27;22:44. doi: 10.1186/s12863-021-00996-x (PMC8555103; doi:10.1186/s12863-021-00996-x)
Supplement: Supplementary file 1 — Additional file 1: Supplementary Table S1. Alignment statistics. Total reads = number (percentage) of sequences in the clean data (after sequence filtering). Total mapped = number (percentage) of sequences that can be located on the reference sequence. Multiple mapped = number (percentage) of sequences with multiple comparison positions on the reference sequence. Unique mapped = number (percentage) of sequences with unique comparison positions on the reference sequence. Read-1/Read-2 mapped = number (percentage) of Read-1 and Read-2 sequences compared to reference sequence (only the unique mapped sequences were calculated). Reads mapped to +/− = the number (percentage) of positive and negative chains on the reference sequence compared to the sequencing sequence (only the unique mapped sequences were calculated). Non-splice reads = number (percentage) of all sequences compared to exons. Splice reads = number (percentage) of segmented comparisons of sequences (also known as junction reads) on two exons. Reads mapped in proper pairs = number (percentage) of sequences for simultaneous alignment of two terminal reads. [file 12863_2021_996_MOESM1_ESM.docx]

**Supplementary Table S1** **Alignment result statistics**

|  | A1 | A3 | A4 | B1 | C1 | C2 |
| --- | --- | --- | --- | --- | --- | --- |
| Total reads | 46432410(100.00%) | 54837174(100.00%) | 61784706(100.00%) | 56589466(100.00%) | 45324992(100.00%) | 49949742(100.00%) |
| Total mapped | 42787208(92.15%) | 50797700(92.63%) | 57336104(92.80%) | 44791449(92.15%) | 41199486(90.90%) | 46389975(92.87%) |
| Mutiple mapped | 1036909(2.23%) | 1291261(2.35%) | 1467277(2.37%) | 1276634(2.26%) | 949090(2.09%) | 2714402(5.43%) |
| Uniquely mapped | 41750299(89.92%) | 49506439(90.28%) | 55868827(90.43%) | 43514815(76.90%) | 40250396(88.80%) | 43675573(87.44%) |
| Read-1 mapped | 20843264(44.89%) | 24766282(45.16%) | 27944853(45.23%) | 22106622(39.06%) | 20133234(44.42%) | 21829892(43.70%) |
| Read-2 mapped | 20907035(45.03%) | 24740157(45.12%) | 27923974(45.20%) | 21408193(37.83%) | 20117162(44.38%) | 21845681(43.74%) |
| Reads map to '+' | 20903669(45.02%) | 24747601(45.13%) | 27930515(45.21%) | 21791006(38.51%) | 20165870(44.49%) | 21854307(43.75%) |
| Reads map to '-' | 20846630(44.90%) | 24758838(45.15%) | 27938312(45.22%) | 21723809(38.39%) | 20084526(44.31%) | 21821266(43.69%) |
| Non-splice reads | 26227461(56.49%) | 28095348(51.23%) | 31134787(50.39%) | 26154115(46.22%) | 24843598(54.81%) | 26680246(53.41%) |
| Splice reads | 15522838(33.43%) | 21411091(39.04%) | 24734040(40.03%) | 17360700(30.68%) | 15406798(33.99%) | 16995327(34.02%) |
| Reads mapped in proper pairs | 38848602(83.67%) | 46591318(84.96%) | 52890678(85.60%) | 39092784(69.08%) | 37502490(82.74%) | 40818986(81.72%) |
