# Supplementary material for: RNA-Seq analysis in giant pandas reveals the differential expression of multiple genes involved in cataract formation
Source: BMC Genom Data. 2021 Oct 27;22:44. doi: 10.1186/s12863-021-00996-x (PMC8555103; doi:10.1186/s12863-021-00996-x)
Supplement: Supplementary file 2 — Additional file 2: Supplementary Table S2. New transcript prediction statistics. [file 12863_2021_996_MOESM2_ESM.docx]

**Supplementary Table S2** New transcripts prediction statistics

| **Isoform ID** | **Postion** | **Class Code** | **Gene Name** | **Compare Ref** |
| --- | --- | --- | --- | --- |
| MSTRG.39.2 | GL192338.1[+]2492995-2647419 | j | GNAO1 | ENSAMET00000018252 |
| MSTRG.46.1 | GL192338.1[+]2722379-2745820 | j | OGFOD1 | ENSAMET00000018310 |
| MSTRG.51.1 | GL192338.1[+]3028598-3028879 | o | SLC12A3 | ENSAMET00000018456 |
| MSTRG.53.2 | GL192338.1[+]3042086-3053028 | j | HERPUD1 | ENSAMET00000018469 |
| MSTRG.53.1 | GL192338.1[+]3042086-3053031 | j | HERPUD1 | ENSAMET00000018469 |
| MSTRG.53.3 | GL192338.1[+]3044659-3052963 | j | HERPUD1 | ENSAMET00000018469 |
| MSTRG.88.2 | GL192338.1[+]3078548-3169624 | j | NLRC5 | ENSAMET00000018521 |
| MSTRG.88.1 | GL192338.1[+]3078548-3216532 | j | NLRC5 | ENSAMET00000018521 |
