# Supplementary material for: RNA-Seq analysis in giant pandas reveals the differential expression of multiple genes involved in cataract formation
Source: BMC Genom Data. 2021 Oct 27;22:44. doi: 10.1186/s12863-021-00996-x (PMC8555103; doi:10.1186/s12863-021-00996-x)
Supplement: Supplementary file 3 — Additional file 3: Supplementary Table S3. Correlation analysis between samples. [file 12863_2021_996_MOESM3_ESM.docx]

|  | A1 | A2 | A3 | B1 | C1 | C2 |
| --- | --- | --- | --- | --- | --- | --- |
| A1 | 1 | 0.788862 | 0.773886 | 0.971615 | 0.932963 | 0.715734 |
| A2 | 0.788862 | 1 | 0.998445 | 0.705802 | 0.894367 | 0.373031 |
| A3 | 0.773886 | 0.998445 | 1 | 0.69325 | 0.88581 | 0.363586 |
| B1 | 0.971615 | 0.705802 | 0.69325 | 1 | 0.876755 | 0.80911 |
| C1 | 0.932963 | 0.894367 | 0.88581 | 0.876755 | 1 | 0.506693 |
| C2 | 0.715734 | 0.373031 | 0.363586 | 0.80911 | 0.506693 | 1 |

**Supplementary Table S3 Correlation analysis statistical table between samples**
