# Supplementary figures and images for: RNA-Seq analysis in giant pandas reveals the differential expression of multiple genes involved in cataract formation
Source: BMC Genom Data. 2021 Oct 27;22:44. doi: 10.1186/s12863-021-00996-x (PMC8555103; doi:10.1186/s12863-021-00996-x)

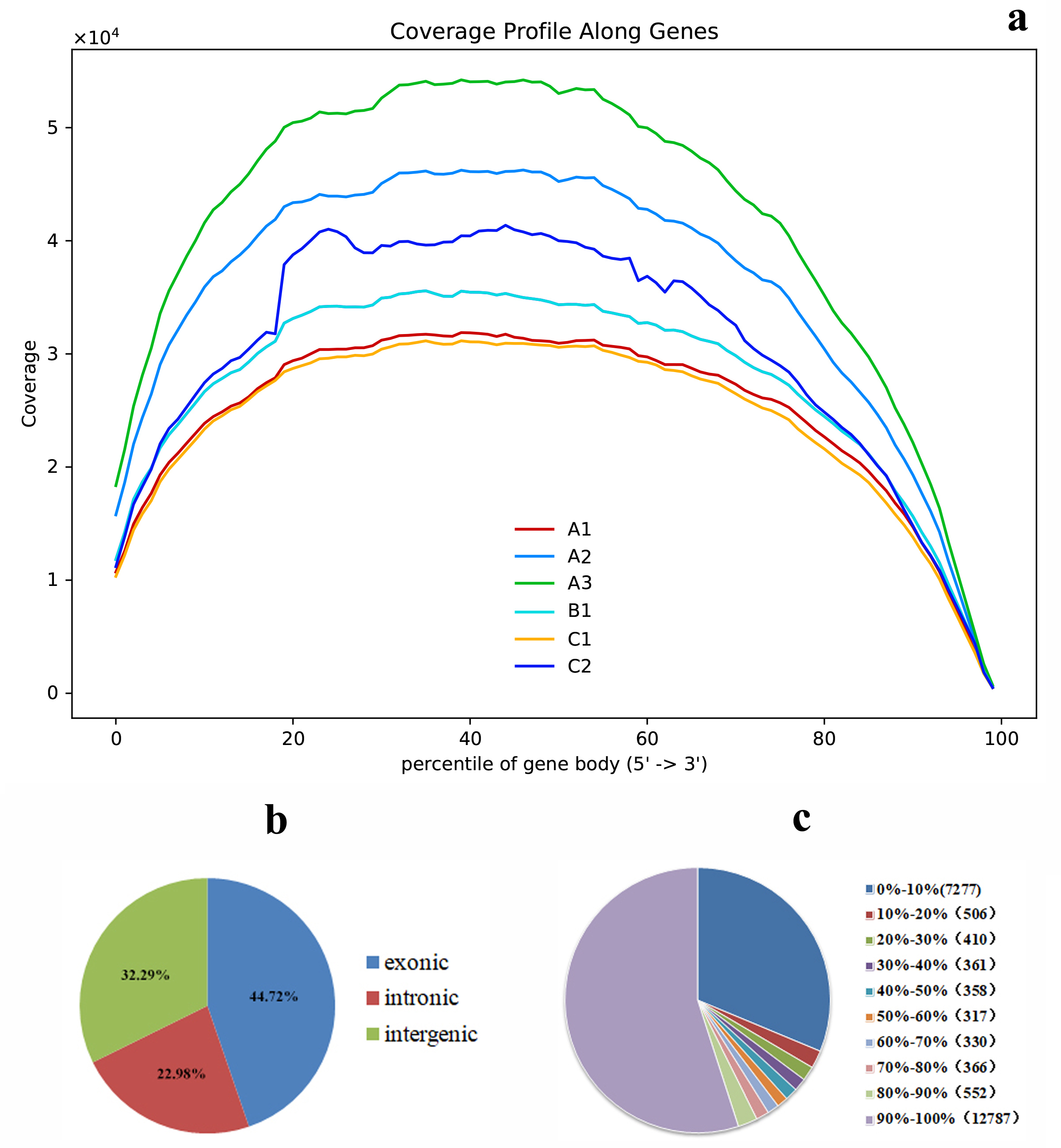

Supplement: Supplementary file 4 — Additional file 4: Supplementary Fig. S1. Genome coverage statistics. (a) Homogeneity distribution curves. The x-axis represents the length of a gene, with 0 as the 5′ end and 100 as the 3′ end. The y-axis shows the total number of sequences that mapped to the corresponding gene position. Each color represents one sample. (b) Pie chart showing the gene coverage ratio. The percentage value represents the percentage of the total area of the corresponding gene in the region under which the gene is measured, with the number of genes that can be measured within the interval in parentheses. (c) Distribution of genome coverage by exon, intron and intergenic regions. [file 12863_2021_996_MOESM4_ESM.jpg]

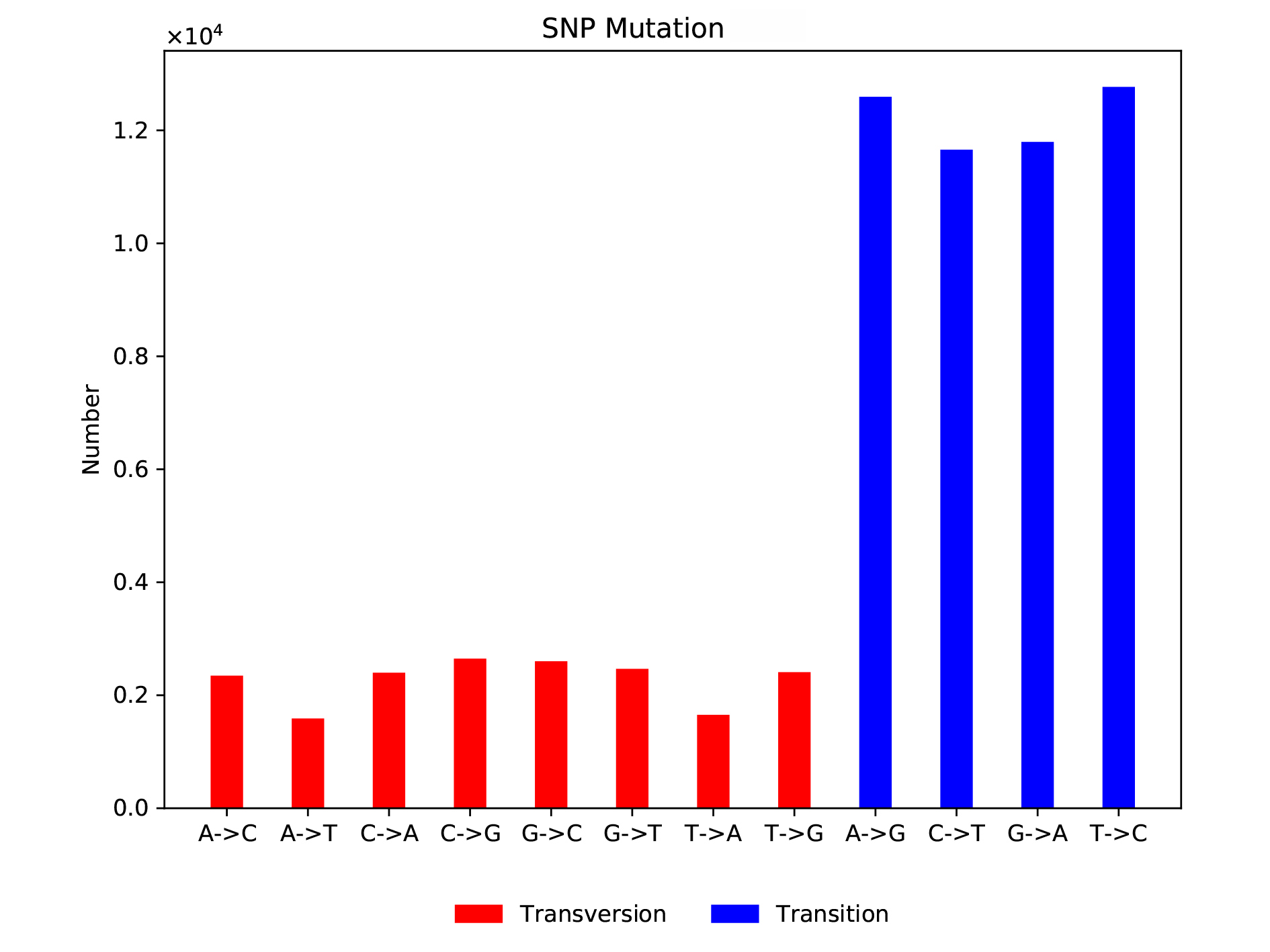

Supplement: Supplementary file 5 — Additional file 5: Supplementary Fig. S2. Frequency of different types of single-nucleotide polymorphism, with red representing transversions and blue representing transitions. [file 12863_2021_996_MOESM5_ESM.jpg]

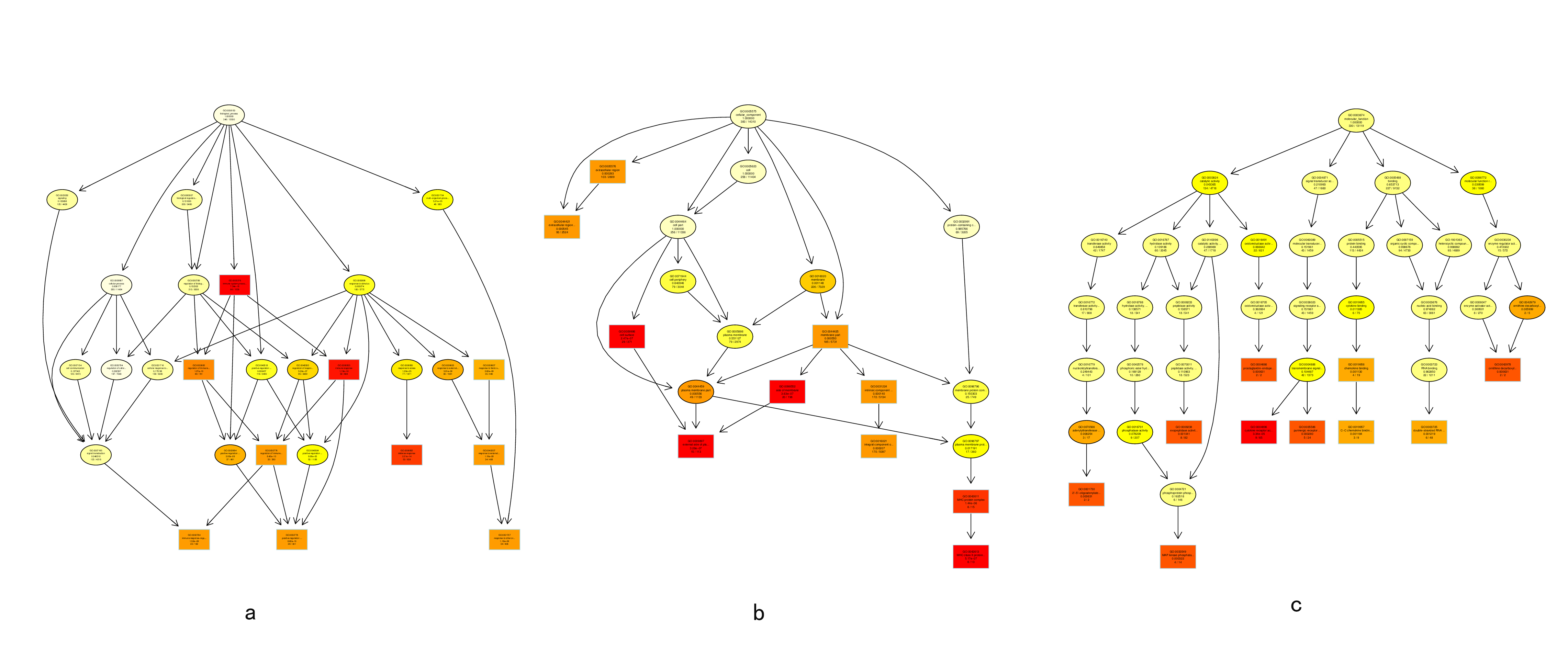

Supplement: Supplementary file 6 — Additional file 6: Supplementary Fig. 3. Directed acyclic graph of significant Gene Ontology molecular functions. Each box represents a GO term, showing the GO term ID, GO description, GO enriched p-value, and the number of differentially expressed/background genes under each GO term. The depth of color represents the degree of enrichment. (a) Acyclic graph of significant GO biological processes. (b) Acyclic graph of significant GO molecular functions. (c) Acyclic graph of significant GO cellular components. [file 12863_2021_996_MOESM6_ESM.jpg]
